# Complex population structure of the Atlantic puffin revealed by whole genome analyses

**DOI:** 10.1038/s42003-021-02415-4

**Published:** 2021-07-29

**Authors:** Oliver Kersten, Bastiaan Star, Deborah M. Leigh, Tycho Anker-Nilssen, Hallvard Strøm, Jóhannis Danielsen, Sébastien Descamps, Kjell E. Erikstad, Michelle G. Fitzsimmons, Jérôme Fort, Erpur S. Hansen, Mike P. Harris, Martin Irestedt, Oddmund Kleven, Mark L. Mallory, Kjetill S. Jakobsen, Sanne Boessenkool

**Affiliations:** 1 Centre for Ecological and Evolutionary Synthesis (CEES), Department of Biosciences, University of Oslo, Oslo, Norway; 2 WSL Swiss Federal Research Institute, Birmensdorf, Switzerland; 3 Norwegian Institute for Nature Research (NINA), Trondheim, Norway; 4 Norwegian Polar Institute, Fram Centre, Langnes, Tromsø Norway; 5 Faroe Marine Research Institute (FAMRI), Tórshavn, Faroe Islands; 6 Norwegian Institute for Nature Research (NINA), Fram Centre, Langnes, Tromsø Norway; 7 Centre for Biodiversity Dynamics (CBD), Norwegian University of Science and Technology (NTNU), Trondheim, Norway; 8 Environment and Climate Change Canada, Newfoundland and Labrador, Canada; 9 Littoral, Environment et Sociétés (LIENSs), UMR 7266 CNRS—La Rochelle Université, La Rochelle, France; 10 South Iceland Nature Research Centre, Ægisgata 2, Vestmannaeyjar, Iceland; 11 UK Centre for Ecology & Hydrology, Penicuik, Midlothian UK; 12 Department of Bioinformatics and Genetics, Swedish Museum of Natural History, Stockholm, Sweden; 13 Department of Biology, Acadia University, Wolfville, Nova Scotia Canada

**Keywords:** Conservation genomics, Population genetics, Conservation biology, Genome

## Abstract

The factors underlying gene flow and genomic population structure in vagile seabirds are notoriously difficult to understand due to their complex ecology with diverse dispersal barriers and extensive periods at sea. Yet, such understanding is vital for conservation management of seabirds that are globally declining at alarming rates. Here, we elucidate the population structure of the Atlantic puffin (*Fratercula arctica*) by assembling its reference genome and analyzing genome-wide resequencing data of 72 individuals from 12 colonies. We identify four large, genetically distinct clusters, observe isolation-by-distance between colonies within these clusters, and obtain evidence for a secondary contact zone. These observations disagree with the current taxonomy, and show that a complex set of contemporary biotic factors impede gene flow over different spatial scales. Our results highlight the power of whole genome data to reveal unexpected population structure in vagile marine seabirds and its value for seabird taxonomy, evolution and conservation.

## Abstract (web summary)

Kersten et al. sequence a draft genome for the Atlantic puffin and report its population structure, genetic diversity and gene flow among four main clusters of populations across the northern Atlantic. These results identify a secondary contact zone between the puffins from the High Arctic and other colonies and proposes a new population structure from the currently recognized three subspecies.

## Introduction

Seabirds are important ecosystem indicators and drivers^1–3^, and have long had an integral place in human culture and economy^4–6^. Nevertheless, global seabird numbers have deteriorated by an alarming 70% since the mid-20th century^7,8^. These declines pose a serious threat to marine ecosystems, human society, and culture^7,9,10^, highlighting the importance of seabird conservation management. Within such management, the identification of distinct population units, i.e., demographically independent populations with restricted gene flow among them^11,12^, is a fundamental first step towards optimized conservation^11,13,14^. Defining such units is, however, difficult for many seabirds because of their complex ecology^15^. Detailed genomic data including thousands of loci provide new possibilities to assess levels of connectivity and gene flow between distinct breeding populations and, thus, help identify relevant conservation units for seabirds^15,16^. Indeed, a few recent publications using reduced genomic representation approaches (e.g., RAD-seq) have reported fine-scale structure over various spatial scales^17–21^. These studies highlight the great potential of genomic data to disentangle barriers to gene flow that would otherwise remain undetected, but remain nonetheless limited due to incomplete sampling of the genome^22^.

**Fig. 1:**
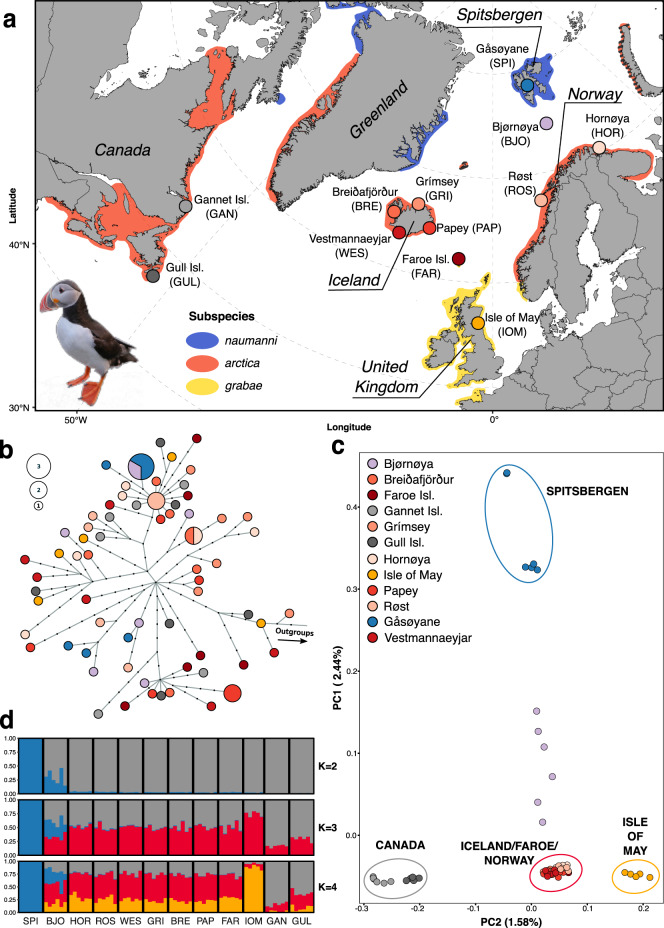
Sampling distribution and genomic structure of 71 Atlantic puffin individuals across 12 colonies throughout the breeding range. **a** Map presenting the location of the 12 sampling sites. Color shading indicates the breeding range of the species as a whole, as well as the recognized subspecies. **b** Mitochondrial haplotype network based on a maximum likelihood tree generated with IQTree and visualized using Fitchi. It contains 66 unique haplotypes identified by 192 mitogenome-wide SNPs. Sizes of circles are proportional to haplotype abundance. Color legend is provided in (**c**). Black dots represent inferred haplotypes that were not found in the present sampling. **c** Principal component analysis (PCA) using genotype likelihoods at 1,093,765 polymorphic nuclear sites calculated in ANGSD to project the 71 individuals onto PC axes 1 and 2. Each circle represents a sample and colors indicate the different colonies. The percentage indicates the proportion of genomic variation explained by each axis. The color coding of the colonies is consistently used throughout the manuscript. **d** CLUMPAK-averaged admixture plots of the best K’s using the same genotype likelihood panel as in (**c**). Each column represents a sample and colonies are separated by solid white lines. Optimal K’s were determined by the method of Evanno et al.^41^ (see Fig. S6a) and colors indicate the ancestry fraction to the different clusters. The dataset(s) needed to create this figure can be found at 10.6084/m9.figshare.14743242.v1.

The Atlantic puffin (*Fratercula arctica*, Linnaeus, 1789, hereafter “puffin”) is an iconic seabird species, prevalent in popular culture^23^, important for tourism^24,25^, and inherently valuable for the marine ecosystem^1^. Puffins were historically widely harvested for their meat and down^6,26,27^ and exploitation remains an important cultural tradition in Iceland and the Faroe Islands^6,24^. Its breeding range stretches from the Arctic coast and islands of European Russia, Norway, Greenland, and Canada, southward to France and the USA^28^ (Fig. 1a). Puffins have been designated as “vulnerable” to extinction globally and listed as “endangered” in Europe^29^. Notably, the once world’s largest puffin colony (Røst, Norway) has experienced complete fledging failure during nine of the last 13 seasons and has lost nearly 80% of its breeding pairs during the last 40 years^29–31^. Similarly, Icelandic and Faroese puffins have experienced low productivity and negative population growth since 2003^32^.

Puffins have been broadly classified into three taxonomic groups along a latitudinal gradient based on size, with the *smallest* puffins found around France, Britain, Ireland and southern Norway (*F. a. grabae*), *intermediate* sized puffins around Norway, Iceland, and Canada (*F. a. arctica*) and the *largest* puffins found in the High Arctic, e.g. Spitsbergen^33^, Greenland^34^, and northeastern Canada^35^ (*F. a. naumanni*)^36^ (Fig. 1a). Nevertheless, this broad classification into three subspecies has been controversial^28,37,38^ and the population structure of puffins remains unresolved at all spatial scales^37^. This knowledge gap obstructs efforts towards an assessment of dispersal barriers, limits our understanding of cause-and-effect dynamics between population trends, ecology and the marine ecosystem, and hinders the development of adapted large-scale conservation actions.

Here, we present the, to the best of our knowledge, first whole-genome analysis of structure, gene flow, and taxonomy of a pelagic, North Atlantic seabird. We generated a de novo draft assembly for the puffin and resequenced 72 individuals across 12 colonies representing the majority of the species’ breeding range (Fig. 1a). Our work suggests that a complex interplay of ecological factors contributes to the range-wide genomic population structure of this vagile seabird.

## Results

### Genome assembly and population sequencing

Based on synteny with the razorbill (*Alca torda*), a total of 13,328 puffin scaffolds were placed into 26 pseudo-chromosomes, leaving 17.06 Mbp (1.4%) unplaced and yielding an assembly of 1.294 Gbp (Supplementary Data 1, Table S1). This assembly contains 4,522 of the 4,915 genes (92.0%) of complete protein-coding sequences from the avian set of the OrthoDB v9 database (Supplementary Data 1). We also assembled the puffin mitogenome (length of 17,084 bp) with a similar arrangement of genomic elements as other members within the Alcidae^39,40^ (Fig. S1, Table S2). For the 72 resequenced specimens, we analyzed a total of 5.77 billion paired reads, obtaining an average fold-coverage of 7X (range 3.0–10) for the nuclear genome and 591X (5.3–1800) for the mitochondrial genome per specimen (Fig. 1a, Supplementary Data 2). One individual (IOM001) was removed from both datasets (nuclear and mitochondrial) due to a substantially lower number of mapped reads (endogeny) relative to all other samples (Supplementary Data 2) resulting in a large proportion of missing sites (Fig. S2). Additional filtering produced a final genotype likelihood dataset of 1,093,765 polymorphic nuclear sites and 192 mitochondrial single-nucleotide polymorphisms (SNPs, Supplementary Data 3) in 71 birds (36 males and 35 females).

### Genomic population structure

Genomic variation across 71 puffin mitogenomes defines 66 polymorphic haplotypes that indicate a recent global population expansion and show no significant population structure (Fig. 1b, Figs. S3, S4, Tables S3, S4). In contrast, we inferred four main population clusters using principal component analysis (PCA) of the nuclear whole-genome dataset (Fig. 1c). Puffins from Spitsbergen are most distinct, while puffins from Bjørnøya are located between Spitsbergen and a larger, central cluster consisting of populations from Norway, Iceland, and the Faroe Islands (Fig. 1c, Fig. S5a). Puffins from Canada form their own distinct cluster, as do those from the Isle of May, southeast Scotland (Fig. 1c, Fig. S5b). Hierarchical PCA analyses of the cluster comprising the mainland Norwegian, Icelandic and Faroese colonies reveal further fine-scale structure separating Norwegian (Hornøya and Røst) and Faroese/Icelandic colonies (Fig. S5c). Model-based clustering (ngsAdmix) agrees with the results from the PCA (Fig. 1d). The optimal model fit for the entire dataset is either *K* = 2 or *K* = 4 (Fig. S6a), as determined by the method of Evanno et al.^41^. At *K* = 2, ngsAdmix separates Spitsbergen from the other colonies, with Bjørnøya being admixed (following separation along PCA 1), whereas at *K* = 4, ngsAdmix reflects the structure of three additional distinct clusters representing Spitsbergen, Canada, the Isle of May, and a central group with more shared ancestry (Fig. 1d). The shared ancestry of the central group remains present in hierarchical admixture analyses excluding Spitsbergen and Bjørnøya individuals (Figs. S6b, S7). We find no fixed alleles and pairwise F_ST_ values between colonies and genomic clusters are low (<0.01) (Table S4), apart from any comparisons involving the Spitsbergen population, which show substantially higher F_ST_ values (0.03–0.08).

**Fig. 2:**
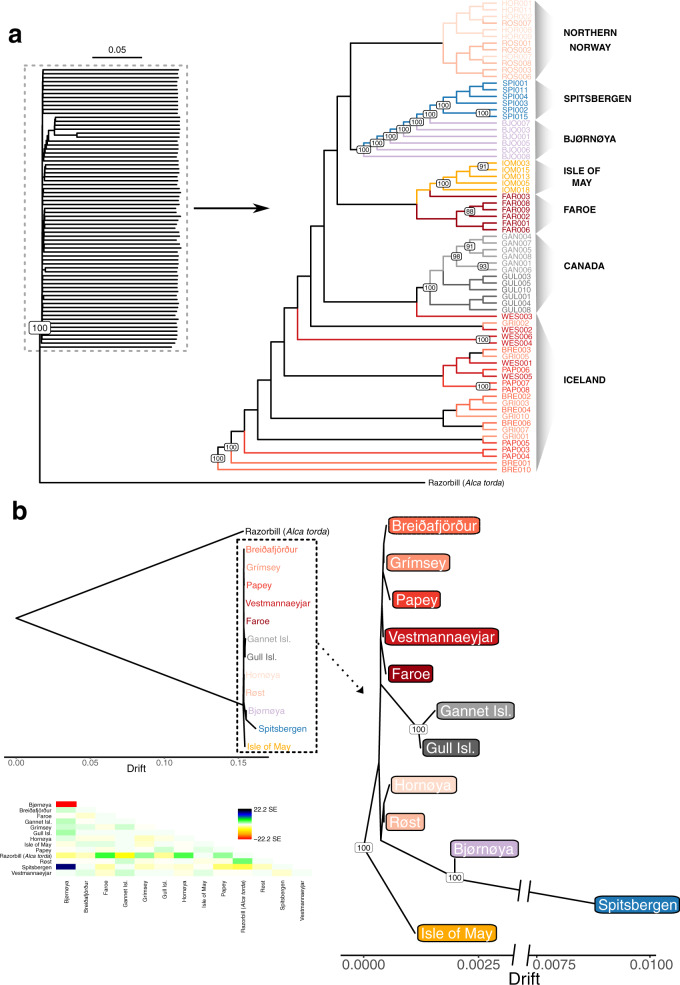
Phylogenetic reconstruction of individual and colony relationships from 71 Atlantic puffin individuals sampled across 12 colonies throughout the species’ breeding range. **a** An individual-based neighbor-joining tree constructed using pairwise p-distances calculated from genotype likelihoods at 1,093,765 polymorphic nuclear sites. Branch lengths and the outgroup were removed for the zoomed-in section to improve visualization. **b** A population-based maximum likelihood Treemix analysis using allele frequencies at the same 1,093,765 polymorphic nuclear sites as in (**a**). Both trees are rooted using the razorbill as an outgroup. The tree in (**b**) is visualized with and without the outgroup. Branch lengths are equivalent to a genetic drift parameter. The heatmap indicates the residual fit of the tree displaying the standard error of the covariance between populations. In (**a**) and (**b**), the color coding of the colonies is consistent with those in Fig. 1 and node labels show bootstrap support >80. The dataset(s) needed to create this Figure can be found at 10.6084/m9.figshare.14743299.v1.

Phylogenetic reconstructions using individual-based Neighbor-Joining (NJ) and maximum likelihood (ML) methods (Fig. 2a, Fig. S8), as well as population-based analyses in Treemix (Fig. 2b), support the distinctiveness of the Spitsbergen, Canada, and the Isle of May puffins with each group forming monophyletic clades with 100% bootstrap support. In contrast, Bjørnøya forms a paraphyletic clade between Spitsbergen and northern Norway (Fig. 2a). The population clusters identified by the PCA and ngsAdmix at smaller spatial scales are also identified in the topologies of the NJ and ML trees, sorting individuals predominantly according to geographical location, although with low bootstrap support (>80) due to large inter-individual variability (Fig. 2a, Fig. S7). Allowing a single migration edge in the Treemix phylogeny identifies recent gene flow from Spitsbergen to Bjørnøya (likelihood = 792.106; Figs. S9, S10a). Adding additional migration edges to the population-based ML tree does not improve the model fit and such edges are therefore not further interpreted (Figs. S9-S11).

### Genetic diversity, heterozygosity, and inbreeding

**Fig. 3:**
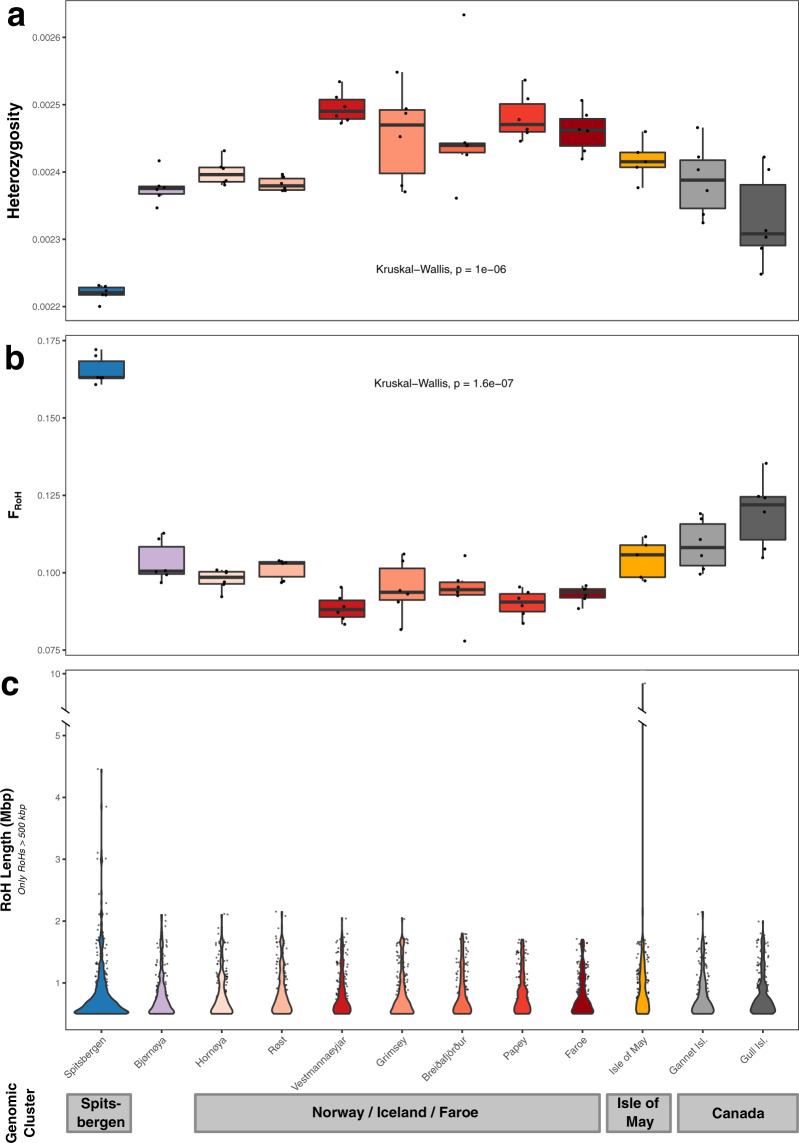
Genome-wide heterozygosity, inbreeding, and Runs-of-Homozygosity (RoH) compared between 12 Atlantic puffin colonies across the species’ breeding range. **a** Estimates of individual genome-wide heterozygosity based on the per-sample one-dimensional Site Frequency Spectrum calculated in ANGSD. **b** Individual inbreeding coefficients, F_RoH_, defined as the fraction of the individual genomes falling into RoHs of a minimum length of 150 kb. RoHs were declared as all regions with at least two subsequent 100 kb windows harboring a heterozygosity below 1.435663 × 10^−3^. **c** RoH length distribution across the 12 colonies only including RoHs longer than 500 kb. A single 9.65 Mbp long RoH on pseudo-chromosome 7 in an Isle of May individual required to introduce a break in the y-axis. In (**a**) and (**b**), black dots indicate individual sample estimates and black lines the median per colony, while in (**c**), black dots represent single RoHs. Statistical significance of differences in heterozygosity and F_RoH_ between populations was assessed with a global Kruskal-Wallis test (*n* = 12). The results of post hoc Dunn tests with Holm corrections are presented in Fig. S12. Error bars show range of values within 1.5 times the interquartile range. Different colonies in all three plots are indicated using the same color code as in Fig. 1. The dataset(s) needed to create this figure can be found at 10.6084/m9.figshare.14743317.v1.

Tajima’s D does not significantly deviate from neutral expectation per colony (Table S3). Nucleotide diversity (*π*) of puffins is significantly different between colonies, with the Spitsbergen population having significantly lower nucleotide diversity than the global median (Wilcoxon Rank Sum test, *U* = 4824, *n*_SPI_ = 25, *n*_Global_ = 300, *P* = 0.017, Table S3). Colonies also differ significantly in levels of heterozygosity (Kruskal–Wallis test, *n* = 12, *P* = 1 × 10^−6^; Fig. 3a) and inbreeding (Kruskal–Wallis test, *n* = 12, *P* = 1 × 10^−7^, Fig. 3b), whereby individual inbreeding (F_RoH_) was approximated based on runs of homozygosity (RoH)^42^. Again, the Spitsbergen colony has significantly lower levels of heterozygosity (0.00220–0.00223) and significantly higher levels of F_RoH_ values (0.161–0.172), compared to the Faroese and Icelandic colonies (Dunn test with Holm correction, *P* < 0.05, *n*_1_ = 6, *n*_2_ = 6). The Faroese and Icelandic colonies contain the highest levels of heterozygosity and lowest F_RoH_ values (Figs. 3a, b, Fig. S12) overall. The remaining colonies display intermediate levels (Fig. 3a, b), although heterozygosity is significantly lower (Fig. 3a, Fig. S12) and inbreeding is significantly higher (Fig. 3b, Fig. S12) on Gull Island and Bjørnøya compared to the Icelandic and Faroese colonies (Dunn test with Holm correction, *P* < 0.05, *n*_1_ = 6, *n*_2_ = 6). Moreover, Spitsbergen harbors the most (an average of 718 per individual) and longest RoHs with eight being ≥2.3 Mbp long (4.21 ± 3.02% of respective chromosome), whereas none of the RoHs in the remaining colonies are >2.15 Mbp long (Fig. 3c). The only exception is a 9.65 Mbp long RoH on pseudo-chromosome 7 (18% of chromosome length) in an Isle of May individual (Fig. 3c).

### Patterns of gene flow and isolation-by-distance (IBD)

**Fig. 4:**
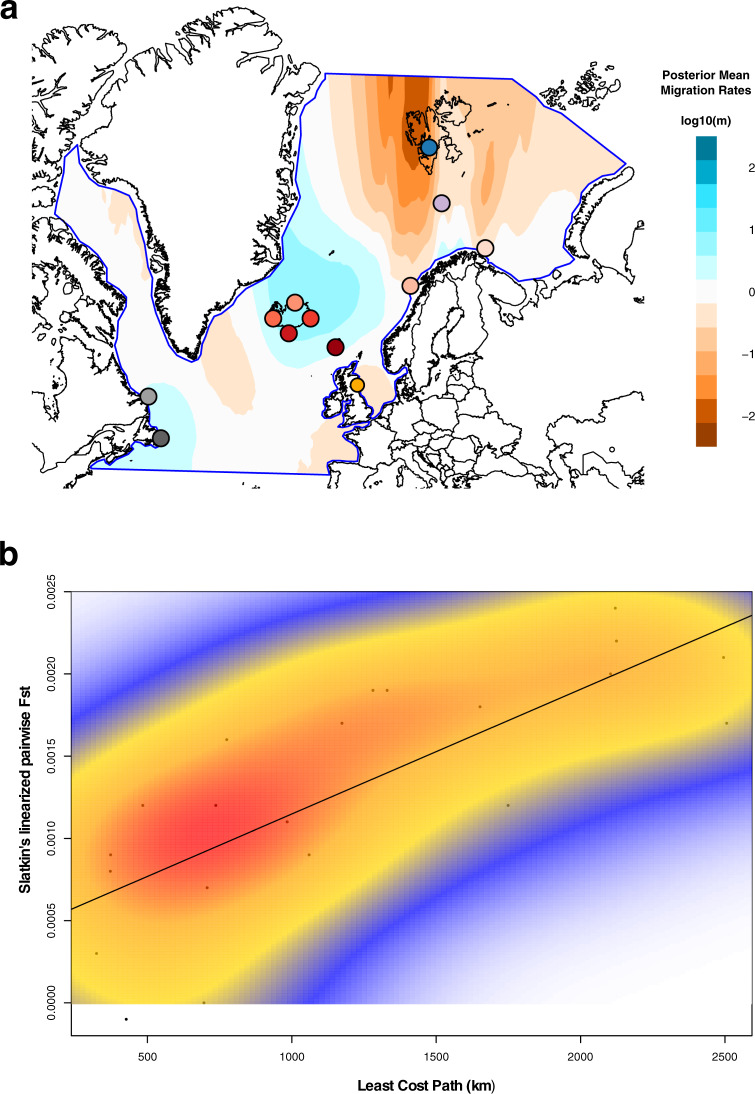
Estimates of continuous long-distance gene flow and isolation by distance (IBD) across the breeding range of the Atlantic puffin estimated from 71 individuals across 12 colonies. **a** Effective migration surfaces inferred by the program EEMS using the average distance between pairs of individuals calculated in ANGSD by sampling the consensus base for each individual at 1,093,765 polymorphic nuclear sites. Darker reds indicate reduced migration across those areas, while darker blues highlight higher migration rates than the global mean. Different colonies are indicated using colors consistent with those in Fig. 1. **b** Correlation between genetic (Slatkin’s linearized F_ST_) and geographic (Least Cost Path—only over water) distance presented after removing the Spitsbergen, Bjørnøya, Isle of May, and Canadian individuals. The diagonal line visualizes the result of the multiple regression on distance matrices (MRM) analysis (slope and y-intercept). The Mantel test between genetic and geographic distance (*R* = 0.775, *P* = 0.012, *n*_Colonies_ = 7) was significant and 60.08% of the variance in Slatkin’s linearized F_ST_ was explained by geographic distance (regression coefficient of linear IBD model = 0.76 × 10^−6^, *P* = 0.006, *n*_Colonies_ = 7). A two-dimensional kernel density estimation (kde2d) highlights dense groups of data points, thus substructure in the genomic landscape pattern. Analyses were conducted and results visualized in R using the *ecodist*, *marmap* and *MASS* packages. The dataset(s) needed to create this figure can be found at 10.6084/m9.figshare.14743323.

We investigated patterns of gene flow and IBD between the colonies using two-dimensional estimated effective migration surface (EEMS) analyses^43^. Levels of gene flow between the Icelandic and Faroese colonies and within the Canadian group is high (3–10× higher than the global average), while intermediate between the Norwegian mainland colonies (around the global average). In contrast, the Spitsbergen colony is split from the remaining colonies by migration rates up to 100× lower than the global average (Fig. 4a, Fig. S13), while additional regions of low gene flow (2–3× lower than the global average) separate the Isle of May, Canadian, and Bjørnøya colonies from the rest (Fig. 4a, Fig. S13). Geographic distance between all puffin colonies is a poor predictor of pairwise genetic distance, driven by high Slatkin’s linearized F_ST_ values between Spitsbergen and the other colonies (Tables S5, S6, Fig. S14). Nevertheless, the geographic distance among a subset of puffin colonies is significantly associated with genetic distance as shown by Mantel tests, linear regression model analyses, and distance-based Redundancy Analysis (dbRDA) models (Fig. 4b, Fig. S14, Tables S5, S6). Specifically, by progressively removing the more distant colonies (Spitsbergen, Isle of May, Bjørnøya, Canada), which are characterized by high Slatkin’s linearized F_ST_ values at relatively small geographic distances (Fig. S14), the fit of a linear IBD model is significantly improved and the proportion of variance of genetic dissimilarity explained by geographic distance is more than doubled (Spitsbergen removed: 37.58%; Spitsbergen/Isle of May/Bjørnøya/Gannet Isl. removed: 84.98%) (Fig. 4b, Fig. S14, Table S5). Similarly, the proportion of explained genetic variance by spatial features estimated in global dbRDA models is more than tripled (All colonies = 18.76%, Spitsbergen/Isle of May/Bjørnøya removed = 59.87%) (Table S5). In all optimized dbRDA models, geographic variables (IBD) contribute significantly to the genetic divergence, while the contribution of the mean sea surface temperature (isolation-by-environment, IBE) is minimal. IBE is only once significantly contributing to the observed genetic variance (when Spitsbergen was removed), yet accounts for less than half of the observed genetic variance (11.37%) compared to the geographic distance (28.66%) (Table S6).

### Admixture on Bjørnøya

We specifically tested for patterns of admixture in Bjørnøya. Significantly negative *f*3 statistics (Z score < −3) are found for all unique combinations of the phylogeny (Spitsbergen, *X*; Bjørnøya) (Table S7), indicating an admixed colony on Bjørnøya caused by gene flow between Spitsbergen and the remaining colonies. Similarly, significantly positive D-statistics (Z score > 3) caused by an excess of ABBA sites reveal excessive allele sharing between Spitsbergen and Bjørnøya (Fig. S15a). The close association and gene flow from Spitsbergen to Bjørnøya is further confirmed by D-statistics not being significantly different from 0 for the (((Bjørnøya, Spitsbergen), H3), Razorbill) topology (Fig. S15b).

### Genetic differentiation

We assessed genome-wide patterns of genetic differentiation by calculating pairwise F_ST_ between the four genomic clusters in 50 kb sliding windows. These analyses show that the differentiation between the clusters is driven by increased F_ST_ in windows across the entire genome, including the presence of several smaller regions with elevated F_ST_ (Fig. S16). Several of these elevated F_ST_ regions are present in all pairwise comparisons (Fig. S16), whereas others are specific for certain comparisons, and may be indicative of local adaptation (Fig. S16).

## Discussion

Barriers to gene flow leading to population structure are notoriously difficult to identify and remain largely unknown for most seabirds^15,44^. Using whole-genome analyses, we here provide insights into the genetic structure of the Atlantic puffin. First, we identify four main puffin population clusters consisting of (1) Spitsbergen (High Arctic), (2) Canada, (3) Isle of May, and (4) multiple colonies in Iceland, the Faroe Islands, and Norway. Second, we find that within such clusters, genetic differentiation is driven by IBD. Finally, we find evidence for secondary contact between two clusters. These observations show that a complex set of drivers impacts gene flow over different spatial scales (100–1000s of km) between these clusters and the colonies within. In particular, the interplay between overwintering grounds, philopatry, natal dispersal, geographic distance, and potentially ocean regimes appears to explain the genomic differentiation between puffin colonies^45^.

Mature puffins rarely, if ever, change their colonies, resulting in very high colony fidelity once they start breeding^28^. Immatures, however, have been observed to visit other nearby colonies during the summer and may breed in non-natal colonies^28,46^. Nevertheless, data on natal philopatry remain scarce, but existing evidence shows rates vary greatly (38–92%) between colonies^28,46^. If either breeding or natal philopatry alone drive the puffin population structure, each colony should constitute its own distinct genomic entity and substantial genomic differentiation across the puffin’s entire breeding range would be observed. Yet, philopatry alone cannot explain the presence of the four large-scale population clusters we observe here. Additional factors must therefore promote the distinctiveness of the four clusters. For instance, the Isle of May birds have a largely separate overwintering distribution mainly in the North Sea (Fig. S17)^28,38,47^. Such potential geographical separation during the winter season might limit the likelihood of immatures intermixing between the Isle of May and other colonies. Similarly, distinct overwintering distributions have been found to lead to increased genetic diversification in other philopatric seabird species^15,44,45^, such as the thick-billed murre (*Uria lomvia*)^21^ and black‐browed albatross (*Thalassarche melanophris*)^48^. The presence of a Canadian cluster can also be largely explained by their winter distribution around Newfoundland^47,49^. There is, however, some fragmentary overlap in the overwintering distribution of the Canadian and Icelandic colonies off southwestern Greenland^47,49^, suggesting that barriers to dispersal of immatures and gene flow in the western Atlantic may be further enforced by the large geographic distance. In contrast, the winter distribution from the colonies in Iceland, Norway, and the Faroe Islands overlaps off the coast of southern Greenland (Fig. S17)^47^. This shared overwintering area, combined with the tendency to return to the natal colony and immature visits to nearby (up to 100 s km) colonies during the summer, appears to drive a pattern of IBD among colonies (Fig. 3b). Indeed, IBD has previously been recognized as an important driver of genomic structure in seabirds, for instance in the little auk (*Alle alle*)^50^ and band-rumped storm-petrel (*Oceanodroma castro*)^51^. While these illustrated mechanisms provide reasonable explanations for the observed dispersal barriers and population structure based on our current knowledge, validation requires additional evidence, specifically on the winter distribution of immature puffins and natal dispersal rates across colonies covering the entirety of the puffin’s breeding range.

High Arctic puffins from Spitsbergen are genetically the most divergent group within our dataset harboring the highest genome-wide differentiation. They are also characterized by significantly lower levels of genetic diversity, greater inbreeding coefficients, and longer and more abundant RoHs compared to other colonies. These observations may either result from a historical bottleneck followed by isolation (e.g., founder effect), local adaptation to their extreme environment, or generally lower effective population sizes. Population abundance estimates of <10,000 breeding pairs on Spitsbergen compared to 500,000 in the West Atlantic, two million on Iceland and more than two million in the boreal East Atlantic potentially indicate a lower effective population size^28^. The High Arctic puffins exclusively inhabit harsh, cold-current environments year-round, as they likely stay in an area bounded by the East Greenland ice edge, a latitudinal border at 70° N, and the front between the Barents and Greenland Sea during winter (Fig. S17). They are also substantially larger than birds from lower latitudes^28,33,34^, following Bergmann’s^52^ or James’s^53^ rule, as has been observed in other seabirds^54,55^. This matches the clinal size variation of puffins that closely tracks sea temperatures in their breeding areas^56^. Despite these distinctions, we find that the relatively small population of puffins on Bjørnøya (<1000 pairs^28^), midway between Spitsbergen and mainland Norway, represents an area of secondary contact between the puffins from the High Arctic and other puffin colonies. Based on D- and the *f*3-statistics, the most likely southern sources are Iceland, the Faroe Islands, Norway, or a combination thereof. Thus, the barriers to gene flow that keep the Spitsbergen colonies distinct do not prevent the formation of a hybrid colony where individuals from the High Arctic and the cluster composed of mainland Norwegian, Icelandic and Faroese colonies meet.

The distinct population structure in the nuclear data is not observed in the mitochondrial genomes, which reveal an abundance of rare alleles and lack of significant population differentiation. The mitogenomic variation suggests that puffins experienced a recent population expansion, possibly out of a refugium after the Last Glacial Maximum. Indeed, it has been shown that mitogenomic variation in seabirds is dominated by historical factors rather than representing contemporary gene flow^44^, and a lack of mitogenomic population structure has been observed in many marine birds with high philopatry^50,57,58^. In contrast to the mitogenomes, the structure in the nuclear data therefore likely originated after the last glacial period and reflects the influence of relatively recent barriers to gene flow in a context of historical demography^15,44^. Such results are relevant for understanding the “seabird paradox”, which contrasts the life-history traits of high philopatry and restricted dispersal in otherwise highly mobile species^59^.

Our results have major implications for the conservation management of the Atlantic puffin. The genetic structure we identify in puffins disagrees with the suggestion of three subspecies (*F. a. naumanni, F. a. arctica, F. a. grabae*)^36^. Although the genetically distinct Spitsbergen cluster coincides with the classification of morphologically large puffins in the High Arctic (*F. a. naumanni*)^28^, we observe gene flow from Spitsbergen into Bjørnøya, which has been considered *F. a. arctica*^28^. Furthermore, the geographic divide between *F. a. grabae* and *F. a. arctica* lies farther south than previously thought, with the Faroese puffins being genetically closer to *F. a. arctica* than to *F. a. grabae*. Nonetheless, *F. a. grabae* is currently represented by a single colony (Isle of May) in our study and the geographical extent of this genomic cluster needs to be refined by additional sampling, particularly in the western UK, Ireland, and France. Finally, puffins from the Western Atlantic region (e.g., colonies in Canada) form their own distinct genetic cluster that is not recognized within the current classification. Our results do not only warrant a revision of Salomonsen’s taxonomic classification of three subspecies^36^, but also highlight the need to acknowledge the four identified clusters as distinct units within the conservation management of puffins^11,13,14^. Although puffin colonies within clusters are not genetically distinct entities, ecological independence illustrated by contrasting population dynamics across relatively small spatial scales (e.g., western Norway^31^) suggests that higher resolution local management units based on ecological differences should be considered. Nonetheless, the genetically distinct clusters at the outer edges of the puffin’s distribution with putative local adaptations that will not be easily replenished indicate that conservation of these distinct clusters must be a first priority. Finally, our sampling does not cover several outskirts of the puffin’s distribution, such as the U.S., northern Canada, Greenland, Ireland, western UK, France or Russia, and we may therefore still underestimate the true biological and genetic complexity of this species.

In conclusion, our study shows that a complex interplay of barriers to gene flow drives a previously unrecognized population diversification in the iconic Atlantic puffin. So far, much of seabird population genetics research has been based on mitochondrial and microsatellite data^15,44^, which have limited power to characterize contemporary factors that determine population structure and gene flow^20,60^. High-resolution nuclear data are therefore essential to help define evolutionary significant population units, disentangle convoluted ecological relationships, and are particularly important for seabird conservation, which aims to preserve genetic diversity considering profound global population declines^7,8^, and the threat of global warming, which negatively impacts ecosystems worldwide^61^.

## Methods

### Ethical statement

Feather and blood samples of puffins included in this study were collected and handled under the following permits.

1. Gåsøyane, Røst, Hornøya, Bjørnøya (Norway)—FOTS ID #15602 and #15603 from the Norwegian Food Safety Authority for SEATRACK and SEAPOP; Permit 2018/607 from Miljødirektoratet (Norwegian Environment Agency), dated 4 May 2018.
2. Gannet and Gull Island (Canada)—Canadian Wildlife Service Migratory Bird Banding Permit 10559 G, approved Animal Use Protocol (AUP) by Eastern Wildlife Animal Care Committee (17GR01, 18GR01), Newfoundland and Labrador Wilderness and Ecological Reserves Permit—Scientific Research (DOC/2017/02003), Canadian Wildlife Service Scientific Permit ST2785 (to M.L.M.), Canadian Wildlife Service Banding Permit 10694, and Acadia University Animal Care Committee Permits ACC 02-15 and 06-15 (to M.L.M.).
3. Isle of May (Scotland)—Scottish Natural Heritage licence 2014/MON/RP/156 and Ringing Permit A400 (to MPH).
4. Vestmannaeyjar, Papey, Breiðafjörður, Grímsey (Iceland)—Icelandic puffins were legally hunted during the hunting period of 1 July–15 August.
5. Faroe—Feathers came from predated birds collected in the field after the predator was finished with them.

### Draft reference genome assembly

A de novo Atlantic puffin draft genome was generated from the blood of a female Atlantic puffin. Read data were sequenced on three Illumina HiSeqX lanes using the 10x Genomics Chromium technology and assembled with the Supernova assembler (v2.1.1)^62^ after subsampling to 0.8 billion and 1 billion reads to maximize performance and remain within the computational capacity of the assembler. We refined the two assemblies through several steps, including merging of ‘haplotigs’, removal of contaminant sequences, misassembly correction, re-scaffolding using mapping coverage and linkage information, and gap filling (Supplementary Data 1a). The most complete and continuous 800 M and 1000 M assemblies together with the 3rd best assembly overall were selected for a second round of refinement (Supplementary Data 1b) resulting in a total of 72 draft assemblies. Of these, we kept the four most complete and continuous assemblies for additional gap filling and polishing, after which the most complete draft genome was selected for downstream analyses (Supplementary Data 1c). The puffin mitogenome was confidently identified by blasting (blastn) all scaffolds shorter than 25 kb against a custom-built database of 135 published mitogenomes of the order ‘Charadriiformes’ and annotated with the MITOS web server^63^ (Fig. S1). The remaining nuclear scaffolds were ordered and concatenated into “pseudo-chromosomes” by mapping them to the razorbill genome (*Alca torda*—NCBI: bAlcTor1 primary, GCA_008658365.1) and applying 200 N’s as padding between each scaffold. We combined unmapped scaffolds into an “unplaced” pseudo-chromosome. We assessed the order and placement of scaffolds by investigating synteny in coverage and length between the puffin and razorbill chromosomes (Table S1). Details on the draft reference genome assembly and refinement can be found in the Supplementary File.

### DNA extraction and sequencing

Samples from a total of 72 puffins collected across 12 breeding colonies (Fig. 1a) were made available for the present study by SEAPOP (http://www.seapop.no/en), SEATRACK (http://www.seapop.no/en/seatrack) and ARCTOX (http://www.arctox.cnrs.fr/en/home—Canadian colonies). These samples had been collected between 2012 and 2018 and consisted of blood preserved in EtOH or lysis buffer, or feathers (Supplementary Data 2). We extracted DNA using the DNeasy Blood & Tissue kit (Qiagen) following the manufacturer’s protocol for animal blood or the nail/hair/feathers protocol applying several modifications for improved lysis and DNA yield. Individuals that had no sexing data associated with them were sexed using PCR amplification of specific allosome loci and visualization via gel electrophoresis. Genomic libraries were built by the Norwegian Sequencing Centre and sequenced on an Illumina HiSeq4000. We processed sequencing reads in PALEOMIX v1.2.14^64^ and split the resulting bam files into nuclear and mitochondrial bam files. Additional details on the DNA extraction, sexing, sequencing and mapping are listed in the Supplementary File.

### Mitogenome analyses

Genotypes across the mitochondrial genome were jointly called with GATK v4.1.4^65^ by using the *HaplotypeCaller*, *CombineGVCFs,* and *GenotypeGVCFs* tool. We filtered genotypes according to GATKs Best Practices^66^ and set genotypes with a read depth <3 or a quality <15 as missing. Indels and non-biallelic SNPs were removed and only SNPs present in all individuals were kept for subsequent analyses. The SNP dataset was annotated (Supplementary Data 3) with snpEff^67^ utilizing the annotation of the newly assembled mitogenome of the Atlantic puffin and converted into a mitogenome sequence alignment. To serve as an outgroup, we appended four other species of the family Alcidae, i.e., the Razorbill (*Alca torda*, NCBI: CM018102.1), the Crested Auklet (*Aethia cristatella*, NCBI: NC_045517.1), the Ancient Murrelet (*Synthliboramphus antiquus*, NCBI: NC_007978.1) and the Japanese Murrelet (*Synthliboramphus wumizusume*, NCBI: NC_029328.1), to the alignment. To construct a maximum-likelihood phylogenetic tree, we split the alignment into seven partitions, i.e., one partition for a concatenated alignment of each of the three codon positions of the protein-coding genes, one partition for the concatenated alignment of the rRNA regions, one partition for the concatenated alignment of the tRNAs, one partition for the alignment of the control region, and one partition for the concatenated alignment of the “intergenic” regions. The best-fitting evolutionary model for each partition was found by *ModelFinder*^68^ and the tree was built with IQTree v1.6.12^69^ using 1000 ultrafast bootstrap replicates. We used the resulting tree to draw a haplotype genealogy graph with Fitchi^70^. Using Arlequin v.3.5^71^, we calculated haplotype (h), nucleotide diversity (π), and Tajima’s D^72^ for each colony, for each genomic cluster defined by the nuclear analysis, and globally. In addition, an Ewens–Watterson test^73^, Chakraborty’s test of population amalgamation^74^, and Fu’s F_s_ test^75^ were conducted for each of those groups. To further identify population differentiation, the proportion of sequence variation (Φ_ST_) was estimated for all pairs of populations and genomic clusters. Hierarchical AMOVA tests subsequently determined the significance of a priori subdivisions into colonies and genomic clusters. Calculation of Φ_ST_ and AMOVA tests were also conducted in Arlequin. Additional details on the mitochondrial analyses are given in the Supplementary File.

### Nuclear genome clustering and phylogenetic analyses

The majority of population genomic analyses were based on nuclear genotype likelihoods as implemented in ANGSD v.0.931^76^. After assessing the quality of the mapped sequencing data in an ANGSD pre-run, we removed an individual from the Isle of May from the dataset. Genotype likelihoods for nuclear SNPs covered in all individuals were calculated and filtered in ANGSD. Accounting for linkage disequilibrium, we further pruned the dataset by only selecting the most central site within blocks of linked sites (*R*^2^ > 0.2) as in Orlando and Librado^77^. Subsequently, all variants located on the Z-pseudo-chromosome and “unplaced scaffolds” were excluded from the analyses yielding a final genotype likelihood panel consisting of 1,093,765 sites. We investigated genomic population structure with a PCA of the genotype likelihood panel using PCAngsd v0.982^78^. Individual ancestry proportions were estimated using a maximum likelihood (ML) approach implemented in ngsAdmix v32^79^, with the number of ancestral populations (K) set from 1 to 10 and conducting 50 replicate runs for each K. The runs were clustered after similarity for each K and ancestry proportions were averaged within the major cluster using Clumpak^80^ with default settings. Additional “hierarchical” PCA and admixture analyses were conducted for genomic sub-cluster(s) using identical methods.

After adding the razorbill genome as an outgroup to the genotype likelihood panel by mapping unpublished, raw 10x Genomics sequencing data used for the assembly of the embargoed razorbill genome to the puffin draft assembly, we built a neighbor-joining (NJ) tree based on pairwise genetic distance matrices (p-distance) and a sample-based ML phylogenetic tree in FastMe v2.1.5^81^ and Treemix v1.13^82^, respectively. For both trees, 100 bootstrap replicates were generated. To infer patterns of population splitting and mixing, we produced population-based ML trees including up to ten migration edges. The optimal number of migrations was selected using a quantitative approach by evaluating the distribution of explained variance, the log likelihoods, the covariance with an increase in migration edges, and by applying the method of Evanno^41^ and several different linear threshold models. The topology for m_0_ and m_BEST_ was evaluated by generating 100 bootstrap replicates. Additional details on the cluster and phylogenetic analyses are given in the Supplementary File.

### Genetic diversity, heterozygosity, and inbreeding

We calculated a set of neutrality tests and population statistics in ANGSD using colony-based one-dimensional (1D) folded site frequency spectra (SFS). For each population, genomic cluster, and globally, Tajima’s D and nucleotide diversity (π) were computed utilizing the per-site θ estimates. Individual genome-wide heterozygosity was calculated in ANGSD using individual, folded, 1D SFS. We calculated heterozygosity by dividing the number of polymorphic sites by the number of total sites present in the SFS.

The proportion of RoH within each puffin genome was computed by calculating local estimates of heterozygosity in 100 kb sliding windows (50 kb slide) following the approach in Sánchez-Barreiro et al.^42^. We defined the 10% quantile of the average local heterozygosity across all samples as the cutoff for a “low heterozygosity region” (Fig. S18). RoHs were declared as all regions with at least two subsequent windows of low heterozygosity (below cutoff) and their final length was calculated as described in Sánchez-Barreiro et al.^42^. We calculated an individual inbreeding coefficient based on the RoH, F_RoH_, as in Sánchez-Barreiro et al.^42^ by computing the fraction of the entire genome falling into RoHs, with the entire genome being the total length of windows scanned. Additional details on these analyses can be found in the Supplementary File.

### Patterns of gene flow and admixture

Assessing potential patterns of IBD within the breeding range of the puffin, the program EEMS^43^ was used to model the association between genetic and geographic data by visualizing the existing population structure and highlighting regions of higher-than-average and lower-than-average historic gene flow. We calculated a pairwise genetic distance matrix in ANGSD by sampling the consensus base (*-doIBS 2 -makeMatrix 1*) at the sites included in the genotype likelihood set (see *Nuclear cluster and phylogenetic analyses*) for each sample. The matrix was fed into 10 independent runs of EEMS, each consisting of one MCMC chain of six million iterations with a two million iteration burn-in, 9999 thinning iterations, and 1000 underlying demes.

Supplementing the results of the EEMS analysis, we conducted a traditional IBD analysis by determining geographical and genetic distances between the 12 colonies and assessing the significance of the correlation between the two distance matrices with a Mantel test^83^ and a multiple regression on distance matrix (MRM)^84^ analysis. F_ST_ was used as a proxy for genetic distance and computed for each population pair in ANGSD by applying two-dimensional (2D), folded SFS. We converted pairwise F_ST_ values to Slatkin’s linearized F_ST_^85^. Least Cost Path distances (paths over water only) between colony coordinates (latitude/longitude) were calculated using the R package *marmap*^86^ and used as geographic distances. We performed the Mantel test (999 permutations) and MRM analysis with the R package *ecodist*^87^. All analyses for IBD were re-run on subsets of colonies by progressively removing the colony from the geographic and genetic distance matrices, whose removal led to the highest increase in the proportion of variance in genetic distance explained by geographic distance in the resulting regression model (Spitsbergen, Isle of May, Bjørnøya and Gannet Isl.).

A distance-based Redundancy Analysis (dbRDA)^88^ was conducted to corroborate the results of the MRM analyses and Mantel tests and to estimate the relative contribution of IBD and IBE to the observed Atlantic puffin population structure. The dbRDA was run between the genetic distance matrix versus geographic and environmental parameters^88^. A global dbRDA was performed with all geographic and environmental variables, and for statistically significant global dbRDA models, the most significant variables (geographic or environmental) were selected via a stepwise regression^89^. Those served as input for a reduced dbRDA to calculate the marginal effect of each variable and for a partial dbRDA with variance partitioning to estimate the separate effects of IBD and IBE. Similar to the MRM analyses and Mantel tests, these analyses were repeated on subsets of colonies by progressively removing the colony from the geographic, environmental, and genetic distance matrices, whose removal led to the highest increase in variance explained in the resulting global dbRDA model. Methods and R code for the dbRDA were found at https://github.com/laurabenestan/db-RDA-and-db-MEM^90^.

Additional assessments of gene flow and admixture were conducted by calculating *f*3-statistics and multi-population D-statistics (aka ABBA BABA test)^91^. We calculated *f*3-statistics in Treemix for each unique combination of ((A,B),C)) of the 12 puffin populations. D-statistics were calculated in ANGSD (-doAbbababa2) for each combination of ((A,B),C),Outgroup) using the 12 puffin colonies. The outgroup was generated in ANGSD using the 10xGenomics sequencing data of the razorbill mapped to the puffin reference genome (see *Nuclear cluster and phylogenetic analyses)*.

Evaluating genome-wide patterns of genetic differentiation, pairwise F_ST_ values between the Norway/Iceland/Faroe cluster and the Spitsbergen, Isle of May, Canada colonies (three comparisons) were calculated in sliding windows of 50 kb with 12.5 kb steps across the 25 pseudo-chromsomes by applying 2D, folded SFS. The window size of 50 kb was chosen for sliding window analyses because LD decays to ca. 10% (*R* < 0.025) within this distance (Fig. S19). Additional details on the IBD, admixture, and sliding-window analyses are given in the Supplementary File.

### Statistics and reproducibility

The research sample included 72 adult Atlantic puffins (*Fratercula arctica*) across 12 colonies located in Svalbard, northern mainland Norway, Iceland, the Faroe Islands, Scotland, and Canada. The sample included six individuals per colony (12 colonies), including an equal sex ratio (3 males and 3 females per colony). All statistical tests were conducted using publicly available programs and packages as described in the methodological sections above. Reproducibility can be accomplished by following the sample collection and laboratory methods outlined above and by following the author’s GitHub (https://github.com/OKersten/PuffPopGen) using the specified parameters mentioned in in the code and methodological sections above.

### Reporting summary

Further information on research design is available in the Nature Research Reporting Summary linked to this article.

## Supplementary information

Supplementary Information

Reporting Summary

Supplementary Data 1

Supplementary Data 2

Supplementary Data 3

Description of Additional Supplementary Files

## Data Availability

Raw read data analyzed in the current study have been deposited in the European Nucleotide Archive (ENA, www.ebi.ac.uk/ena) under study accession number PRJEB40631 (see Supplementary Data 2 for individual sample accession numbers). Nuclear and mitochondrial scaffolds (GCA_905066775.1, CAJHIB010000001-CAJHIB010013329), as well as pseudo-chromosomes (GCA_905066775.2, CAJHIB020000001-CAJHIB020000027), have been uploaded to ENA (Project PRJEB40926, Sample SAMEA7482542).

## Code availability

Full code used for the population genomic analyses is available on the first author’s GitHub (https://github.com/OKersten/PuffPopGen) and on Zenodo under the 10.5281/zenodo.4899574^92^. This includes versions of any software used, if relevant, and any specific variables or parameters used to generate, test, and process the dataset of this study.
