# Supplementary material for: Complex population structure of the Atlantic puffin revealed by whole genome analyses
Source: Commun Biol. 2021 Jul 29;4:922. doi: 10.1038/s42003-021-02415-4 (PMC8322098; doi:10.1038/s42003-021-02415-4)
Supplement: Supplementary file 1 — Supplementary Information [file 42003_2021_2415_MOESM1_ESM.pdf]

Complex population structure of the Atlantic puffin revealed by whole genome analyses

Oliver Kersten[1]*, Bastiaan Star[1], Deborah M. Leigh[2], Tycho Anker-Nilssen[3], Hallvard Strøm[4], Jóhannis Danielsen[5], Sébastien Descamps[4], Kjell E. Erikstad[6,7], Michelle G. Fitzsimmons[8], Jérôme Fort[9], Erpur S. Hansen[10], Mike P. Harris[11], Martin Irestedt[12], Oddmund Kleven[3], Mark L. Mallory[13], Kjetill S. Jakobsen[1], Sanne Boessenkool[1]*

[1]Centre for Ecological and Evolutionary Synthesis (CEES), Department of Biosciences, University of Oslo, Blindernveien 31, 0371 Oslo, Norway
[2]WSL Swiss Federal Research Institute, Zürcherstrasse 111, 8903 Birmensdorf, Switzerland
[3]Norwegian Institute for Nature Research (NINA), Høgskoleringen 9, NO-7034 Trondheim, Norway
[4]Norwegian Polar Institute, Fram Centre, Postbox 6606, Langnes, 9296 Tromsø, Norway
[5]Faroe Marine Research Institute (FAMRI), Nóatún 1, FO-100 Tórshavn, Faroe Islands
[6]Norwegian Institute for Nature Research (NINA), Fram Centre, Postbox 6606, Langnes, 9296 Tromsø, Norway
[7]Centre for Biodiversity Dynamics (CBD), Norwegian University of Science and Technology (NTNU), Trondheim, Norway
[8]Environment and Climate Change Canada, Newfoundland and Labrador, Canada
[9]Littoral, Environment et Sociétés (LIENSs), UMR 7266 CNRS – La Rochelle Université, 17000 La Rochelle, France
[10]South Iceland Nature Research Centre, Ægisgata 2, 900 Vestmannaeyjar, Iceland
[11]UK Centre for Ecology & Hydrology, Penicuik, Midlothian, EH26 0QB, UK
[12]Department for Bioinformatics and Genetics, Swedish Museum of Natural History, Box 50007, 104 05 Stockholm, Sweden
[13]Department of Biology, Acadia University, Wolfville, Nova Scotia, Canada, B4P 2R6

**Email: oliver.kersten@ibv.uio.no, sanne.boessenkool@ibv.uio.no*

SUPPLEMENTARY FILE

1. Methods - Draft Reference Genome Assembly **3**
1.1 DNA Extraction and Sequencing 3
1.2 Initial Assembly 3
1.3 Assembly Refinement 3
1.4 Mitochondrial Genome Parsing and Annotation 4
1.5 Nuclear Chromosome Ordering 4
2. Methods - Population Genomic Analyses **5**
2.1 Sampling and DNA Extraction 5
2.2 Sexing 5
2.3 Sequencing and Data Processing 6
2.4 Mitochondrial Analysis 6
2.5 Nuclear Analysis 6
2.5.1 Genotype Likelihoods 6
2.5.2 Population Structure 8
2.5.3 Phylogenetic Analyses 7
2.5.4 Tajima's D and Nucleotide Diversity 8
2.5.5 Heterozygosity, Runs-of-Homozygosity, and Inbreeding 9
2.5.6 Gene flow and Isolation by Distance 9
2.5.7 D- and f3-statistic 11
2.5.8 Genome-wide patterns of genetic differentiation 11
3. Results - Mitochondrial Population Structure **12**
4. Supplementary Tables **13**
5. Supplementary Figures **20**
6. References **39**

1. Methods - Draft Reference Genome Assembly

1.1 DNA Extraction and Sequencing

For the construction of a *de novo* Atlantic puffin genome assembly, a fresh blood sample was collected on 13 June 2018 from a female Atlantic puffin (ring no.: MA28445, Zool. Museum Oslo) breeding on Hernyken (67°25’33’’N 11°52’50’’E), Røst, northern Norway, each year since 2014 (still present 2020). High molecular weight (HMW) DNA was extracted from 15 µl of blood using the Kingfisher Cell and Tissue DNA Kit following the manufacturer's protocol. This was used to prepare a single 10x Genomics Chromium technology library, which was sequenced on three Illumina HiSeqX lanes (150 bp insert size) at the SciLifeLab in Stockholm, Sweden. Each lane generated ~600-760 million paired-end reads for a total of ~ two billion reads.

1.2 Initial Assembly

To maximize performance and remain within the computational capacity of the assembler, two draft genomes were assembled with the Supernova assembler (v2.1.1, 10x Genomics[1]) after subsampling to 0.8 billion and 1 billion reads, respectively. The initial 0.8 billion Supernova assembly, hereinafter referred to as the 800M assembly, was 1.324 Gbp long and consisted of 22,635 scaffolds with a genomic scaffold N50 length of 0.758 Mbp. The initial 1 billion assembly, hereinafter referred to as the 1000M assembly, was 1.342 Gbp long and consisted of 23,650 scaffolds with a genomic scaffold N50 length of 0.711 Mbp. Subsequently, improvements to the two initial assemblies were made through several refinement steps using the reads from each HiSeqX lane separately, as well as all of their possible combinations (Supplementary Data 1a).

1.3 Assembly Refinement

The refinement of the assembly consisted of several downstream steps. Following recommendations of the SciLifeLab Stockholm (pers. comm.) and the BC Cancer Canada's Michael Smith Genome Sciences Centre (see https://warrenlr.github.io/papers/DeNovoAssemblyBTL.pdf for an overview) as performed in previous genome assemblies[2–5], measures included merging of ‘haplotigs’, removal of contaminant sequences, misassembly correction, re-scaffolding using mapping coverage and linkage information, and gap filling (Supplementary Data 1a).

First, using ’purge_haplotigs’[6], pairs of syntenic contigs that were falsely assembled as separate contigs due to a high degree of heterozygosity were identified and one of them removed based on read depth and alignment score[6]. Concurrently, contigs with an exceptional high or low coverage of mapped reads (part of the ‘purge_haplotigs’ output) were blasted (BLASTn) against the NCBI nr_v5 database and “non-Eukaryote” and RNA contigs were removed. Subsequently, misassemblies in the genome were identified and corrected using Tigmint[7] with default parameters (Supplementary Data 1a). After inspection of the barcode multiplicities (# of reads for each barcode vs. # of different barcodes) within the sequencing data of each lane and their combinations to determine the -m parameter, scaffolding was performed with ARKS[8] and further improved with LINKS v1.8.6[9] using default settings (Supplementary Data 1a). Completeness and continuity of the assemblies were assessed with BUSCO v3[10] using the avian set of the OrthoDB v9 database (4,915 gene groups) and with QUAST v4.6[11].

The most complete and continuous 800M and 1000M assembly, as well as the 3rd best assembly overall, were selected for further refinement (Supplementary Data 1b). Gaps were filled with Sealer[12] applying various values of -k as recommended by the developers, followed by a polishing step using ntCard/ntHits/ntEdit[13] using settings suggested by the authors for this type of data (Supplementary Data 1b). Resulting contigs that were flagged as "non-eukaryotic" by both Kraken2[14] and Blobtools[15], or flagged as "non-eukaryotic" by one and as "unclassified" by the other, were removed to improve the signal to noise ratio for subsequent refinement steps.

The three assemblies were further refined by running a second round of the ARKS pipeline. Following the benchmarking procedure in Coombe et al.[8], Tigmint-ARKS-LINKS was run for all 24 combinations of -k (30, 40, 60, 80, 100, 120) and -a (0.3, 0.5, 0.7, and 0.9), while keeping all other parameters the same as before, except for -d (1000, 2500, 5000, 7500, 10000) and -t (10, 5, 2), as recommended by the developers and applied in previous research[2–5] (Supplementary Data 1b). This resulted in a total of 72 draft assemblies. Assessing the assemblies using BUSCO v3 with the avian set of the OrthoDB v9 database and QUAST v4.6, the following four assemblies were kept for gap filling and polishing: The assembly with 1) the highest number of complete genes, 2) the largest maximum scaffold size, 3) the largest N50 and 4) the fewest number of contigs. Applying the same settings as previously, gaps were filled again with Sealer followed by a polishing step using ntCard/ntHits/ntEdit and continuity and completeness were determined with BUSCO and QUAST (Supplementary Data 1c).

1.4 Mitochondrial Genome Parsing and Annotation

To extract the mitochondrial genome/scaffold from the assembly, all scaffolds shorter than 25 kb were blasted (blastn) against a custom-built database of 135 published mitogenomes of the order 'Charadriiformes'. The resulting significant alignment of a single 17 kb scaffold to a large number of mitogenomes in the reference database was visually inspected in Jalview v2.11.1[16] and the scaffold was confidently identified as the puffin mitogenome.

In this mitogenome, a 40+ bp poly-C region prior to a gap (poly-N) region was hard-masked to prevent erroneous mappings. The mitogenome start was shifted to match the start of most other published Charadriiformes mitogenomes and reverse complemented for the right strand orientation using SeqKit v0.12.0[17].

Annotation was performed with the MITOS web server[18] with the protein prediction method of Al Arab[19]. The annotation was manually inspected and coordinates corrected (extended/shrunk) to match the known amino acid structures of mitochondrial genes (Table S2). The tRNA secondary structure was visualized and checked with tRNAscan-SE[20]. Finally, the circular genome and annotation were visualized in shinyCircos[21](Figure S1).

1.5 Nuclear Chromosome Ordering

Nuclear scaffolds were ordered into "pseudo-chromosomes" using the razorbill genome (*Alca torda* - NCBI: bAlcTor1 primary, GCA_008658365.1), which has previously been assembled by the Vertebrate Genome Project (VGP) and is currently the only available chromosome-level assembly in the Alcidae family. All 15,328 nuclear puffin scaffolds were mapped to the razorbill genome using minimap2 v2.17[22]. Scaffolds were assigned to the razorbill chromosome with the largest number of respective hits/alignments, ordered along the chromosomes according to their first alignment position and concatenated into pseudo-chromosomes using 200 N's as padding

between each scaffold. Scaffolds that didn't align to the razorbill chromosomes were combined into an "unplaced" pseudo-chromosome using 200 N's as padding. Finally, order and placement of scaffolds was assessed by investigating synteny in coverage and length between the puffin and razorbill chromosomes (Table S1). The size of the puffin pseudo-chromosomes were of similar size as the respective razorbill chromosome counterparts (Table S1). The only exception was the Z pseudo-chromosome, which was likely a merged ZW chromosome, as the puffin was a female and the razorbill a male.

2. Methods - Population Genomic Analyses

2.1 Sampling and DNA Extraction

Samples from a total of 72 puffins collected across 12 breeding colonies were made available for the present study by SEAPOP (http://www.seapop.no/en), SEATRACK (http://www.seapop.no/en/seatrack/) and ARCTOX (http://www.arctox.cnrs.fr/en/home - Canadian colonies). These samples had been collected between 2012-2018 and consisted of blood preserved in EtOH or lysis buffer, or feathers (Figure 1a, Supplementary Data 2).

DNA from blood samples was extracted using the DNeasy Blood & Tissue kit (Qiagen) following the manufacturer's protocol for animal blood, but doubling the amount of proteinase K for improved lysis. Blood preserved in ethanol was de-coagulated prior to the extraction by thorough vortexing and the addition of 15 µl of 0.5M EDTA to 15 µl of whole blood. DNA was eluted in 2 x 200 µl preheated EB buffer (37°C) after a 10 min incubation at room temperature.

For the DNA extraction from feathers, the first 0.5 cm of the feather shaft (calamus), sometimes containing visual droplets of dried blood, were clipped off and used for the extraction. Combining up to six calami per individual, DNA was extracted according to the nail/hair/feathers protocol of the DNeasy Blood & Tissue kit (Qiagen) with the following changes to improve lysis and increase DNA yield (inspired by[23]): the amount of proteinase K was tripled, the volume of 1M DTT was increased to 50 µl, and samples were incubated at 56°C overnight. Additionally, 70 µg of RNaseA were added to each sample prior to the addition of Buffer AL. Finally, DNA was eluted in 2 x 200 µl preheated EB buffer (37°C) after a 10 min incubation at room temperature.

2.2 Sexing

Individuals that had no sexing data associated with them were sexed using PCR amplification of specific allosome loci and visualization via gel electrophoresis. PCR's were done in a 50 µl reaction volume containing 1x AccuPrime *Pfx* Reaction Mix (Invitrogen), 1U AccuPrime *Pfx* DNA Polymerase (Invitrogen), 0.4 mg/ml BSA (New England Biolabs), 0.3 µM of each of the forward (P8) and reverse (M5) primer published by Griffiths et al.[24] and Bantock et al.[25], and 5µl of template. The thermal profile included an initiation step at 95°C for 5 min, followed by 35 (blood extracts) to 40 (feather extracts) cycles of 30 s at 95°C, 30 s at 50°C and 30 s at 68°C, and a final extension step of 68°C for 5 min. Gel products were visualized on a 3 % agarose gel and females were identified as having two bands (ZW), while males only showed one band (ZZ).

2.3 Sequencing and Data Processing

Genomic libraries were built by the Norwegian Sequencing Centre using a TruSeq DNA Nano preparation kit (Illumina) applying DNA shearing to an approximate insert size of 350 bp, and subsequently sequenced on an Illumina HiSeq4000. Each library was either pooled with 31 other samples and sequenced across four lanes, or pooled with 15 other samples and sequenced across two lanes (Canadian samples).

Sequencing reads were processed in PALEOMIX v1.2.14[26]. Specifically, after removing adapters from forward and reverse reads with AdapterRemoval v2.3.1[27] (*--mm3 --minlength25 --collapse yes --trimns yes --trimqualities yes*), reads were mapped to the Atlantic puffin draft assembly using BWA *mem* v0.7.17[28]. Reads that aligned with a quality score (MapQ) of ≥ 25 were kept for duplicate removal with PicardTools v2.18.27[29] and indel realignment using GATKs *IndelRealigner*[30]. Finally, bam files were split into nuclear and mitochondrial bam files using SAMtools v1.9[31].

2.4 Mitochondrial Analysis

Genotypes were jointly called with GATK v4.1.4[30] by using the *HaplotypeCaller* (*--ploidy 1 --ERC GVCF*), *CombineGVCFs* and *GenotypeGVCFs* tool. Genotypes were filtered with BCFtools v1.9[31] by applying *"--SnpGap 10 -e 'QD < 2.0 || MQ < 40 || FS > 60.0 || SOR > 3 || MQRankSum < -12.5 || ReadPosRankSum < -8.0'"* according to GATKs Best Practices[32] and genotypes with a read depth less than 3 or a quality less than 15 were set as missing. Indels and non-biallelic SNPs were removed and only SNPs present in all individuals were kept for subsequent analyses.

The final SNP dataset was annotated with snpEff[33] utilizing the annotation of the newly assembled mitogenome of the Atlantic puffin (see above) and converted into a mitogenome sequence alignment with BCFtools v1.9 (*consensus -H 1 -M N*). To serve as an outgroup, four other species of the family Alcidae, i.e. the Razorbill (*Alca torda*, NCBI: CM018102.1), the Crested Auklet (*Aethia cristatella*, NCBI: NC_045517.1), the Ancient Murrelet (*Synthliboramphus antiquus*, NCBI: NC_007978.1) and the Japanese Murrelet (*Synthliboramphus wumizusume*, NCBI: NC_029328.1), were appended to the alignment using Muscle v3.8.31[34] (*-profile* + *-refine*) and BCFtools v1.9 (*merge --missing-to-ref*).

To construct a maximum-likelihood phylogenetic tree, the alignment was split into seven partitions, i.e. one partition for a concatenated alignment of each of the three codon positions of the protein coding genes, one partition for the concatenated alignment of the rRNA regions, one partition for the concatenated alignment of the tRNAs, one partition for the alignment of the control region, and one partition for the concatenated alignment of the "intergenic" regions. The best-fitting evolutionary model for each partition was found by *ModelFinder*[35] followed by a greedy strategy[36] that starts with the full partition model and subsequently merges two partitions until the model fit does not increase any further, thereby preventing overparameterization. All partitions were set to share the same set of branch lengths, but were allowed to have their own evolutionary rate[37]. The tree was built with IQTree v1.6.12 using 1000 ultrafast bootstrap replicates by resampling partitions and then sites within resampled partitions[38], and each bootstrap tree was optimized using a hill-climbing nearest neighbor interchange (NNI) search based directly on the corresponding bootstrap alignment. The resulting tree was used to draw a haplotype genealogy graph with Fitchi[39].

Using Arlequin v.3.5[40], haplotype (h), nucleotide diversity (π) and Tajima's D[41] were calculated for each colony, for each genomic cluster defined by the nuclear analysis, and globally. Additionally, an Ewens–Watterson test[42], Chakraborty's test of population amalgamation[43] and Fu's F_s test[44] were conducted for each of those groups. To further identify population differentiation, the proportion of sequence variation (Φ_{ST}) was estimated for all pairs of populations and genomic clusters. Hierarchical AMOVA tests subsequently determined the significance of *a priori* subdivisions into colonies and genomic clusters. Calculation of Φ_{ST} and AMOVA tests were conducted in Arlequin applying 10,100 permutations and a Holm correction for multiple tests (for Φ_{ST}).

2.5 Nuclear Analysis

2.5.1 Genotype Likelihoods

The majority of population genomic analyses were based on genotype likelihoods as implemented in ANGSD v.0.931[45]. Prior to calculating genotype likelihoods, the quality of the mapped sequencing data was assessed in an ANGSD pre-run using *"-uniqueOnly 1 -remove_bads 1 -minMapQ 25 -maxDepth 800 -checkBamHeaders 1 -C 50 -baq 2 -doQsDist 1 -doDepth 1 -doCounts 1 -dumpCounts 2 -GL 1"*. For each individual, the depth of coverage per individual (0-20X) versus the proportion of sites was determined and visualized in R v.3.6[46] with a cannibalized script (https://github.com/z0on/2bRAD_denovo/blob/master/plotQC.R). As a result, an individual from the Isle of May (IOM001) was removed from the dataset due to low endogenous DNA content, low average depth of coverage and a large proportion of missing sites compared to all other samples (Supplementary Data 2, Figure S2a). Subsequently, the ANGSD pre-run was repeated with the same parameters, but without the removed individual. "Global Depth vs. No. of sites" and "Genotyping Rate Cutoff vs. No. of remaining sites" were calculated and plotted in R with the above-mentioned script to determine the appropriate cutoffs for depth and genotyping rate (Figure S2b).

Genotype likelihoods for SNPs covered in all individuals were calculated and filtered in ANGSD with *"-uniqueOnly 1 -remove_bads 1 -minMapQ 30 -minQ 30 -C 50 -baq 2 -checkBamHeaders 1 -HWE_pval 1e-2 -sb_pval 1e-5 -hetbias_pval 1e-5 -skipTriallelic 1 -minInd 71 -snp_pval 1e-6 -minMaf 0.05 -setMaxDepth 635 -setMinDepth 365 -doMajorMinor 1 -doMaf 1 -doCounts 1 -doGlf 2 -doHWE 1 -dosnpstat 1"*, resulting in the genotype likelihoods of 7,521,565 sites stored in *beagle* format.

The dataset was further pruned to account for linkage disequilibrium. Linkage expressed as the r^2 value was calculated for pairs of sites within 50 kb windows along all pseudo-chromosomes using ngsLD[47]. Linked sites ($R^2 > 0.2$) were clustered into larger groups using *mcl*[48] in the software OrthoMCL v2.0.92[49], and the most central site was selected as representative of each block for subsequent analyses[50]. Additionally, all variants located on the Z-pseudo-chromosome and "unplaced scaffolds" were excluded from the analyses yielding a final genotype likelihood panel consisting of 1,093,765 sites.

2.5.2 Population Structure

Genomic population structure was investigated using a Principal Component Analysis (PCA) of the genotype likelihood panel. PCAngsd v0.982[51] was run using default settings, followed by plotting the eigenvectors and eigenvalues of the two principal components explaining most observed genetic variation. Additional PCAs were computed for selected genomic sub-clusters containing subsets of the data following the same method.

Individual ancestry proportions were estimated using a maximum likelihood (ML) approach implemented in ngsAdmix v32[52] by setting the number of ancestral populations, K, from 1 to 10 and conducting 50 replicate runs for each K. The runs were clustered after similarity for each K and ancestry proportions were averaged within the major cluster using Clumpak[53] with default settings. The optimal value of K was chosen based on the method of Evanno[54] and biological validity. An additional "hierarchical" admixture analysis was conducted for a genomic sub-cluster using identical methods.

2.5.3 Phylogenetic Analyses

In order to be able to add an outgroup to the phylogenetic trees in this study, unpublished, raw 10xGenomics sequencing data used for the assembly of the embargoed razorbill genome (*Alca torda*, GCA_008658365.1) were mapped to the Atlantic puffin genome with PALEOMIX (settings as in *Sequencing and Data Processing)*, followed by calculating genotype likelihoods for the 1,093,765 sites of the final puffin dataset in ANGSD (settings as in *Genotype likelihoods*) after combining the razorbill and puffin data. Using this panel of genotype likelihoods, 100 bootstrap replicates of pairwise genetic distance matrices (p-distance) were calculated with ngsDist v1.0.8 by randomly sampling with replacement blocks of 20 SNPs. For each distance matrix replicate and the original distance matrix, a neighbor-joining (NJ) tree was built with FastMe v2.1.5[55], using the optimized BalME criterion followed by improving the initial tree topology with nearest-neighbor interchange (NNI) and subtree pruning and regrafting (SPR). The resulting trees were combined with IQTree v1.6.12[56].

Assessing and authenticating the topology of the NJ tree, a sample-based ML phylogenetic tree was built with Treemix v1.13[57]. At each of the 1,093,765 sites of the final puffin dataset, the consensus base was determined for each sample (including the razorbill as outgroup) with ANGSD (*-doIBS 2 -doMajorMinor1 -output01 0*) and converted into an allele count/frequency. Missing sites in the razorbill were randomly assigned a "1,0" or "0,1". Treemix was run 100 times at different seeds while applying a round of global rearrangements (*-global*), setting the razorbill at the root (*-root RAZ*), and turning off sample size correction (*-noss*). The topology of the replicate with the highest likelihood was assessed by generating 100 bootstrap replicates (*-bootstrap*) via resampling the data in blocks of 500 SNPs (*-k 500*). Bootstrap values were projected onto the "main" tree using IQTree.

Additionally, to infer patterns of population splitting and mixing, population-based ML trees including up to ten migration edges were generated in Treemix. The sample-wise allele frequency data matrix was converted into a population-wise data matrix by summing the allele counts of all samples of a population at each site. For each migration (0-10), 100 replicates were generated in Treemix applying the same settings as for the sample-based ML trees, except for using the -m (# of migration edges) flag and keeping the sample size correction turned on. The optimal number of migrations was picked using a quantitative approach implemented in the R package *OptM* (https://CRAN.R-project.org/package=OptM) by evaluating the distribution of explained variance, log likelihoods, and covariance with an increase in migration edges, and by applying the method of Evanno[54] and several different linear threshold models. For m_0 and m_{BEST}, the tree with the highest likelihood was selected and its topology was evaluated by generating 100 bootstrap replicates through resampling the data in blocks of 500 SNPs. Bootstrap values were projected onto each "main" tree using IQTree.

2.5.4 Tajima's D and Nucleotide Diversity

A set of neutrality tests and population statistics were calculated using colony-based one-dimensional (1D) folded Site-Frequency-Spectra (SFS). A set of sites covered in all individuals and passing several quality filters without removing rare alleles was selected in ANGSD with *"-uniqueOnly 1 -remove_bads 1 -minMapQ 30 -minQ 30 -dosnpstat 1 -C 50 -baq 2 -checkBamHeaders 1 -doHWE 1 -sb_pval 1e-5 -hetbias_pval 1e-5 -skipTriallelic 1 -minInd 71 -setmaxDepth 635 -setminDepth 365 -doMajorMinor 1 -doMaf 1 -doCounts 1".* The resulting dataset was further pruned by filtering out sites where heterozygote counts comprise more than

50% of all counts, as those could represent lumped paralogs[58] (also see https://github.com/ANGSD/angsd/issues/156), generating in a final count of 829,850,258 sites. Site allele frequency (SAF) likelihoods (*.saf.idx* file) were estimated for each population, genomic cluster, and globally in ANGSD (*angsd -sites sites_2do -GL 1 -doSaf 1)* with the puffin draft genome as ancestral sequence, followed by calculating folded 1D-SFS with *realSFS*. For each population, genomic cluster, and globally, Tajima's D, and nucleotide diversity ($\boldsymbol{\pi}$) were computed per pseudo-chromosome (*thetaStat do_stat* command in ANGSD) utilizing the per-site $\boldsymbol{\theta}$ estimates, which were determined by the *realSFS saf2theta* command using the folded 1D-SFS and SAF likelihoods. Nucleotide diversity per pseudo-chromosome was calculated by dividing the $\boldsymbol{\theta}_{pairwise}$ estimate by the number of sites. Significance of differences in nucleotide diversity between colonies and against the global mean was assessed with Wilcoxon Rank Sum test[59] applying the Holm correction[60].

2.5.5 Heterozygosity, Runs-of-Homozygosity, and Inbreeding

Individual genome-wide heterozygosity was calculated in ANGSD. Specifically, using the puffin draft genome as ancestral reference, the individual, folded, 1D SFS of 829,850,258 sites (see *Tajima's D and Nucleotide Diversity*) was estimated with ANGSD (-doSaf) and *realSFS*. Heterozygosity was calculated by dividing the number of polymorphic sites by the number of total sites present in the SFS. Statistical significance of differences in heterozygosity between populations was assessed with a global Kruskal-Wallis test[61], followed by a *post-hoc* Dunn test[62] applying the Holm correction[60].

The proportion of runs of homozygosity (RoH) within each puffin genome was computed by calculating local estimates of heterozygosity in sliding windows following the approach in Sánchez-Barreiro et al. (2020)[63]. A list containing 23,002 100 kbp sliding windows with a 50 kbp shift along the 25 pseudo-chromosomes (*-r parameter)* and the concatenated (all pseudo-chromosomes) site allele frequency likelihoods (*.saf.idx* files) from above were used for the estimation of the window-based SFS in *realSFS*. Local heterozygosity was calculated as above and the distribution of local heterozygosity per sample was visualized (Figure S18). The 10% quantile of the average local heterozygosity across all samples was defined as the cutoff for a "low heterozygosity region" and was set to 1.435663×10^{-3} (Figure S18). RoH were declared as all regions with at least two subsequent windows of low heterozygosity (below cutoff) and their final length was calculated as described in Sánchez-Barreiro et al. (2020)[63], i.e. $RoH_{length} = n_{windows} * 100\ kbp - ((n_{windows} - 1) * 50\ kbp)$. As a result, the minimum RoH length was 150 kbp, increasing in steps of 50 kbp. An individual inbreeding coefficient based on the RoH, F_{RoH} , was subsequently calculated as in Sánchez-Barreiro et al. (2020)[63] by computing the fraction of the entire genome falling into RoHs, with the entire genome being the total length of windows scanned, i.e. $Total\ Length = n_{All\_windows} * 100\ kbp - ((n_{All\_windows} - 1) * 50\ kbp)$. Statistical significance of differences in F_{RoH} between populations was assessed with a global Kruskal-Wallis test[61], followed by a *post-hoc* Dunn test[62] applying the Holm correction[60].

2.5.6 Gene flow and Isolation by Distance

Assessing potential landscape genetic patterns of Isolation-By-Distance (IBD) within the breeding range of the Atlantic puffin, the program EEMS[64] (estimated effective migration surfaces) was used to model the association between genetic and geographic data by visualizing the existing

population structure and highlighting regions of higher-than-average and lower-than-average historic gene flow. As input, a pairwise genetic distance matrix was calculated in ANGSD by sampling the consensus base (*-doIBS 2 -makeMatrix 1*) at the 1,093,765 filtered sites included in the genotype likelihood set (see *Population Structure*) for each sample. The matrix was fed into 10 independent runs of EEMS, each consisting of one MCMC chain of six million iterations with a two million iteration burn-in, 9999 thinning iterations, and 1000 underlying demes. The geographic population grid was outlined by a polygon drawn with http://www.birdtheme.org/useful/v3tool.html. As suggested by Petkova et al.[64], proposal variances thresholds were increased as follows to lower the proposal acceptance rate to a recommended level (10 - 40%): *mSeedsProposalS2 = 0.14*, *qSeedsProposalS2 = 0.6*, *mEffctProposalS2 = 0.9*, *qEffctProposalS2 = 0.006*. The EEMS output was visualized in R with code supplied by EEMS. One of the runs converged at a local as opposed to the global likelihood maximum and was excluded from the analysis (Figure S13b).

Supplementing the results of the EEMS analysis, a traditional IBD analysis was conducted by determining geographical and genetic distances between the 12 colonies and assessing the significance of the correlation between the two distance matrices with a Mantel test[65] and a Multiple Regression on distance Matrix (MRM)[66] analysis. In order to calculate F_{ST} as a proxy for genetic distance, two-dimensional (2D), folded SFS were computed for each population pair in *realSFS* by applying the per-population SAF likelihoods (*.saf.idx* files) generated above (see *Tajima's D and Nucleotide Diversity*). Subsequently, 2D SFS of population pairs were used together with the per-population SAF likelihoods to calculate the pairwise F_{ST} between each colony (*realSFS fst -whichFst 1 -fstout*). Pairwise F_{ST} values were converted to Slatkin's linearized F_{ST}[67]. Least Cost Path distances (paths between colonies only over water) between colony coordinates (latitude/longitude) were calculated using the R package *marmap*[68] and used as geographic distances. The Mantel test (999 permutations) and MRM analysis were performed with the R package *ecodist*[69]. A two-dimensional kernel density estimation (kde2d) with 300 grid points in each direction was run with the R package *MASS*[70] to visualize substructure in the landscape genetic patterns. All analyses for IBD were re-run on subsets of colonies by progressively removing the colony from the geographic and genetic distance matrices, whose removal led to the highest proportion of variance in genetic distance explained by geographic distance in the resulting regression model (Spitsbergen, Isle of May, Bjørnøya and Gannet Isl.). Hornøya was not removed due to comparably low Slatkin's linearized F_{ST} values at relatively large geographic distances.

A distance-based Redundancy Analysis (dbRDA[71] was conducted to corroborate the results of the MRM analyses and Mantel tests and to estimate the relative contribution of IBD and Isolation-By-Environment (IBE) to the observed Atlantic puffin population structure. The dbRDA was run between the genetic distance matrix containing pairwise Slatkin's linearized F_{ST} (dependent variables) versus geographic and environmental parameters (explanatory variables)[71]. A principal coordinate analysis (PCoA) was performed using the inter-colony pairwise Slatkin's linearized F_{ST} values, and the resulting principal component axes were kept as response variable after applying the Cailliez[72] correction to only retain positive eigenvalues[73]. To obtain uncorrelated geographic variables, the Least Cost Path distance matrix was transformed to positive Moran's Eigenvector Maps (MEMs)[74], using the R package *adespatial*[75] by setting the truncation threshold to the length of the longest edge of the minimum spanning tree. The sea-

surface-temperature (SST) at each colony during the months of April-August (breeding season) in the last 50 years[76] was retrieved from the HadiSST database[77] and the mean SST was used as environmental variable for each colony. Multicollinearity among geographic and environmental variables was accounted for by only retaining variables with a variance inflation factor < 5. A global dbRDA was run with all MEMs and the environmental variable and proportion of explained variance (adjusted R^2) as well as model probability were calculated using ANOVA tests with 999 permutations. For statistically significant global dbRDA models, the most significant variables (geographic or environmental) were selected via a stepwise regression using both forward and backward selection, and a stopping criterion[78]. The chosen variables then served as input for a reduced dbRDA, for which the marginal effect of each variable and its significance were tested using ANOVA tests with 999 permutations. A partial dbRDA with variance partitioning was conducted to estimate the independent contribution of the geographic and environmental variables in the optimized model and their significance, which also served as an estimation of the separate effects of IBD and IBE. Similar to the MRM analyses and Mantel tests, these analyses were repeated on subsets of colonies by progressively removing the colony from the geographic, environmental and genetic distance matrices, whose removal led to the highest proportion of variance explained in the resulting global dbRDA model. Optimized dbRDA model analyses were only conducted in cases where the selected spatial variables included both a geographic and environmental variable in order to be able to parse out effects of IBD and IBE. Methods and R code for the dbRDA were found at https://github.com/laurabenestan/db-RDA-and-db-MEM[79].

2.5.7 D- and ƒ3-statistic

Additional assessments of admixture and gene flow were conducted by calculating ƒ3-statistics and multi-population D-statistics (aka ABBA BABA test)[80]. Using the panel of population allele frequencies (see *Phylogenetic Analyses*), ƒ3-statistics were calculated in Treemix for each unique combination of ((A,B),C)) of the 12 puffin populations, where significantly negative values of the ƒ3 statistic (Z-score < -3) are evidence of admixture between population A and B in population C.

The D-statistics was calculated in ANGSD (-doAbbababa2) for each combination of ((A,B),C),Outgroup) using the 12 puffin populations. The statistic has a positive value if, in ((A,B),C),Outgroup), there is an excess of shared sites between A and Outgroup or B and C, and a negative value if there is an excess of shared sites between A and C or B and Outgroup, with statistical significance at -3 > Z-score > 3. The outgroup was generated in ANGSD by applying -doFasta 2 (with *-doCounts 1 -C 50 -minMapQ 30 -minQ 30* and min and max depth set to half and double the average depth) to the 10xGenomics sequencing data of the Razorbill mapped to the puffin reference genome (see *Phylogenetic Analyses*). The outgroup multi-*fasta* file was further filtered by removing the sequences corresponding to the mitochondrial region, the Z-chromosome, and "unplaced" scaffolds in the puffin reference genome.

2.5.8 Genome-wide patterns of genetic differentiation

To assess whether genetic differentiation is genome-wide or localized, patterns of pairwise F_{ST} values between genomic clusters were investigated. Two-dimensional (2D), folded SFS were computed for three genomic cluster/population pairs (Spitsbergen, Isle of May, Canada vs. Norway/Iceland/Faroe) in *realSFS* by applying the genomic cluster/population SAF likelihoods

(*.saf.idx* files) generated above (see *Tajima's D and Nucleotide Diversity*). Subsequently, 2D SFS of these pairs were used together with the SAF likelihoods to calculate the pairwise F_{ST} between each pair in sliding windows of 50 kb with 12.5 kb steps across the 25 pseudo-chromsomes (*realSFS fst -whichFst 1 -fstout* followed by *realSFS fst stats2 -win 50000 -step 12500*). The window size of 50 kb was chosen for sliding window analyses because LD decays to ca. 10% ($R < 0.025$) within this distance (Figure S19). In addition, the median pairwise F_{ST} was calculated using the resulting 93,778 sliding windows.

3. Results - Mitochondrial Population Structure

We obtained 192 mitogenomic SNPs of which 160 (83.33 %) were located in protein coding genes, five (2.60 %) in rRNAs, 19 (9.90 %) in tRNAs and six (3.13 %) in the Control Region (Table S8). The polymorphic sites across the mitogenomes defined 66 distinct haplotypes (Table S3), of which only four occurred in more than one individual (Figure 1b, Figure S3). Indicating high homoplasy, haplotype diversity was high (0.998 ± 0.003) and nucleotide diversity was low overall (π = 0.0008 ± 0.0004), with both being similar across colonies and nuclear genomic clusters (Table S3). We observed no significant geographic structure in mitogenome data (Figure 1b, Figures S3, S4). Similarly, while neither the global estimate of Φ_{ST} (0.006) nor pairwise Φ_{ST} values involving all colonies or genomic clusters were significant ($P > 0.05$, $n_{Colonies} = 12$, $n_{Clusters} = 4$), the majority of Φ_{ST} values including Spitsbergen were substantially higher compared to the rest (Table S4). Nevertheless, a set of neutrality tests and the haplotype network indicated inter-colony differences, as well as general recent population expansion (Table S3). While the Ewens-Watterson's tests were not significant for any of the colonies or genomic clusters, significant Tajima's D, significant Fu's Fs, or Chakraborty's tests for the "global" population, the cluster comprising mainland Norwegian, Icelandic and Faroese colonies, and the Canadian group indicated an excess number of alleles and absence of mutation-drift equilibrium, as would be expected from a recent population expansion (Table S3). Concordant with the global population expansion, the Fitchi haplotype network (Figure 1b, Figure S3) was presented in a distinctive star-shape, often associated with a population expansion, e.g. from a single refugium during the Last Glacial Maximum (LGM).

4. Supplementary Tables

Table S1: Length and number of placed scaffolds for each (pseudo-)chromosome of the Atlantic Puffin draft assembly. Scaffolds were mapped to the razorbill reference genome using minimap2 to order them into pseudo-chromosomes**.**

Puffin (Pseudo)chromosome	No. of Scaffolds	Total Length of Scaffolds without 'N' padding (bp)	Length of Razorbill Chromosomes (bp)	Difference (%)
Chromosome 01	1,876	234,966,019	215,872,496	8.84
Chromosome 02	1,379	176,321,017	165,051,286	6.83
Chromosome 03	1,017	133,497,470	125,510,139	6.36
Chromosome 04	567	88,537,706	82,438,829	7.40
Chromosome 05	725	76,161,884	70,992,871	7.28
Chromosome 06	541	55,750,312	52,539,765	6.11
Chromosome 07	513	52,461,240	47,750,090	9.87
Chromosome 08	537	47,829,909	43,861,690	9.05
Chromosome 09	377	47,463,729	43,770,548	8.44
Chromosome 10	374	45,442,885	41,489,931	9.53
Chromosome 11	551	42,383,409	38,974,675	8.75
Chromosome 12	339	38,810,983	35,546,317	9.18
Chromosome 13	511	38,802,013	34,322,904	13.05
Chromosome 14	179	14,714,561	13,223,547	11.28
Chromosome 15	205	14,765,580	13,149,485	12.29
Chromosome 16	268	10,588,656	9,767,604	8.41
Chromosome 17	284	9,490,077	8,686,195	9.25
Chromosome 18	184	8,770,925	8,379,326	4.67
Chromosome 19	454	7,553,894	8,133,896	-7.13
Chromosome 20	214	8,626,142	7,777,642	10.91
Chromosome 21	381	6,450,336	7,118,070	-9.38
Chromosome 22	279	6,110,852	6,270,964	-2.55
Chromosome 23	315	2,728,230	3,233,901	-15.64
Chromosome 24	238	2,117,903	2,563,298	-17.38
Chromosome 25	99	852,274	926,252	-7.99
Chromosome Z	921	105,477,361	84,526,827	24.79
Unplaced	2,000	17,061,156	N/A	N/A

Total Length of the Razorbill Genome	1,171,878,548	bp
Total Length of All Placed Scaffolds	1,276,675,367	bp
Total Length of All Scaffolds	1,293,736,523	bp
Total Number of Placed Scaffolds	13,328	
Total Number of Scaffolds	15,328	
Median Length of Unplaced Scaffolds	1,839	bp
Number of N's in Unplaced Scaffolds	9,166,580	
Number of non-N's in Unplaced Scaffolds	7,894,576	

Table S2: Manual annotation changes to the mitogenome of the Atlantic Puffin. Changes were made after manual inspection of the MITOS web server annotation. Reasons for changes and noteworthy attributes of several protein coding genes are mentioned. Start and stop coordinates are in BED format.

Orignal ID	Original Coordinates (bp)		Corrected ID	Corrected Coordinates (bp)		Notes
	Start	Stop		Start	Stop	
**NAD1**	2,792	3,763	**NAD1**	2,792	3,770	End extended by 7 bp.
**NAD3_a**	9,540	9,702	**NAD3**	9,525	9,874	Originally split because of a frameshift mutation. This mutation is well known and presumably skipped during translation (Mindell et al. 1998). Start was extended by 5AA/15bp.
**NAD3_b**	9,700	9,874				
**NAD4**	10,239	11,617	**NAD4**	10,239	11,622	End extended by 5 bp.
**NAD5**	11,801	13,637	-	-	-	Start codon is GTG instead of ATG (common in Charadriiformes; see Paton and Baker 2006).
**COX1**	5,387	6,938	-	-	-	Start codon is GTG instead of ATG (common in Charadriiformes; see Paton and Baker 2006).
**COX3**	8,672	9,456	-	-	-	Stop codon is just a single T. TAA stop codon is completed by the addition of 3' A residues to the mRNA via polyadenylation (Ojala et al. 1981).

References:

Mindell, D. P., M. D. Sorenson, and D. E. Dimcheff. 1998. "An Extra Nucleotide Is Not Translated in Mitochondrial ND3 of Some Birds and Turtles." Molecular Biology and Evolution 15 (11): 1568–71.

Paton, Tara A., and Allan J. Baker. 2006. "Sequences from 14 Mitochondrial Genes Provide a Well-Supported Phylogeny of the Charadriiform Birds Congruent with the Nuclear RAG-1 Tree." Molecular Phylogenetics and Evolution 39 (3): 657–67.

Ojala, D., J. Montoya, and G. Attardi. 1981. "tRNA Punctuation Model of RNA Processing in Human Mitochondria." Nature 290 (5806): 470–74.

Table S3: Diversity and neutrality statistics for nuclear and mitochondrial genomes across 12 Atlantic Puffin colonies. Values in bold are genomic clusters identified by the nuclear data analysis. Nuclear statistics were calculated using colony-based one-dimensional (1D) folded Site-Frequency-Spectra (SFS). A set of sites covered in all individuals and passing several quality filters without removing rare alleles was selected in ANGSD and the resulting dataset was further pruned by filtering out sites where heterozygote counts comprise more than 50% of all counts. Site allele frequency (SAF) likelihoods were estimated for each population, genomic cluster, and globally in ANGSD followed by calculating folded 1D-SFS with *realSFS*. Tajima's D and nucleotide diversity (**π**) were computed per pseudo-chromosome utilizing the per-site **θ** estimates. All mitogenome statistics and tests were calculated in Arlequin v.3.5. h = haplotype diversity. π = nucleotide diversity. SD = standard deviation.

		Mitogenomes							Nuclear Genomes	
Colony	Sample Size	No. of distinct haplotypes	h ± SD	π ± SD (x 10^{-3})	Tajima's D	Ewens-Watterson's F	Chakraborty's Test[1]	Fu's Fs	π ± SD (x 10^{-3})	Tajima's D ± SD
Breiðafjörður	6	6	1.000 +/- 0.096	0.62 +/- 0.38	-0.89	N.A.	6/4.93	-0.88	3.28 +/- 0.30	0.04 +/- 0.05
Faroe	6	6	1.000 +/- 0.096	0.94 +/- 0.57	-0.58	N.A.	6/5.23	-0.29	3.28 +/- 0.29	0.00 +/- 0.05
Grímsey	6	6	1.000 +/- 0.096	0.80 +/- 0.48	-1.22	N.A.	6/5.12	-0.52	3.28 +/- 0.30	0.00 +/- 0.05
Hornøya	6	6	1.000 +/- 0.096	0.78 +/- 0.47	-0.75	N.A.	6/5.10	-0.56	3.23 +/- 0.27	0.05 +/- 0.04
Papey	6	5	0.933 +/- 0.122	0.82 +/- 0.50	-0.26	0.22	5/5.14	1.36	3.29 +/- 0.29	-0.01 +/- 0.05
Røst	6	5	0.933 +/- 0.122	0.55 +/- 0.34	-0.50	0.22	5/4.83	0.69	3.24 +/- 0.29	0.04 +/- 0.04
Vestmannaeyjar	6	6	1.000 +/- 0.096	0.98 +/- 0.59	-1.13	N.A.	6/5.26	-0.24	3.30 +/- 0.29	-0.01 +/- 0.04
**Main**	**42**	**39**	**0.997 +/- 0.006**	**0.79 +/- 0.40**	**-2.17***	**0.03**	**39/19.43**	**-24.02***	**3.27 +/- 0.29****	**0.02 +/- 0.05 ****
Gannet Isl.	6	6	1.000 +/- 0.096	0.70 +/- 0.43	-0.42	N.A.	6/5.03	-0.70	3.26 +/- 0.29	0.07 +/- 0.04
Gull Isl.	6	6	1.000 +/- 0.096	0.76 +/- 0.46	-0.89	N.A.	6/5.08	-0.59	3.13 +/- 0.71	0.06 +/- 0.06
**Canada**	**12**	**12**	**1.000 +/- 0.034**	**0.72 +/- 0.39**	**-1.23**	**N.A.**	**12/8.59**	**-4.23***	**3.28 +/- 0.29**	**-0.13 +/- 0.06**
**Isle of May**	**5**	**5**	**1.000 +/- 0.127**	**0.83 +/- 0.53**	**-0.87**	**N.A.**	**5/4.41**	**0.09**	**3.27 +/- 0.30**	**0.12 +/- 0.05**
**Spitsbergen**	**6**	**5**	**0.933 +/- 0.122**	**0.67 +/- 0.41**	**0.45**	**0.22**	**6/4.99**	**1.02**	**3.09 +/- 0.28**	**0.43 +/- 0.06**
Bjørnøya	6	6	1.000 +/- 0.096	0.78 +/- 0.47	-1.34	N.A.	6/5.10	-0.56	3.23 +/- 0.28	0.07 +/- 0.04
**Total**	**71**	**66**	**0.998 +/- 0.003**	**0.77 +/- 0.39**	**-2.33***	**0.02**	**66/24.73**	**-24.31***	**3.24 +/- 0.34****	**0.07 +/- 0.12****

*Significant deviation from neutral expectation at α=0.05

**Mean of individual colony values due to a known unresolved bug in the ANGSD program (*thetaStat do_stat* with a large SFS results in -NaN)

[1]Number of haplotype sampled/expected number of haplotypes

N.A. = The test is impossible because all haplotypes are different

Table S4: Pairwise genetic distances between Atlantic Puffin colonies and genomic clusters identified by the analysis of nuclear and mitochondrial genomes. Matrices contain pairwise F_{ST} (top diagonal) and Φ_{ST} (bottom diagonal) values between A) 12 colonies and B) four genomic clusters. None of the Φ_{ST} values are statistically significant ($P < 0.05$). Pairwise F_{ST} values are based on folded two-dimensional (2D) Site-Frequency-Spectra (SFS), which were calculated using ANGSD as follows: Site allele frequency (SAF) likelihoods were estimated for each colony using a set of sites covered in all individuals and passing several quality filters (without removing rare alleles) including removing sites with heterozygote counts comprising more than 50% of all counts. Folded, 2D SFS were computed for each population pair in *realSFS* (ANGSD) and used together with the per-population SAF likelihoods to calculate the pairwise F_{ST} between each colony. The proportion of sequence variation (Φ_{ST}) was estimated for all pairs of populations using the mitogenome SNPs. Calculation of Φ_{ST} was conducted in Arlequin applying 10,100 permutations and a Holm correction for multiple tests. None of them were significant.

a

	Breiðafjörður	Faroe	Grímsey	Hornøya	Papey	Røst	Vestmannaeyjar	Gannet Isl.	Gull Isl.	Isle of May	Spitsbergen	Bjørnøya
**Breiðafjörður**	-	0.001	0.000	0.002	0.000	0.001	0.000	0.005	0.004	0.006	0.033	0.004
**Faroe**	-0.059	-	0.002	0.002	0.001	0.002	0.001	0.006	0.004	0.007	0.034	0.005
**Grímsey**	-0.029	-0.002	-	0.002	0.001	0.002	0.001	0.006	0.005	0.007	0.034	0.004
**Hornøya**	-0.070	0.028	-0.027	-	0.002	0.001	0.002	0.007	0.005	0.007	0.034	0.004
**Papey**	0.028	-0.028	0.089	0.135	-	0.002	0.001	0.005	0.004	0.007	0.035	0.004
**Røst**	-0.018	0.031	0.048	0.012	0.160	-	0.002	0.006	0.005	0.007	0.034	0.004
**Vestmannaeyjar**	-0.044	-0.027	-0.017	-0.025	0.035	0.054	-	0.005	0.004	0.007	0.034	0.004
**Gannet Isl.**	-0.050	-0.038	0.069	0.030	0.055	-0.023	0.026	-	0.002	0.011	0.039	0.009
**Gull Isl.**	-0.088	-0.078	-0.032	-0.042	0.016	0.002	-0.071	-0.033	-	0.010	0.037	0.007
**Isle of May**	-0.029	0.019	-0.014	-0.067	0.129	0.052	-0.093	0.061	-0.073	-	0.040	0.010
**Spitsbergen**	0.024	0.051	0.069	0.045	0.165	-0.021	0.081	0.058	0.029	0.082	-	0.018
**Bjørnøya**	-0.098	-0.051	-0.019	-0.023	0.012	-0.003	-0.049	-0.009	-0.076	-0.019	-0.026	-

b

	Main	Canada	Isle of May	Spitsbergen
**Main**	-	0.004	0.006	0.033
**Canada**	-0.016	-	0.010	0.037
**Isle of May**	-0.001	0.012	-	0.040
**Spitsbergen**	0.044	0.048	0.082	-

Table S5: Overview of Isolation By Distance (IBD) analyses among 12 Atlantic puffin colonies. Pairwise Slatkin's linearized F_{ST} values were used as genetic distances and pairwise Least Cost Path distances (over water only) as geographic distances. The significance of the correlation between the two distance matrices was assessed with a Mantel test and a Multiple Regression on Distance Matrix (MRM) analysis. For the global distance-based redundancy analysis (dbRDA) the explanatory variables included the geographic distance matrix transformed to Moran's Eigenvector Maps and the mean sea surface temperature at each puffin colony in the breeding season in the last 50 years. For all three analyses, colonies were sequentially removed from the dataset to increase the proportion of explained genetic variance. Variance Explained (MRM) = Proportion of variance in genetic distance explained by geographic distance. Variance Explained (dbRDA) = Proportion of variance in genetic distance explained by spatial parameters. *Gannet Island was not removed from the global dbRDA as it resulted in a non-significant model with substantially decreased explained variance.

Removed colonies		MRM Analysis			Mantel Test		Global dbRDA		
		Variance Explained (%)	Regression Coefficient (x 10^{-6})	P-value	R-value	P-value	Variance Explained (%)	F-value	P-value
-		3.69	1.91	0.354	0.192	0.172	18.76	1.85	0.075
Spitsbergen		37.58	1.37	0.003	0.613	0.002	41.73	3.39	0.004
Bjørnøya		4.83	2.37	0.446	0.220	0.196	21.39	1.91	0.216
Breiðafjörður		2.49	1.55	0.457	0.158	0.192	17.14	1.69	0.159
Faroe		2.82	1.68	0.463	0.168	0.184	17.29	1.70	0.106
Grímsey		2.66	1.62	0.472	0.163	0.192	26.53	2.20	0.033
Hornøya		7.77	2.94	0.213	0.279	0.18	38.91	3.12	0.008
Papey		2.52	1.59	0.486	0.159	0.18	17.64	1.71	0.125
Røst		4.76	2.21	0.409	0.218	0.19	17.37	1.70	0.105
Vestmannaeyjar		2.35	1.52	0.506	0.153	0.205	19.43	1.80	0.086
Gannet Isl.		3.12	1.94	0.338	0.177	0.105	21.24	2.35	0.084
Gull Isl.		4.92	2.72	0.232	0.222	0.12	16.62	1.66	0.15
Isle of May		3.28	1.79	0.426	0.181	0.175	17.55	1.71	0.128
	Bjørnøya	38.60	1.39	0.006	0.621	0.005	41.37	3.12	0.015
	Breiðafjörður	34.14	1.23	0.005	0.584	0.006	44.06	3.36	0.002
	Faroe	37.39	1.38	0.006	0.612	0.004	42.06	3.18	0.004
	Grímsey	33.41	1.27	0.011	0.578	0.007	44.89	3.44	0.004
	Hornøya	45.66	1.63	0.003	0.676	0.005	42.46	4.32	0.001
Spitsbergen +	Papey	33.89	1.30	0.011	0.582	0.01	42.76	3.24	0.001
	Røst	39.39	1.43	0.006	0.628	0.006	43.33	3.29	0.004
	Vestmannaeyjar	34.51	1.29	0.008	0.587	0.004	48.11	3.78	0.003
	Gannet Isl.	27.01	1.19	0.091	0.520	0.094	28.16	2.76	0.046
	Gull Isl.	37.68	1.67	0.029	0.614	0.034	36.09	2.69	0.023
	Isle of May	65.92	1.37	0.002	0.812	0.001	50.86	4.11	0.008
	Bjørnøya	77.49	1.39	0.002	0.880	0.001	59.87	4.98	0.014
	Breiðafjörður	65.33	1.26	0.003	0.808	0.001	49.95	3.66	0.009
	Faroe	64.13	1.34	0.003	0.801	0.001	49.60	3.62	0.014
	Grímsey	62.37	1.31	0.001	0.790	0.002	57.01	4.54	0.004
Spitsbergen/	Hornøya	77.87	1.62	0.001	0.882	0.001	54.66	5.82	0.006
Isle of May +	Papey	62.47	1.32	0.001	0.790	0.002	52.40	3.94	0.007
	Røst	67.93	1.42	0.004	0.824	0.002	51.93	3.88	0.009
	Vestmannaeyjar	63.52	1.32	0.002	0.797	0.002	49.45	3.61	0.014
	Gannet Isl.	58.79	1.16	0.004	0.767	0.001	36.31	3.28	0.002
	Gull Isl.	63.59	1.60	0.001	0.797	0.003	45.88	3.26	0.005
	Breiðafjörður	79.50	1.31	0.002	0.892	0.002	61.27	4.69	0.033
	Faroe	76.54	1.39	0.002	0.875	0.003	59.57	4.44	0.027
	Grímsey	75.96	1.36	0.002	0.872	0.002	61.80	4.78	0.035
Spitsbergen/	Hornøya	83.91	1.59	0.007	0.916	0.004	64.75	7.43	0.015
Isle of May/	Papey	75.38	1.38	0.003	0.868	0.003	63.23	5.01	0.02
Bjørnøya +	Røst	75.10	1.39	0.004	0.867	0.004	63.68	5.09	0.013
	Vestmannaeyjar	77.59	1.38	0.002	0.881	0.002	59.30	4.40	0.055
	Gannet Isl.	84.98	1.19	0.001	0.922	0.002	39.64	3.30	0.082
	Gull Isl.	76.77	1.61	0.001	0.876	0.002	50.75	3.40	0.029
	Breiðafjörður	89.10	1.12	0.001	0.944	0.002			
	Faroe	85.43	1.23	0.004	0.924	0.009			
Spitsbergen/	Grímsey	84.37	1.17	0.004	0.919	0.004			
Isle of May/	Hornøya	90.62	1.37	0.009	0.952	0.009		*	
Bjørnøya/	Papey	85.11	1.20	0.003	0.923	0.001			
Gannet Isl. +	Røst	83.80	1.19	0.008	0.915	0.006			
	Vestmannaeyjar	85.64	1.20	0.005	0.925	0.003			
	Gull Isl.	60.08	0.76	0.006	0.775	0.012			

Table S6: Detailed results of significance tests for the optimized and partial distance-based redundancy analysis (dbRDA) on 12 Atlantic puffin colonies. The genetic distance matrix consisted of pairwise Slatkin's linearized F_{ST} values. Explanatory variables included a geographic distance matrix consisting of pairwise Least Cost Path distances (only over water) transformed to Moran's Eigenvector Maps and the mean sea surface temperature at each puffin colony in the breeding season in the last 50 years. After removing the Spitsbergen colony from the dataset, the global dbRDA was significant. In the resulting optimized model, a geographical and environmental variable significantly contributed to the observed genetic variation. Their individual contribution was estimated in a partial dbRDA followed by variation partitioning. Variance Explained = Proportion of variance in genetic distance explained by spatial features. D.f. = Degrees of Freedom. MEM = Moran's Eigenvector Map. SSTmean = Mean sea surface temperature at each puffin colony in the breeding season in the last 50 years.

		Optimized dbRDA				Partial dbRDA			
		Variance Explained (%)	D.f.	F-value	P-value	Variance Explained (%)	D.f.	F-value	P-value
Spitsbergen Removed	MEM1	29.38	1	5.16	0.006	28.66	1	5.35	0.005
	SSTmean	11.37	1	2.73	0.018	11.37	1	2.73	0.024
	Residual	59.25	7	-	-	59.25	7	-	-

Table S7: Significant admixture signal between 12 Atlantic Puffin colonies as revealed by f3-statistics. Statistics were calculated in Treemix using the consensus base at each of 1,093,765 polymorphic nuclear sites for each of the 71 puffins, and determined for each unique combination of the ((A,B),C)) topology involving the 12 puffin populations. Only significant (Z-score < -3) topologies are shown in the table, each providing evidence of admixture between population A and B in population C/"Admixed".

Admixed	A	B	f3	stdErr	Zscore
BJO	PAP	SPI	-0.00150	0.0001	-23.79
BJO	GUL	SPI	-0.00148	0.0001	-23.05
BJO	GRI	SPI	-0.00147	0.0001	-23.41
BJO	FAR	SPI	-0.00144	0.0001	-22.88
BJO	GAN	SPI	-0.00143	0.0001	-22.63
BJO	HOR	SPI	-0.00143	0.0001	-22.46
BJO	WES	SPI	-0.00142	0.0001	-22.44
BJO	ROS	SPI	-0.00141	0.0001	-22.94
BJO	BRE	SPI	-0.00141	0.0001	-22.53
BJO	IOM	SPI	-0.00135	0.0001	-20.75

5. Supplementary Figures

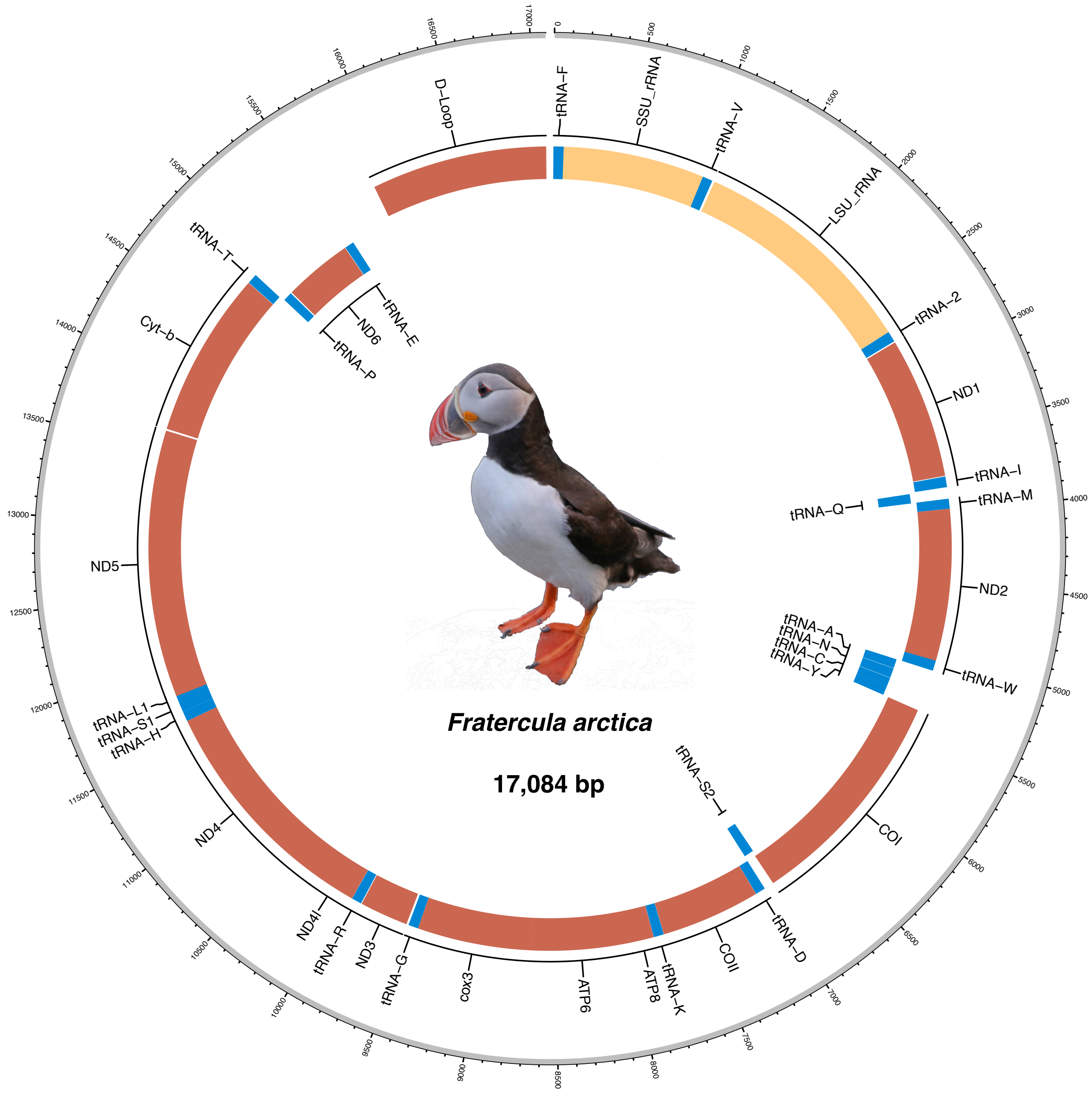

Figure S1: Circular representation of the annotated mitogenome of the Atlantic puffin. Protein coding regions are highlighted in red, tRNAs in blue, and rRNAs in yellow. Outer ring depicts position in bp. Annotation was performed with the MITOS web server and the plot was produced with shinyCircos. Puffin image credit: Annemarie Loof.

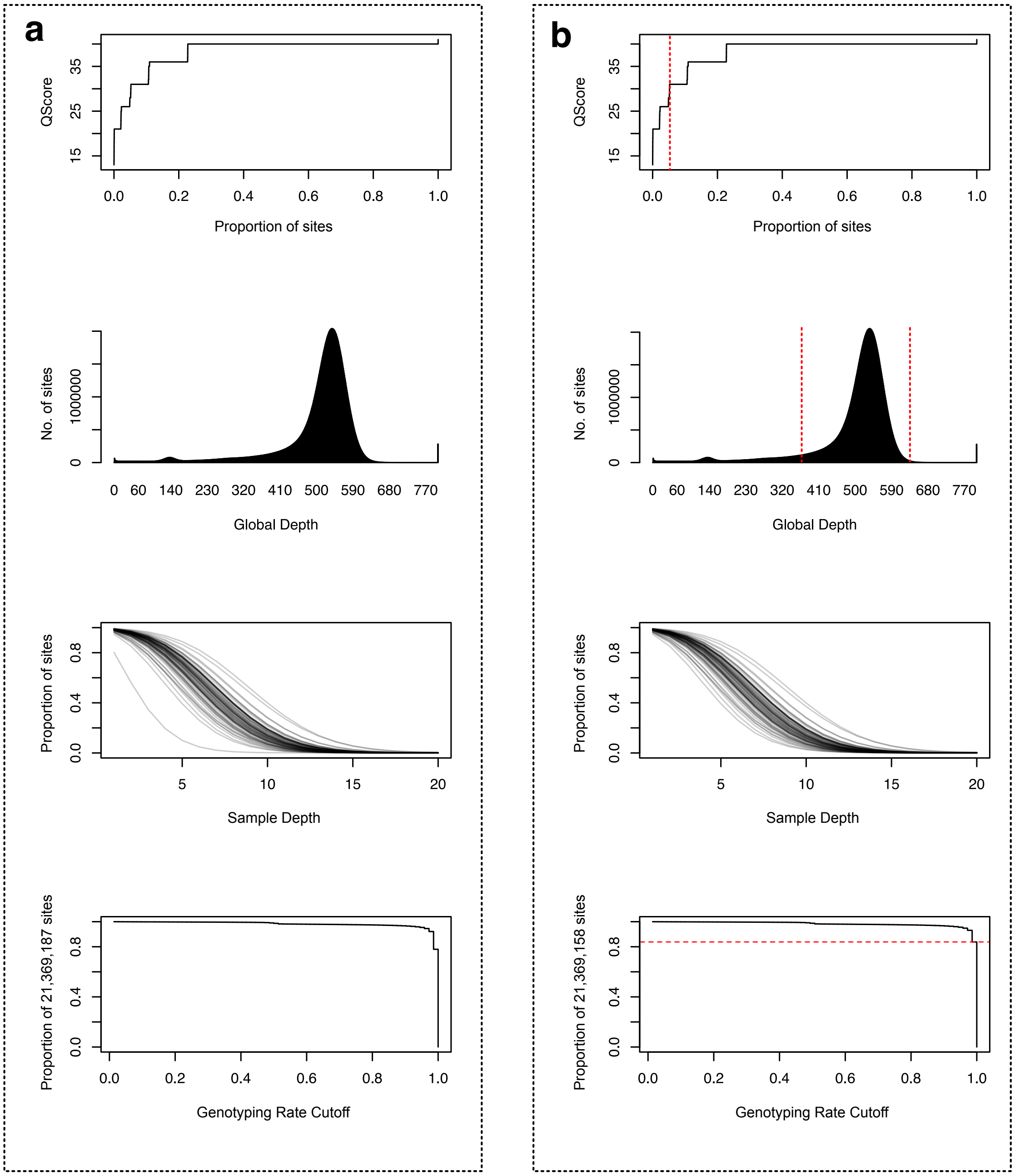

Figure S2: Mapping quality control of 72 Atlantic puffin samples aligned to the newly assembled draft genome. One sample had very low endogenous DNA content and low coverage across all sites in a) and was removed in b). Cutoffs for genotyping rate, global depth and QScore (red lines) were chosen for downstream genotype likelihood calculations.

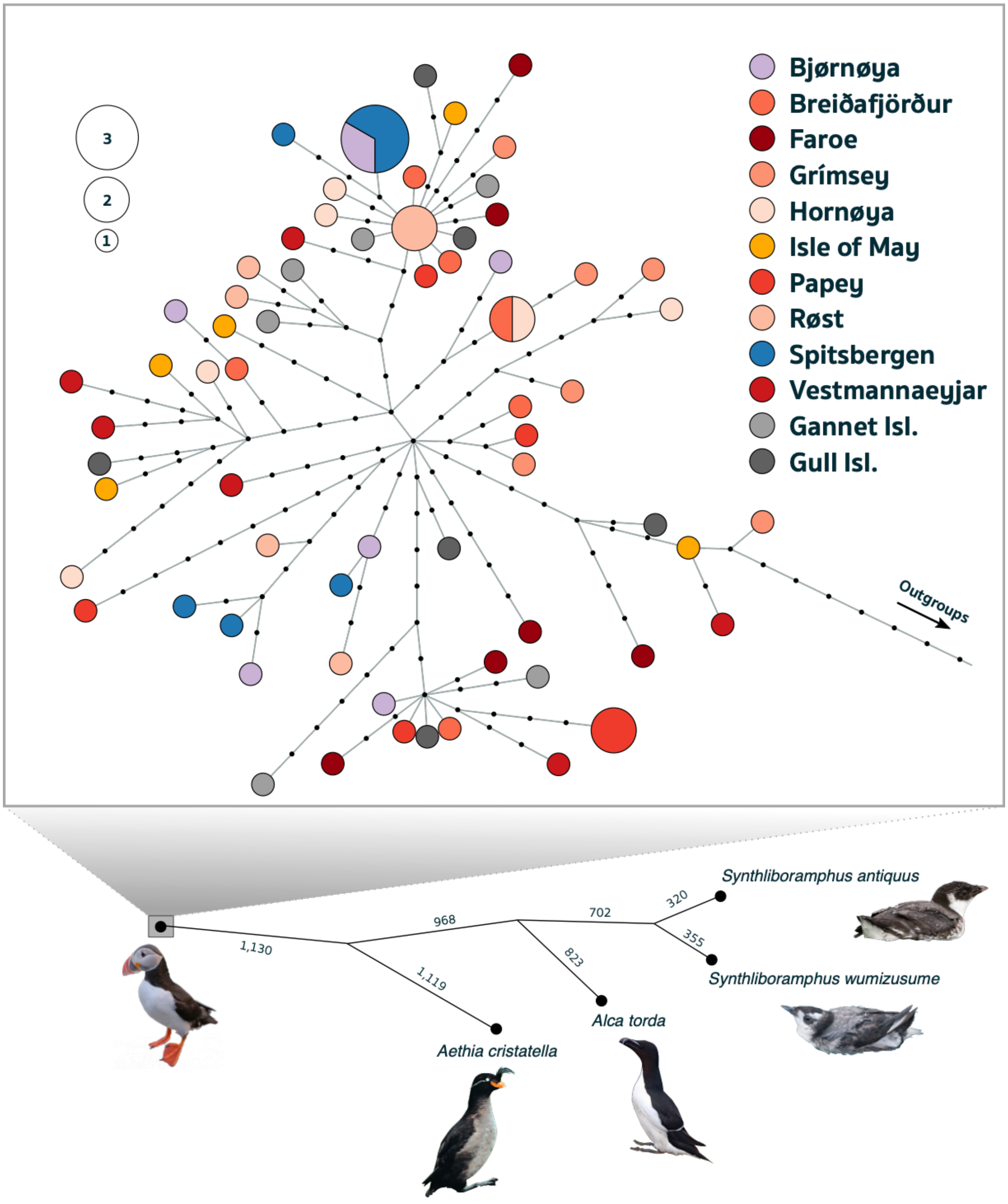

Figure S3: Haplotype network for 71 Atlantic puffin mitogenomes. The network was constructed using the program Fitchi and a Maximum Likelihood tree generated in IQTree. It contains 66 unique haplotypes identified by 192 SNPs. Sizes of circles are proportional to haplotype abundance. Black dots represent inferred haplotypes that were not found in the present sampling. Different colonies are indicated using different colors consistent with the remaining manuscript. Numbers in the outgroup tree refer to the number of substitutions between mitogenomes. Outgroup images were used under the Creative Commons license and retrieved from following authors on Wiki Commons or flickr: Eric Ellingson (Ancient Murrelet), “pseudolapiz” (Japanese Murrelet), Gunther Tschuch (Razorbill) and F. Deines (Crested Auklet).

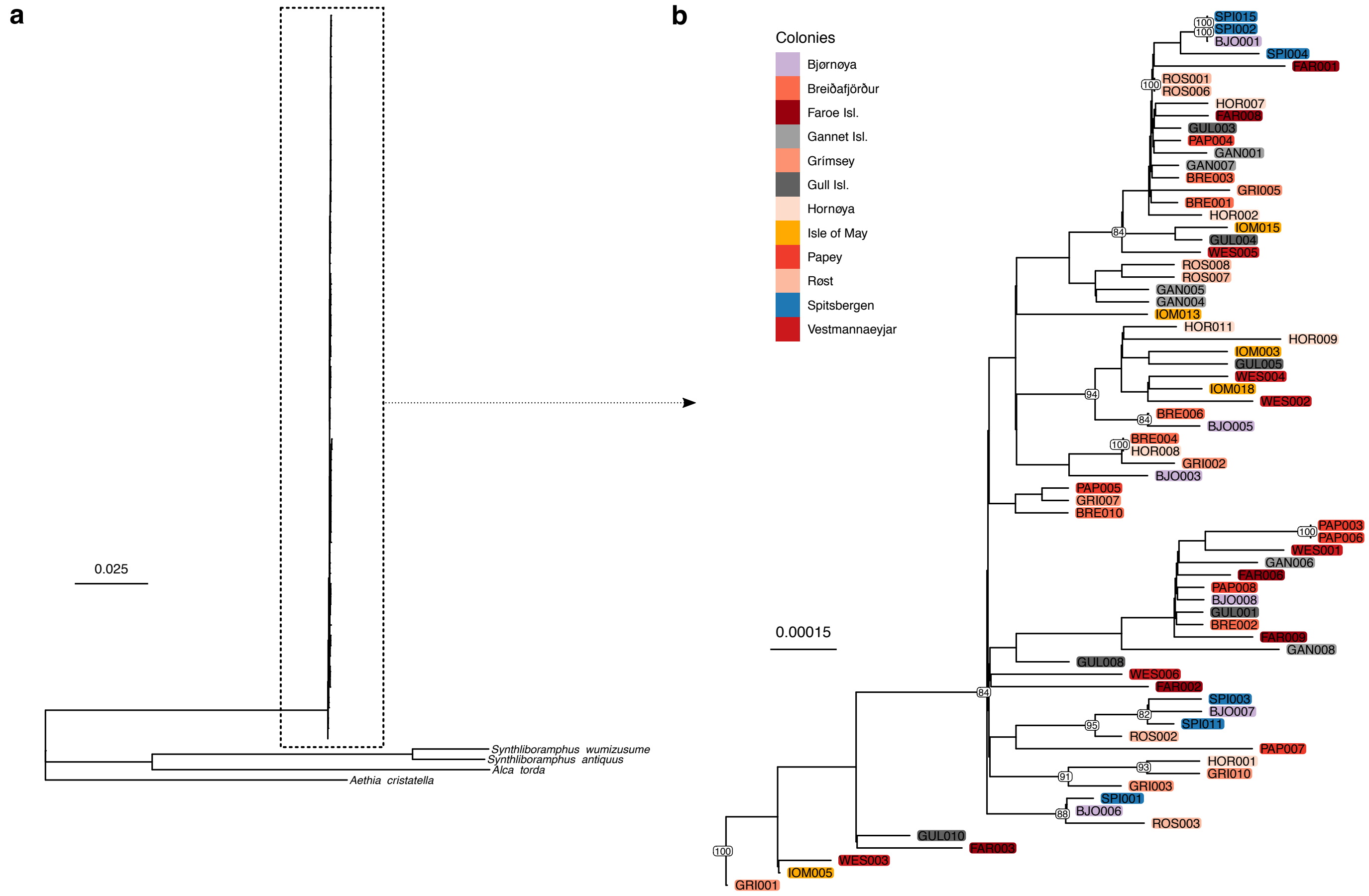

Figure S4: Mitogenome phylogeny including 12 Atlantic puffin colonies across the species' breeding range. The maximum likelihood tree was generated in IQTree using an alignment of 71 puffin and four outgroup species mitogenomes. The outgroups are members of the same bird family (Alcidae). The alignment was split into seven partitions, i.e. one partition for a concatenated alignment of each of the three codon positions of the protein coding genes, one partition for the concatenated alignment of the rRNA regions, one partition for the concatenated alignment of the tRNAs, one partition for the alignment of the control region, and one partition for the concatenated alignment of the "intergenic" regions. The tree was visualized a) with and b) without the outgroups. Different colonies are indicated using different colors consistent with the main manuscript and node labels show bootstrap support.

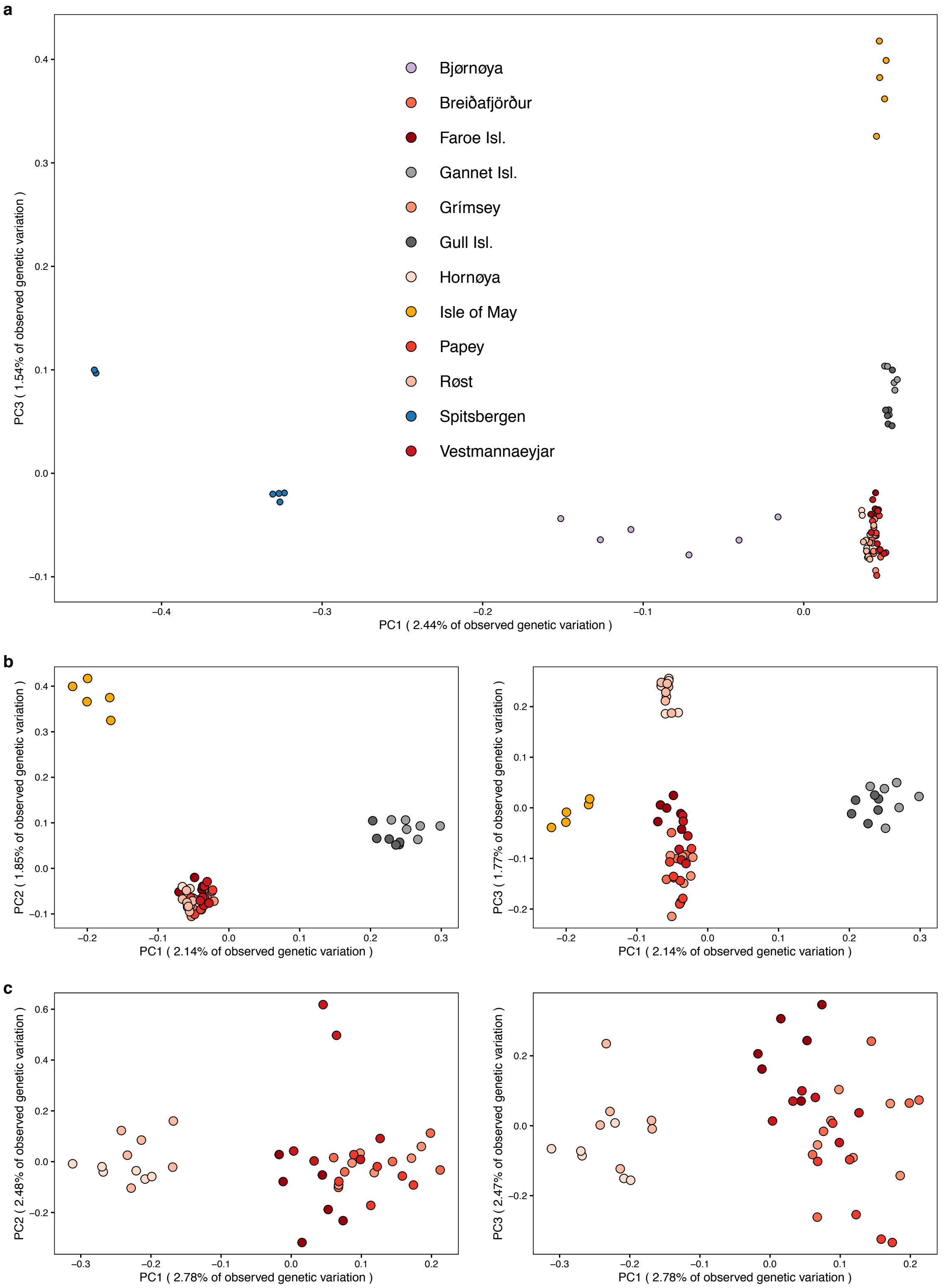

Figure S5: Principal component analyses (PCAs) of genotype likelihoods across 12 Atlantic puffin colonies. The data consisted of 71 puffin samples and 1,093,765 polymorphic nuclear sites and was projected onto a) PC1 and PC3, but was also subsampled to b) exclude the Spitsbergen and Bjørnøya colonies and to c) only include the mainland Norwegian, Icelandic and Faroese colonies. Different colonies are indicated using different colors consistent with the main manuscript.

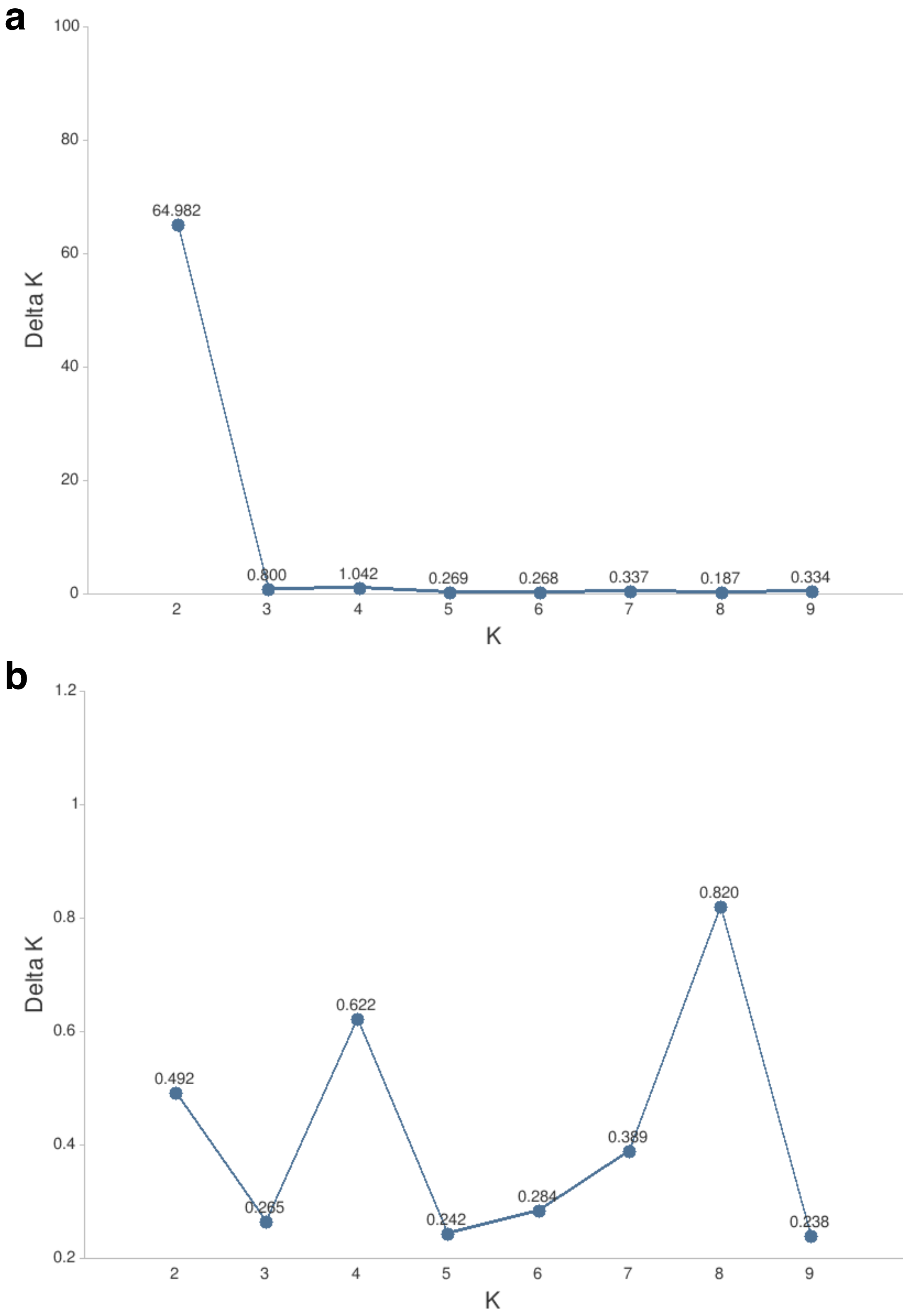

Figure S6: Delta K as a function of the no. of ancestral clusters (K) as calculated by the method of Evanno et al. (2005) for K = 1-9. The optimal K for the admixture analysis including a) a genotype likelihood panel consisting of 71 individuals and 1,093,765 polymorphic nuclear sites and b) a subset of this dataset comprised of 59 individuals (after removing individuals from Spitsbergen and Bjørnøya) is determined by the largest delta K(s).

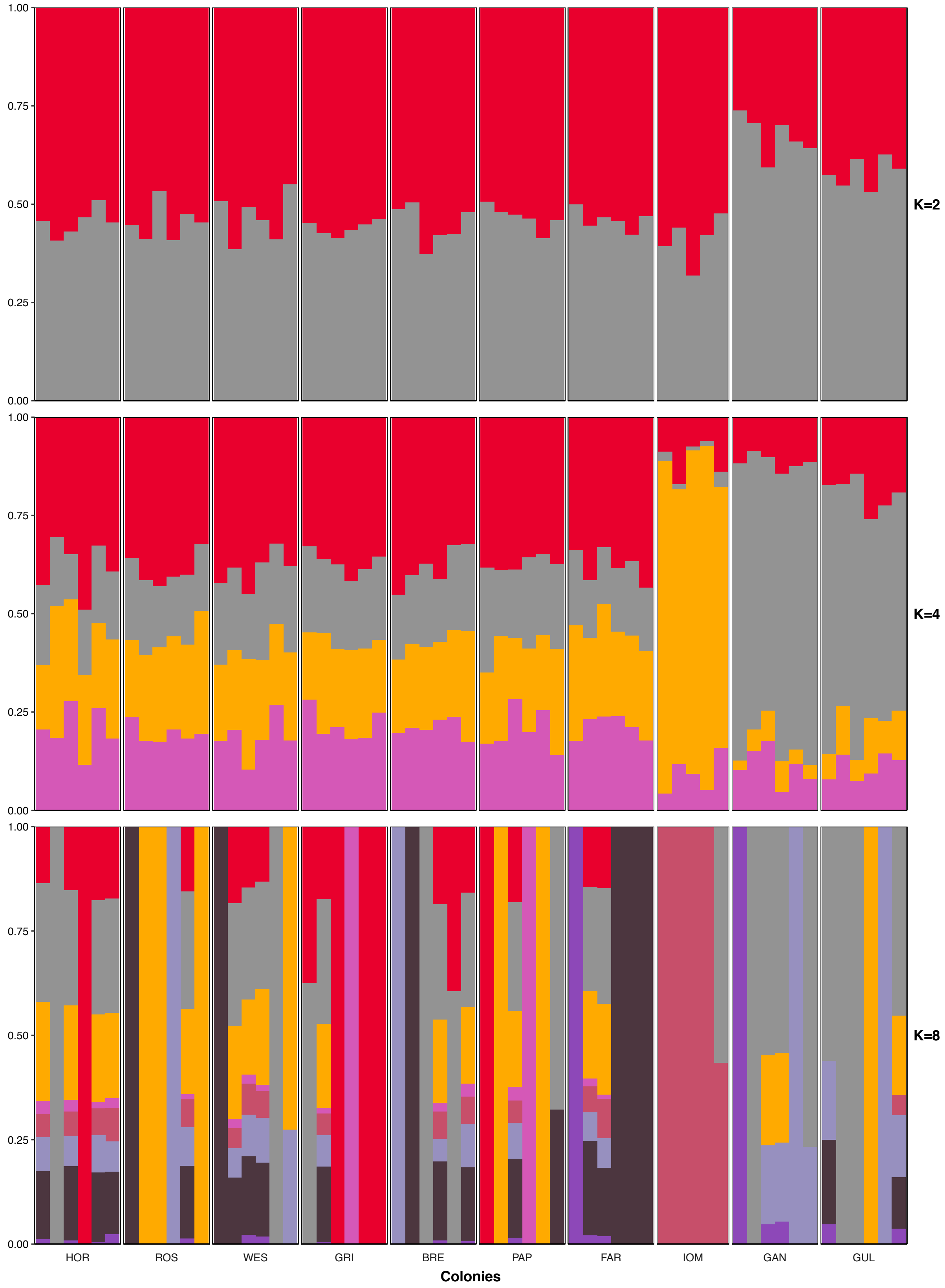

Figure S7: CLUMPAK-averaged admixture plots of the best K's for a subset of the Atlantic puffin genotype likelihood panel after excluding Spitsbergen and Bjørnøya individuals. Each column represents a sample and colonies are separated by solid white lines. Optimal K's were determined by the method of Evanno et al. (2005) (See Figure S6b).

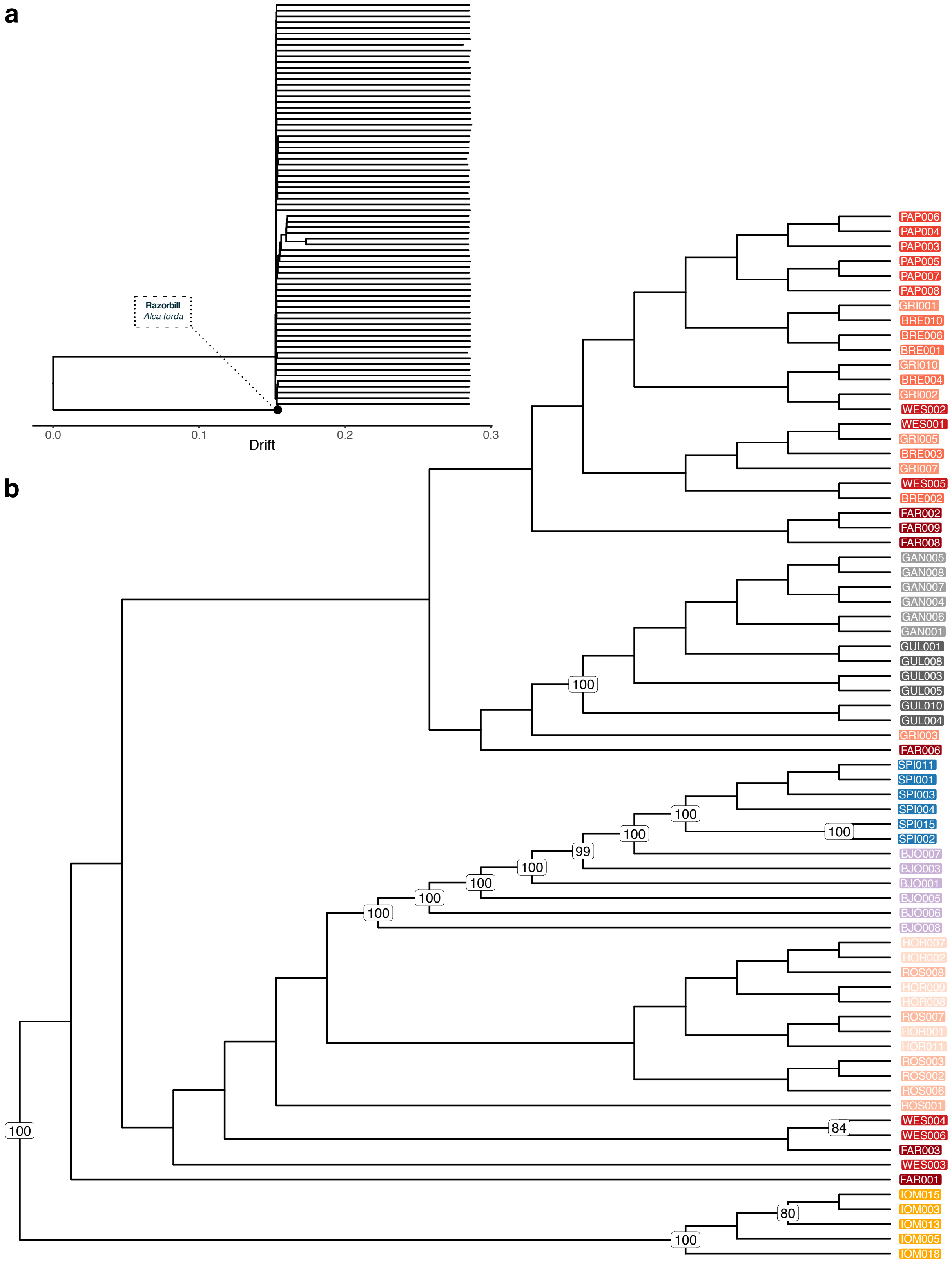

Figure S8: Individual-based Treemix analysis of 71 Atlantic puffins. The maximum likelihood analysis used allele frequencies at 1,093,765 polymorphic nuclear sites and the razorbill as outgroup. The output was visualized a) with and b) without branch lengths and outgroup. Different colonies are indicated using different colors consistent with the main manuscript. Node labels show bootstrap support > 80.

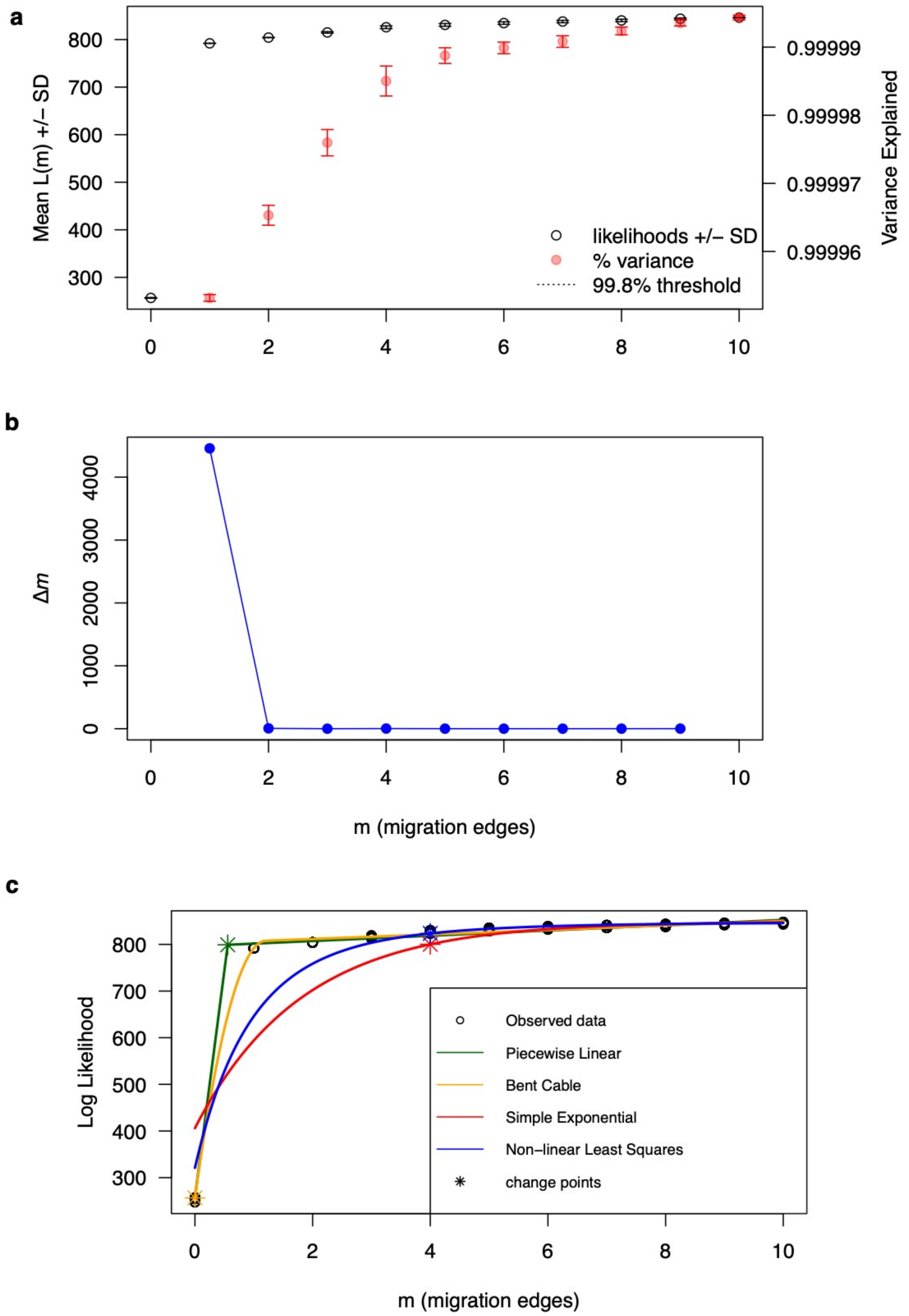

Figure S9: Estimation of the optimal number of migration edges (m) for a Treemix generated population-based maximum likelihood tree using optM. OptM utilized the output of 100 replicate Treemix runs across m = 1-10. The program estimated the best m using a) the distribution of the mean log likelihoods and % explained variance for each m and b) an *ad hoc* statistic based on the second order rate of change in the log likelihood weighted by the standard deviation. c) The selection of the best m is checked by various threshold models.

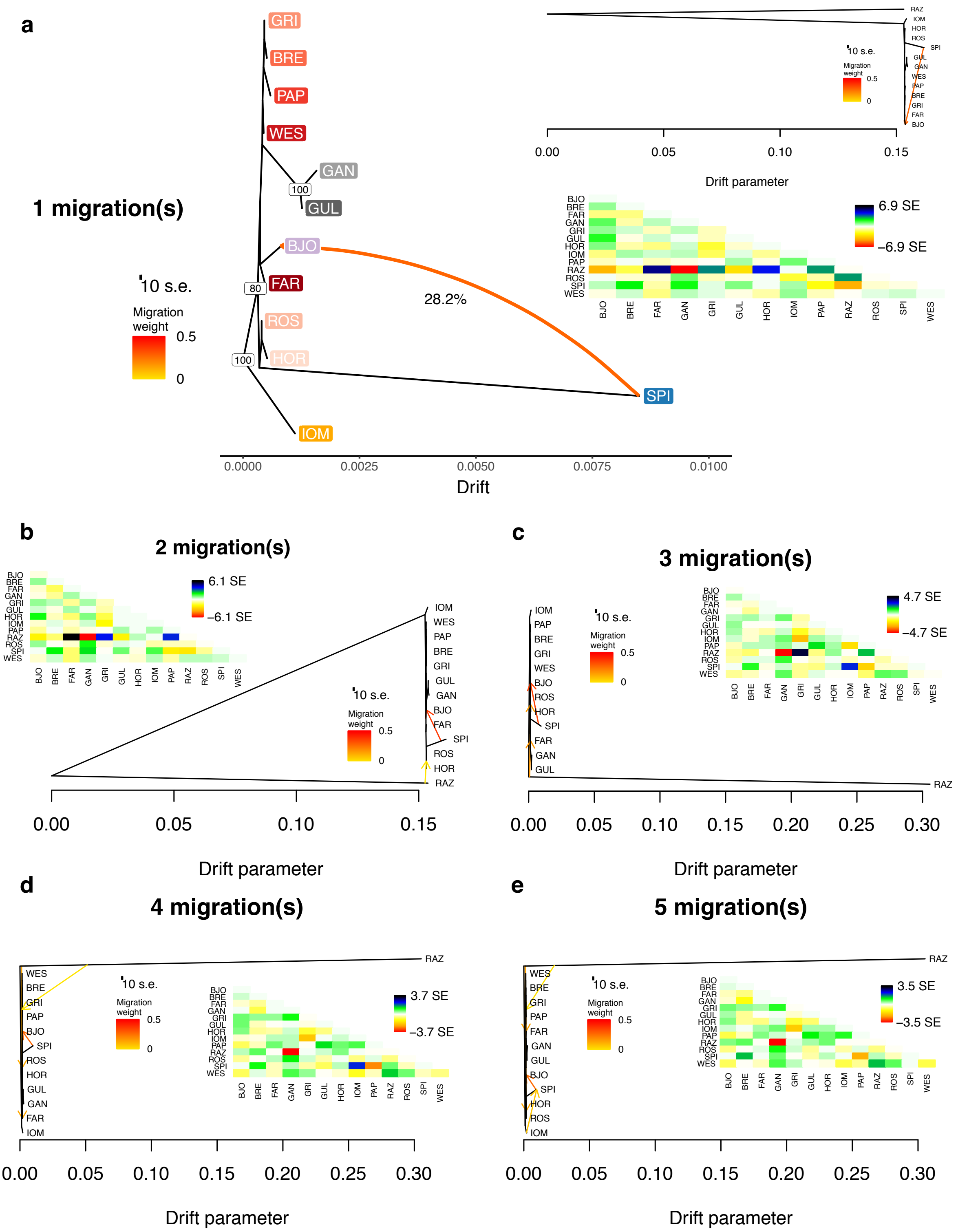

Figure S10: Population-based Treemix analyses of 12 Atlantic puffin colonies applying up to 5 migrations. The phylogenies were constructed using allele frequencies at 1,093,765 polymorphic nuclear sites and adding 1-5 (a-e) migration edges. Migrations are shown as arrows and their weight are indicated by a color range. Branch lengths are equivalent to a genetic drift parameter. The heatmaps highlight the residual fit of the tree displaying the standard error of the covariance between populations. All trees are rooted using the razorbill as an outgroup. Node labels in a) show bootstrap support ≥ 80.

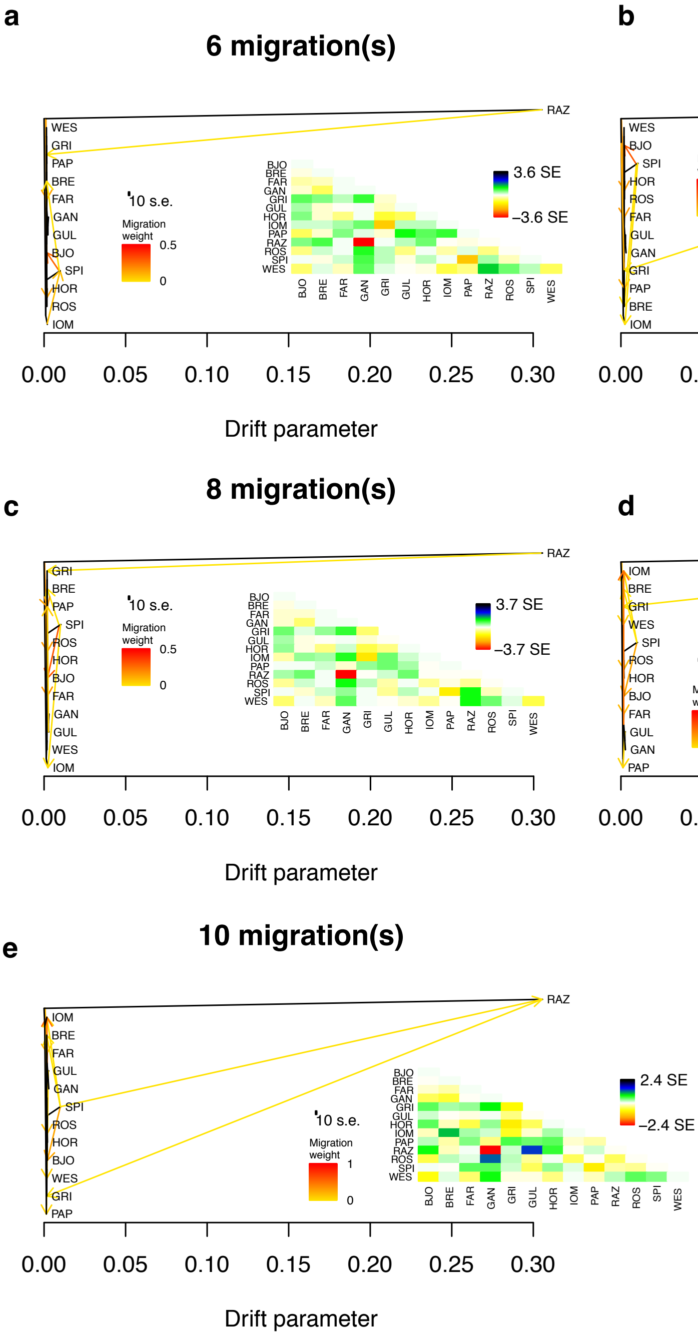

Figure S11: Population-based Treemix analyses of 12 Atlantic puffin colonies applying between 6 and 10 migrations. The phylogenies were constructed using allele frequencies at 1,093,765 polymorphic nuclear sites and adding 6-10 (a-e) migration edges. Migrations are shown as arrows and their weight are indicated by a color range. Branch lengths are equivalent to a genetic drift parameter. The heatmaps highlight the residual fit of the tree displaying the standard error of the covariance between populations. All trees are rooted using the razorbill as an outgroup.

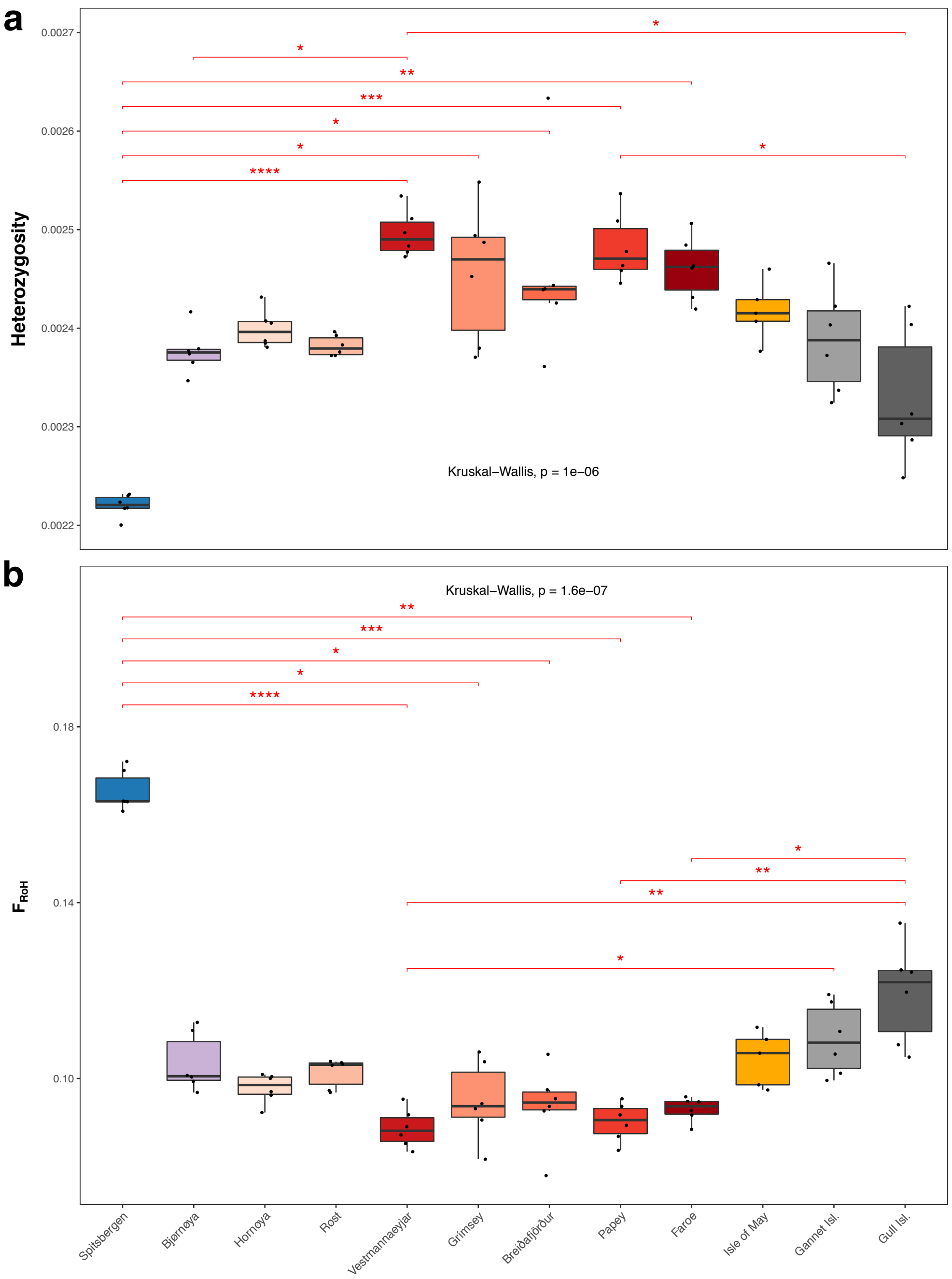

Figure S12: Global genome-wide heterozygosity and inbreeding compared between 12 Atlantic puffin colonies across the species' breeding range. a) Estimates of individual global genome-wide heterozygosity were based on the per-sample one-dimensional Site Frequency Spectrum calculated in ANGSD. b) Individual inbreeding coefficients, F_{RoH} , were defined as the fraction of the individual genomes filling into RoHs of a minimum length of 150 kbp. RoHs were declared as all regions with at least two subsequent 100 kbp windows harboring a heterozygosity below 1.435663 x 10^{-3}. In both plots, black dots indicate individual sample estimates and black lines the median per colony. Different colonies in both plots are indicated using different colors consistent with the remaining manuscript. Statistical significance of differences between populations was assessed with global Kruskal-Wallis tests, followed by *post-hoc* Dunn tests applying the Holm correction (n=12). Significant differences are indicated in red. *p<0.05, **p<0.01, ***p<0.001, ****p<0.0001. Error bars show range of values within 1.5 times the interquartile range.

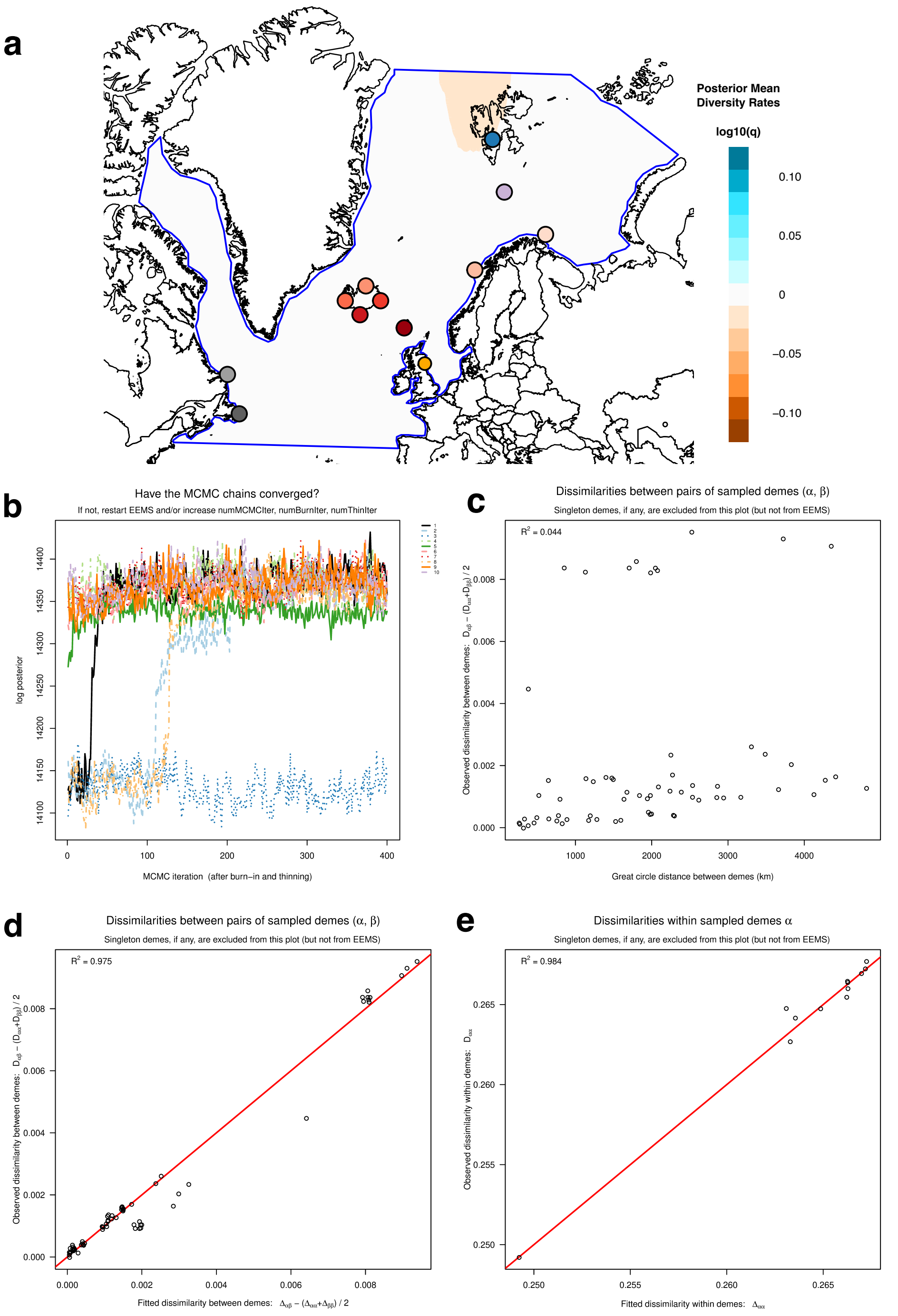

Figure S13: Graphical output of EEMS analysis. a) Posterior diversity rates plot depicts deviations from diversity estimates associated with continuous long-distance gene flow. Darker reds indicate reduced diversity across those areas, and darker blues indicate higher diversity rates than expected. Different colonies are indicated using different colors consistent with the remaining manuscript. b) Posterior probability trace plot of the ten MCMC chains providing an indication on the convergence. Each chain was started from a different randomly initialized parameter state and one chain converged at a local as opposed to the global maximum. c) Genetic dissimilarities as calculated by the EEMS analysis vs. geographic distance between demes/colonies. The two remaining plots show the observed vs. fitted genetic dissimilarities as calculated by the EEMS model d) between and e) within demes/colonies. The red line indicates a fit of 100% between observed and model values. The R^2 value in c), d), and e) describes the fit of the correlation.

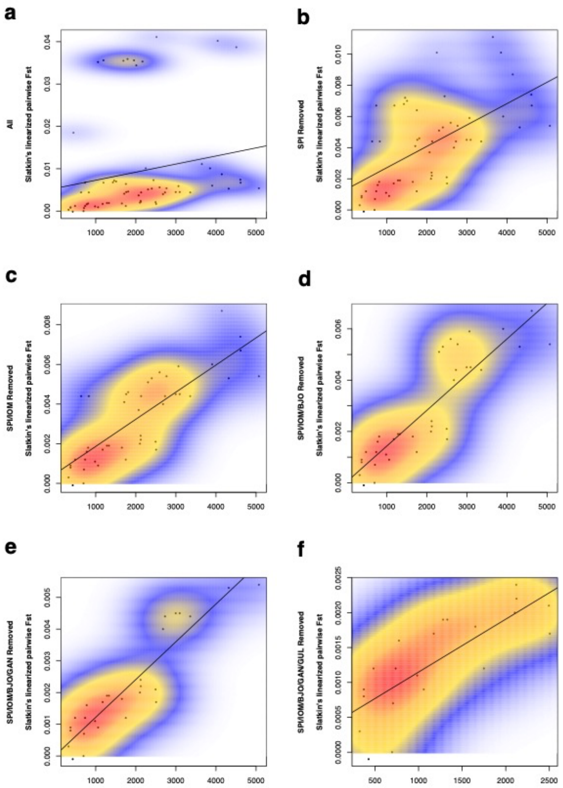

Figure S14: Isolation by distance (IBD) patterns for 12 Atlantic puffin populations. Genetic (Slatkin's linearized F_{ST}) distance as a function of geographic (Least Cost Path - only over water) distance (x-axes unit is kilometer) is presented for various colony subsets. The diagonal lines visualize the results of the Multiple Regression on Distance Matrices (MRM) analysis (slope and y-intercept). a) Using all colonies ($n_{Colonies}$ = 12), the Mantel test between genetic and geographic distance (*R* = 0.192, *P* = 0.172) was not significant and 3.69% of variance in Slatkin's linearized F_{ST} was explained by geographic distance (regression coefficient of linear IBD model = 1.91×10^{-6}, *P* = 0.354). b) After removing the Spitsbergen colony ($n_{Colonies}$ = 11), the Mantel test between genetic and geographic distance (*R* = 0.613, *P* = 0.002) was significant and 37.58% of variance in Slatkin's linearized F_{ST} was explained by geographic distance (regression coefficient of linear IBD model = 1.37×10^{-6}, *P* = 0.003). c) Without the Spitsbergen and Isle of May colony ($n_{Colonies}$ = 10), the Mantel test between genetic and geographic distance (*R* = 0.812, *P* = 0.001) was significant and 65.92% of variance in Slatkin's linearized F_{ST} was explained by geographic distance (regression coefficient of linear IBD model = 1.37×10^{-6}, *P* = 0.002). d) After removing the Spitsbergen, Isle of May and Bjørnøya colonies ($n_{Colonies}$ = 9), the Mantel test between genetic and geographic distance (*R* = 0.880, *P* = 0.001) was significant and 77.49% of variance in Slatkin's linearized F_{ST} was explained by geographic distance (regression coefficient of linear IBD model = 1.39×10^{-6}, *P* = 0.002). e) Without the Spitsbergen, Isle of May, Bjørnøya and Gannet Island colonies ($n_{Colonies}$ = 8), the Mantel test between genetic and geographic distance (*R* = 0.922, *P* = 0.002) was significant and 84.98% of variance in Slatkin's linearized F_{ST} was explained by geographic distance (regression coefficient of linear IBD model = 1.19×10^{-6}, *P* = 0.001). f) After removing the Spitsbergen, Isle of May, Bjørnøya and Canadian colonies ($n_{Colonies}$ = 7), the Mantel test between genetic and geographic distance (*R* = 0.775, *P* = 0.012) was significant and 60.08% of variance in Slatkin's linearized F_{ST} was explained by geographic distance (regression coefficient of linear IBD model = 0.76×10^{-6}, *P* = 0.006). A two-dimensional kernel density estimation (kde2d) highlights dense groups of data points in all subplots, thus substructure in the genomic landscape pattern. Analyses were conducted and results visualized in R using the *ecodist*, *marmap* and *MASS* packages.

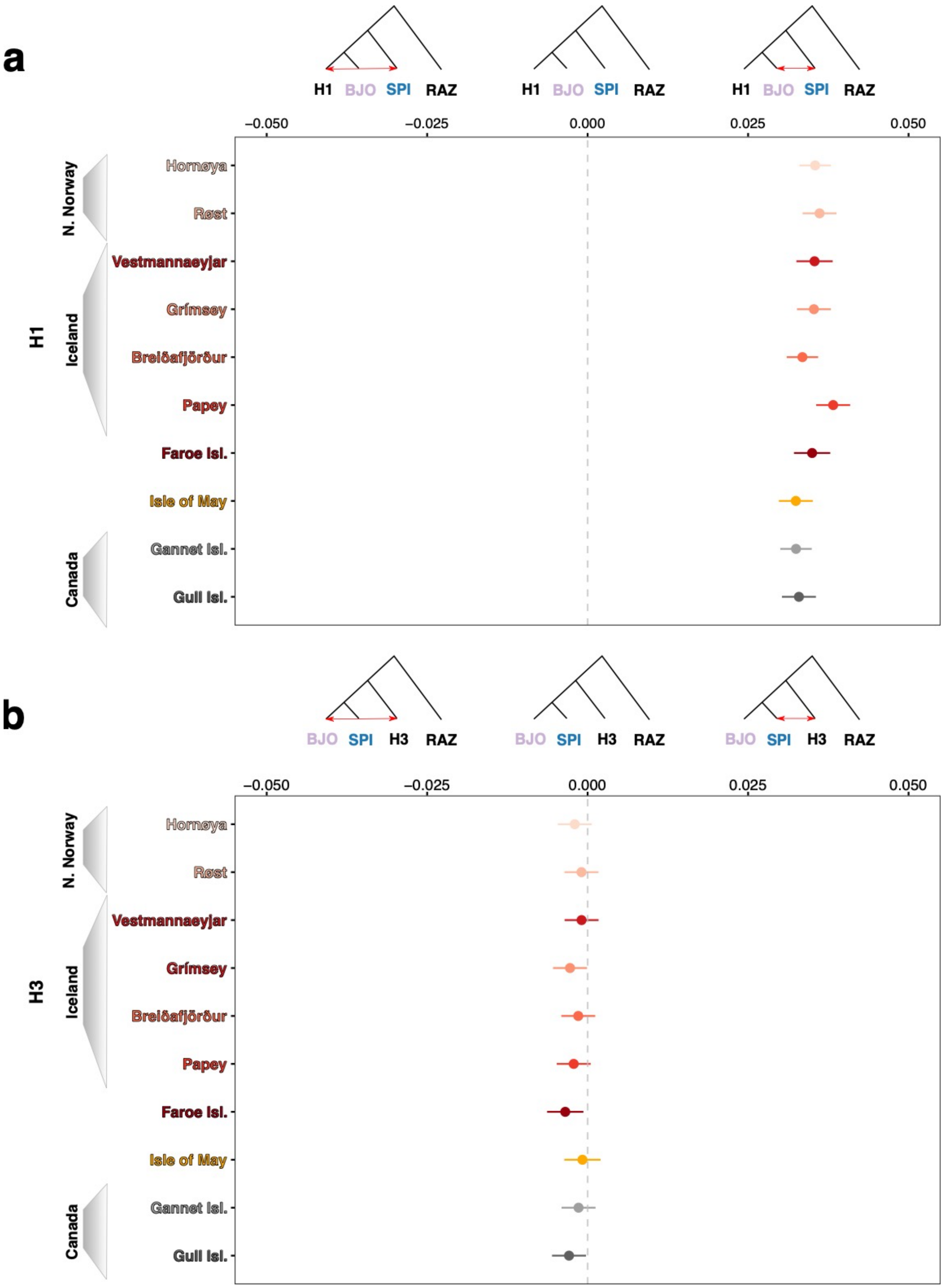

Figure S15: D-statistics for two topologies involving the Spitsbergen and Bjørnøya colonies. The D-statistic (ABBABABA test) was calculated for the tree configuration a) (((H1, BJO),SPI),RAZ) and b) (((BJO, SPI),H3),RAZ) using the razorbill as the outgroup. The error bars indicate one standard deviation of the D-statistic. The grey dashed line presents the null expectation of no excess gene flow between a) Bjørnøya/H1 and Spitsbergen and b) Bjørnøya/Spitsbergen and H3.

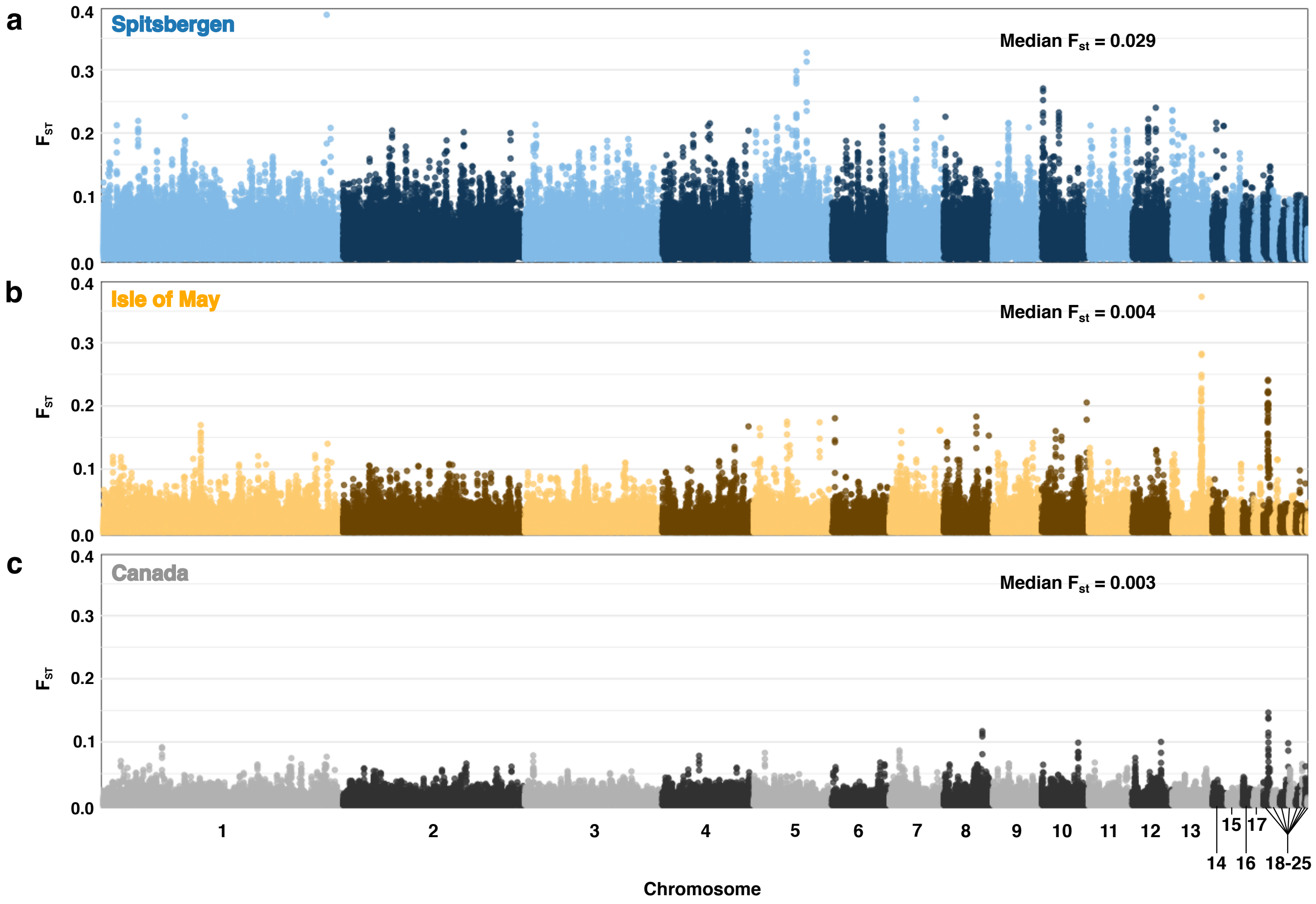

Figure S16: Genome-wide analysis of pairwise F_{ST} between four genomic population clusters of the Atlantic puffin. Estimates of pairwise F_{ST} were calculated between the Norway/Iceland/Faroe cluster and the a) Spitsbergen, b) Isle of May, and c) Canada clusters in 50 kb sliding windows with a 12.5 kb shift along the 25 pseudo-chromosomes. Estimates are based on two-dimensional Site Frequency Spectra calculated in ANGSD. The different chromosomes are represented by different shades following the cluster color used throughout the main manuscript.

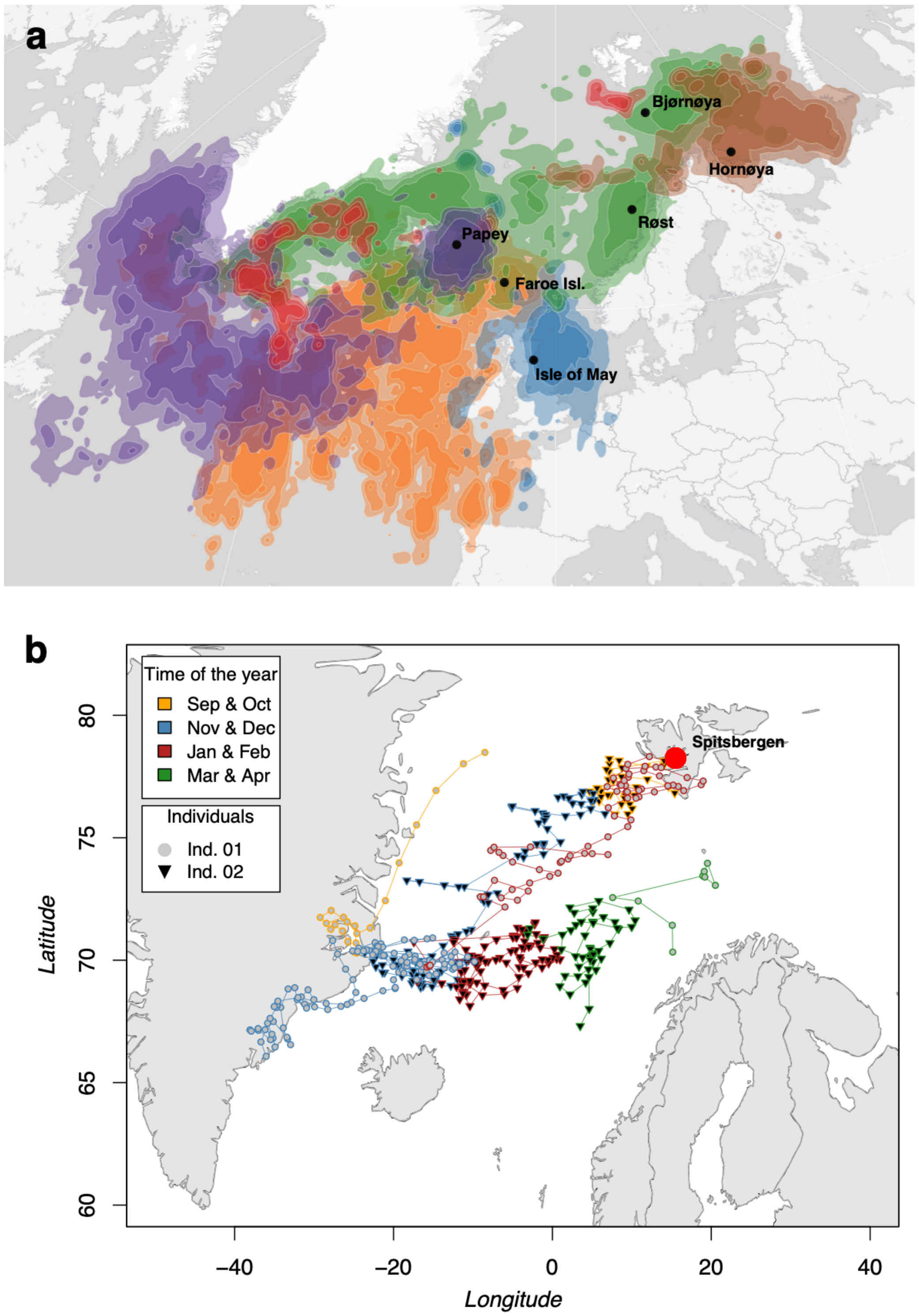

Figure S17: Overwintering distribution of several Atlantic puffin colonies spread across the species' breeding range. The data for the non-breeding distribution of the six colonies highlighted in a) and sampled in this study was made available by SEATRACK (http://www.seapop.no/en/seatrack/) and collected between 2009-2019 in the months of August-April using GLS loggers. Bjørnøya = red, Hornøya = brown, Røst = green, Papey = purple, Faroe Isl. = orange, and Isle of May = blue**.** b) Distributional data of two Spitsbergen individuals collected in 2018-2019 using GLS loggers.

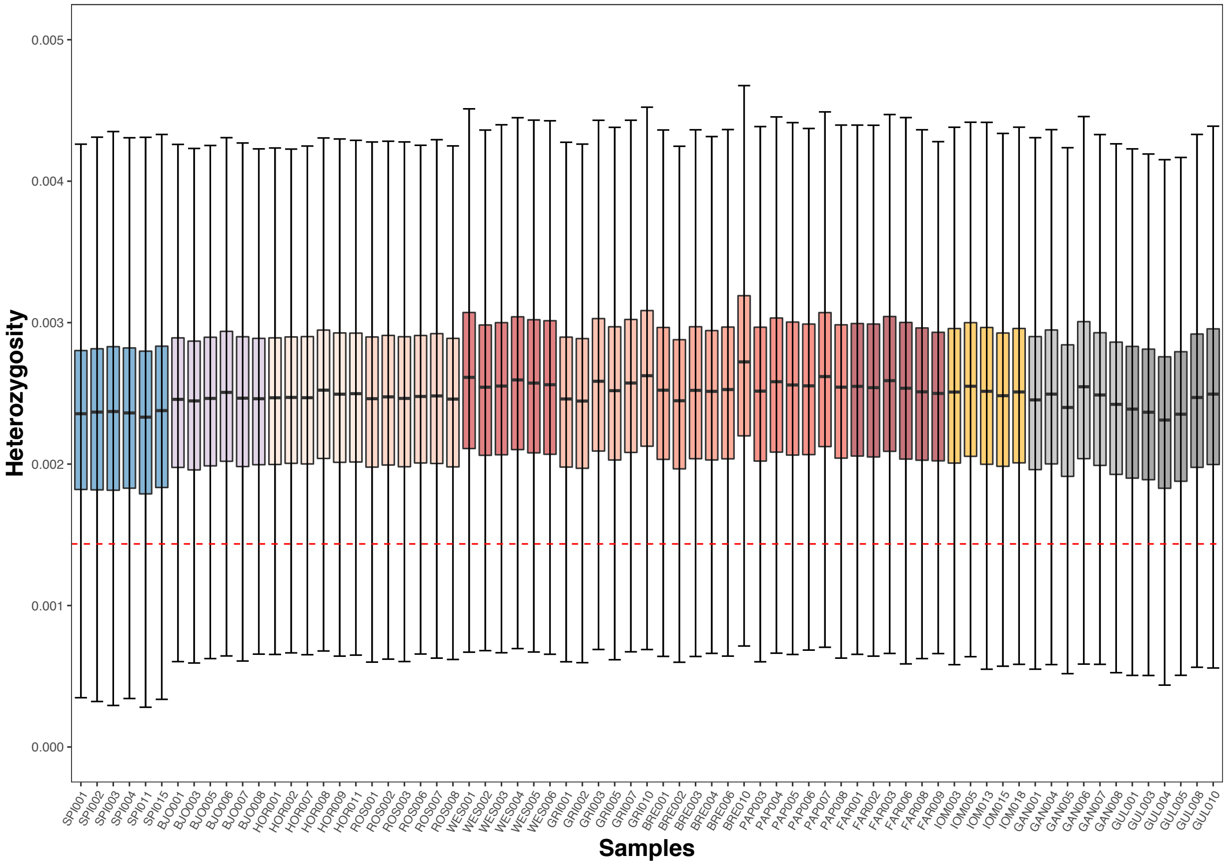

Figure S18: Local genome-wide heterozygosity compared between 71 Atlantic puffin individuals across 12 colonies throughout the species' breeding range. Estimates of individual genome-wide heterozygosity are based on local estimates of heterozygosity in 100 kbp sliding windows with a 50 kbp shift along the 25 pseudo-chromosomes. Local estimates per window are based on one-dimensional Site Frequency Spectra calculated in ANGSD. The dashed red line indicates the 10% quantile of the average local heterozygosity across all samples (1.435663 x 10^{-3}). Black lines present the median per sample. Error bars show range of values within 1.5 times the interquartile range. Different colonies are indicated using different colors consistent with the remaining manuscript.

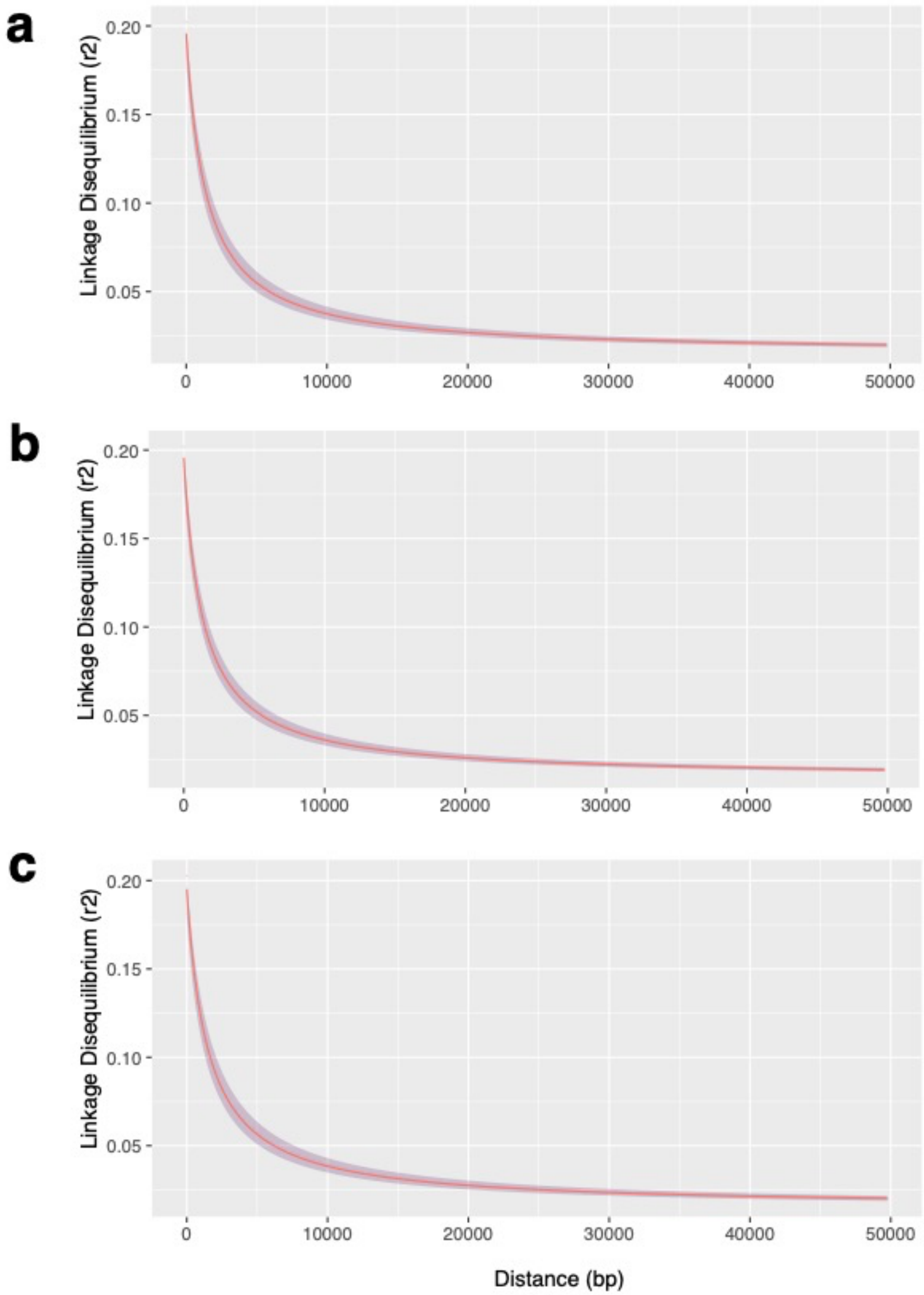

Figure S19: Linkage Disequilibrium (LD) decay across the three longest pseudo-chromosomes of the Atlantic puffin draft reference genome. Linkage expressed as the r^2 value was calculated for pairs of sites within 50 kb windows along pseudo-chromosomes a) 1, b) 2, and c) 3 using ngsLD. The shaded grey area indicates 95% confidence intervals. To visualize the results, the R script *fit_LDdecay.R* (supplied with ngsLD) was run with *--max_kb_dist 100 --fit_boot 100 --fit_level 20 --plot_data --plot_scale 3*

5. References

1. Weisenfeld, N. I., Kumar, V., Shah, P., Church, D. M. & Jaffe, D. B. Direct determination of diploid genome sequences. *Genome Res.* **27**, 757–767 (2017).
2. Taylor, G. A. *et al.* The genome of the North American brown bear or grizzly: Ursus arctos ssp. horribilis. *Genes* **9**, (2018).
3. Kwan, H. H. *et al.* The genome of the Steller sea lion (Eumetopias jubatus). *Genes* **10**, (2019).
4. Jones, S. J. *et al.* The genome of the northern sea otter (Enhydra lutris kenyoni). *Genes* **8**, (2017).
5. Jones, S. J. M. *et al.* The genome of the beluga whale (Delphinapterus leucas). *Genes* **8**, (2017).
6. Roach, M. J., Schmidt, S. A. & Borneman, A. R. Purge Haplotigs: allelic contig reassignment for third-gen diploid genome assemblies. *BMC Bioinformatics* **19**, 460 (2018).
7. Jackman, S. D. *et al.* Tigmint: correcting assembly errors using linked reads from large molecules. *BMC Bioinformatics* **19**, 393 (2018).
8. Coombe, L. *et al.* ARKS: chromosome-scale scaffolding of human genome drafts with linked read kmers. *BMC Bioinformatics* **19**, 234 (2018).
9. Warren, R. L. *et al.* LINKS: Scalable, alignment-free scaffolding of draft genomes with long reads. *Gigascience* **4**, 35 (2015).
10. Simão, F. A., Waterhouse, R. M., Ioannidis, P., Kriventseva, E. V. & Zdobnov, E. M. BUSCO: assessing genome assembly and annotation completeness with single-copy orthologs. *Bioinformatics* **31**, 3210–3212 (2015).
11. Gurevich, A., Saveliev, V., Vyahhi, N. & Tesler, G. QUAST: quality assessment tool for genome assemblies. *Bioinformatics* **29**, 1072–1075 (2013).
12. Paulino, D. *et al.* Sealer: a scalable gap-closing application for finishing draft genomes. *BMC Bioinformatics* **16**, 230 (2015).
13. Warren, R. L. *et al.* ntEdit: scalable genome sequence polishing. *Bioinformatics* (2019) doi:10.1093/bioinformatics/btz400.
14. Wood, D. E. & Salzberg, S. L. Kraken: ultrafast metagenomic sequence classification using exact alignments. *Genome Biol.* **15**, R46 (2014).
15. Laetsch, D. R. & Blaxter, M. L. BlobTools: Interrogation of genome assemblies. *F1000Res.* **6**, (2017).

16. Waterhouse, A. M., Procter, J. B., Martin, D. M. A., Clamp, M. & Barton, G. J. Jalview Version 2—a multiple sequence alignment editor and analysis workbench. *Bioinformatics* **25**, 1189–1191 (2009).
17. Shen, W., Le, S., Li, Y. & Hu, F. SeqKit: A cross-platform and ultrafast toolkit for FASTA/Q file manipulation. *PLoS One* **11**, e0163962 (2016).
18. Bernt, M. *et al.* MITOS: improved de novo metazoan mitochondrial genome annotation. *Mol. Phylogenet. Evol.* **69**, 313–319 (2013).
19. Al Arab, M. *et al.* Accurate annotation of protein-coding genes in mitochondrial genomes. *Mol. Phylogenet. Evol.* **106**, 209–216 (2017).
20. Lowe, T. M. & Chan, P. P. tRNAscan-SE On-line: integrating search and context for analysis of transfer RNA genes. *Nucleic Acids Res.* **44**, W54–7 (2016).
21. Yu, Y., Ouyang, Y. & Yao, W. shinyCircos: an R/Shiny application for interactive creation of Circos plot. *Bioinformatics* **34**, 1229–1231 (2018).
22. Li, H. Minimap2: pairwise alignment for nucleotide sequences. *Bioinformatics* **34**, 3094–3100 (2018).
23. Campos, P. F. & Gilbert, T. M. P. DNA extraction from keratin and chitin. in *Ancient DNA: Methods and Protocols* (eds. Shapiro, B. & Hofreiter, M.) 43–49 (Humana Press, 2012).
24. Griffiths, R., Double, M. C., Orr, K. & Dawson, R. J. A DNA test to sex most birds. *Mol. Ecol.* **7**, 1071–1075 (1998).
25. Bantock, T. M., Prys-Jones, R. P. & Lee, P. L. M. New and improved molecular sexing methods for museum bird specimens. *Mol. Ecol. Resour.* **8**, 519–528 (2008).
26. Schubert, M. *et al.* Characterization of ancient and modern genomes by SNP detection and phylogenomic and metagenomic analysis using PALEOMIX. *Nat. Protoc.* **9**, 1056–1082 (2014).
27. Lindgreen, S. AdapterRemoval: easy cleaning of next-generation sequencing reads. *BMC Res. Notes* **5**, 337 (2012).
28. Li, H. Aligning sequence reads, clone sequences and assembly contigs with BWA-MEM. *arXiv preprint arXiv:1303.3997* (2013).
29. Broad Institute. Picard Toolkit. http://broadinstitute.github.io/picard/.
30. McKenna, A. *et al.* The Genome Analysis Toolkit: a MapReduce framework for analyzing next-generation DNA sequencing data. *Genome Res.* **20**, 1297–1303 (2010).
31. Li, H. *et al.* The sequence alignment/map format and SAMtools. *Bioinformatics* **25**, 2078–2079 (2009).

32. Van der Auwera, G. A. *et al.* From FastQ data to high confidence variant calls: the Genome Analysis Toolkit best practices pipeline. *Curr. Protoc. Bioinformatics* **43**, 11.10.1–33 (2013).
33. Cingolani, P. *et al.* A program for annotating and predicting the effects of single nucleotide polymorphisms, SnpEff: SNPs in the genome of Drosophila melanogaster strain w1118; iso-2; iso-3. *Fly* **6**, 80–92 (2012).
34. Edgar, R. C. MUSCLE: multiple sequence alignment with high accuracy and high throughput. *Nucleic Acids Res.* **32**, 1792–1797 (2004).
35. Kalyaanamoorthy, S., Minh, B. Q., Wong, T. K. F., von Haeseler, A. & Jermiin, L. S. ModelFinder: fast model selection for accurate phylogenetic estimates. *Nat. Methods* **14**, 587–589 (2017).
36. Lanfear, R., Calcott, B., Ho, S. Y. W. & Guindon, S. Partitionfinder: combined selection of partitioning schemes and substitution models for phylogenetic analyses. *Mol. Biol. Evol.* **29**, 1695–1701 (2012).
37. Chernomor, O., von Haeseler, A. & Minh, B. Q. Terrace aware data structure for phylogenomic inference from supermatrices. *Syst. Biol.* **65**, 997–1008 (2016).
38. Gadagkar, S. R., Rosenberg, M. S. & Kumar, S. Inferring species phylogenies from multiple genes: concatenated sequence tree versus consensus gene tree. *J. Exp. Zool. B Mol. Dev. Evol.* **304**, 64–74 (2005).
39. Matschiner, M. Fitchi: haplotype genealogy graphs based on the Fitch algorithm. *Bioinformatics* **32**, 1250–1252 (2016).
40. Excoffier, L. & Lischer, H. E. L. Arlequin suite ver 3.5: a new series of programs to perform population genetics analyses under Linux and Windows. *Mol. Ecol. Resour.* **10**, 564–567 (2010).
41. Tajima, F. Statistical method for testing the neutral mutation hypothesis by DNA polymorphism. *Genetics* **123**, 585–595 (1989).
42. Watterson, G. A. Heterosis or neutrality? *Genetics* **85**, 789–814 (1977).
43. Chakraborty, R. Mitochondrial DNA polymorphism reveals hidden heterogeneity within some Asian populations. *Am. J. Hum. Genet.* **47**, 87–94 (1990).
44. Fu, Y. X. Statistical tests of neutrality of mutations against population growth, hitchhiking and background selection. *Genetics* **147**, 915–925 (1997).
45. Korneliussen, T. S., Albrechtsen, A. & Nielsen, R. ANGSD: Analysis of next generation sequencing data. *BMC Bioinformatics* **15**, 356 (2014).
46. R Core Team. R: A language and environment for statistical computing. (2020).

47. Fox, E. A., Wright, A. E., Fumagalli, M. & Vieira, F. G. ngsLD: evaluating linkage disequilibrium using genotype likelihoods. *Bioinformatics* **35**, 3855–3856 (2019).
48. Van Dongen, S. Graph clustering via a discrete uncoupling process. *SIAM J. Matrix Anal. Appl.* **30**, 121–141 (2008).
49. Li, L., Stoeckert, C. J., Jr & Roos, D. S. OrthoMCL: identification of ortholog groups for eukaryotic genomes. *Genome Res.* **13**, 2178–2189 (2003).
50. Orlando, L. & Librado, P. Origin and evolution of deleterious mutations in horses. *Genes* **10**, (2019).
51. Meisner, J. & Albrechtsen, A. Inferring population structure and admixture proportions in low-depth NGS data. *Genetics* **210**, 719–731 (2018).
52. Skotte, L., Korneliussen, T. S. & Albrechtsen, A. Estimating individual admixture proportions from next generation sequencing data. *Genetics* **195**, 693–702 (2013).
53. Kopelman, N. M., Mayzel, J., Jakobsson, M., Rosenberg, N. A. & Mayrose, I. Clumpak: a program for identifying clustering modes and packaging population structure inferences across K. *Mol. Ecol. Resour.* **15**, 1179–1191 (2015).
54. Evanno, G., Regnaut, S. & Goudet, J. Detecting the number of clusters of individuals using the software STRUCTURE: a simulation study. *Mol. Ecol.* **14**, 2611–2620 (2005).
55. Lefort, V., Desper, R. & Gascuel, O. FastME 2.0: A comprehensive, accurate, and fast distance-based phylogeny inference program. *Mol. Biol. Evol.* **32**, 2798–2800 (2015).
56. Nguyen, L.-T., Schmidt, H. A., von Haeseler, A. & Minh, B. Q. IQ-TREE: a fast and effective stochastic algorithm for estimating maximum-likelihood phylogenies. *Mol. Biol. Evol.* **32**, 268–274 (2015).
57. Pickrell, J. K. & Pritchard, J. K. Inference of population splits and mixtures from genome-wide allele frequency data. *PLoS Genet.* **8**, e1002967 (2012).
58. Matz, M. V. Fantastic Beasts and How To Sequence Them: Ecological Genomics for Obscure Model Organisms. *Trends Genet.* **34**, 121–132 (2018).
59. Mann, H. B. & Whitney, D. R. On a test of whether one of two random variables is stochastically larger than the other. *Ann. Math. Stat.* **18**, 50–60 (1947).
60. Holm, S. A simple sequentially rejective multiple test procedure. *Scand. Stat. Theory Appl.* **6**, 65–70 (1979).
61. Kruskal, W. H. & Wallis, W. A. Use of ranks in one-criterion variance analysis. *J. Am. Stat. Assoc.* **47**, 583–621 (1952).
62. Dunn, O. J. Multiple comparisons using rank sums. *Technometrics* **6**, 241–252 (1964).

63. Sánchez-Barreiro, F. *et al.* Historical population declines prompted significant genomic erosion in the northern and southern white rhinoceros (Ceratotherium simum). *bioRxiv* 2020.05.10.086686 (2020) doi:10.1101/2020.05.10.086686.
64. Petkova, D., Novembre, J. & Stephens, M. Visualizing spatial population structure with estimated effective migration surfaces. *Nat. Genet.* **48**, 94–100 (2016).
65. Mantel, N. The detection of disease clustering and a generalized regression approach. *Cancer Res.* **27**, 209–220 (1967).
66. Lichstein, J. W. Multiple regression on distance matrices: a multivariate spatial analysis tool. *Plant Ecol.* **188**, 117–131 (2007).
67. Slatkin, M. A measure of population subdivision based on microsatellite allele frequencies. *Genetics* **139**, 457–462 (1995).
68. Pante, E., Simon-Bouhet, B. & Irisson, J.-O. *marmap - R package*. (2019).
69. Goslee, S. & Urban, D. The ecodist package for dissimilarity-based analysis of ecological data. *Journal of Statistical Software, Articles* **22**, 1–19 (2007).
70. Venables, W. N. & Ripley, B. D. *Modern Applied Statistics with S*. (Springer, 2002).
71. Legendre, P. & Anderson, M. J. Distance-based redundancy analysis: Testing multispecies responses in multifactorial ecological experiments. *Ecol. Monogr.* **69**, 1–24 (1999).
72. Cailliez, F. The analytical solution of the additive constant problem. *Psychometrika* **48**, 305–308 (1983).
73. Dray, S., Legendre, P. & Peres-Neto, P. R. Spatial modelling: a comprehensive framework for principal coordinate analysis of neighbour matrices (PCNM). *Ecol. Modell.* **196**, 483–493 (2006).
74. Borcard, D. & Legendre, P. All-scale spatial analysis of ecological data by means of principal coordinates of neighbour matrices. *Ecol. Modell.* **153**, 51–68 (2002).
75. Dray, S. *et al. adespatial: Multivariate Multiscale Spatial Analysis. R package version 0.3-13. https://CRAN.R-project.org/package=adespatial*. (2021).
76. Anker-Nilssen, T., Aarvak, T. & Bangjord, G. Mass mortality of Atlantic Puffins Fratercula arctica off Central Norway, spring 2002: causes and consequences. *Atl. Seabirds* **5**, 57–72 (2003).
77. Rayner, N. A. Global analyses of sea surface temperature, sea ice, and night marine air temperature since the late nineteenth century. *J. Geophys. Res.* **108**, (2003).
78. Blanchet, F. G., Legendre, P. & Borcard, D. Modelling directional spatial processes in ecological data. *Ecol. Modell.* **215**, 325–336 (2008).

79. Benestan, L. M. *et al.* Population genomics and history of speciation reveal fishery management gaps in two related redfish species (Sebastes mentella and Sebastes fasciatus). *Evol. Appl.* **14**, 588–606 (2021).
80. Soraggi, S., Wiuf, C. & Albrechtsen, A. Powerful inference with the D-statistic on low-coverage whole-genome data. *G3* **8**, 551–566 (2018).
