# Supplementary material for: Complex population structure of the Atlantic puffin revealed by whole genome analyses
Source: Commun Biol. 2021 Jul 29;4:922. doi: 10.1038/s42003-021-02415-4 (PMC8322098; doi:10.1038/s42003-021-02415-4)
Supplement: Supplementary file 2 — Reporting Summary [file 42003_2021_2415_MOESM2_ESM.pdf]

nature research

Corresponding author(s): Oliver Kersten, Sanne Boessenkool

Last updated by author(s): Jun 2, 2021

Reporting Summary

Nature Research wishes to improve the reproducibility of the work that we publish. This form provides structure for consistency and transparency in reporting. For further information on Nature Research policies, see our Editorial Policies and the Editorial Policy Checklist.

Statistics

For all statistical analyses, confirm that the following items are present in the figure legend, table legend, main text, or Methods section.

n/a	Confirmed	
☐	☒	The exact sample size (*n*) for each experimental group/condition, given as a discrete number and unit of measurement
☐	☒	A statement on whether measurements were taken from distinct samples or whether the same sample was measured repeatedly
☐	☒	The statistical test(s) used AND whether they are one- or two-sided<br>*Only common tests should be described solely by name; describe more complex techniques in the Methods section.*
☐	☒	A description of all covariates tested
☐	☒	A description of any assumptions or corrections, such as tests of normality and adjustment for multiple comparisons
☐	☒	A full description of the statistical parameters including central tendency (e.g. means) or other basic estimates (e.g. regression coefficient) AND variation (e.g. standard deviation) or associated estimates of uncertainty (e.g. confidence intervals)
☐	☒	For null hypothesis testing, the test statistic (e.g. *F*, *t*, *r*) with confidence intervals, effect sizes, degrees of freedom and *P* value noted<br>*Give P values as exact values whenever suitable.*
☒	☐	For Bayesian analysis, information on the choice of priors and Markov chain Monte Carlo settings
☒	☐	For hierarchical and complex designs, identification of the appropriate level for tests and full reporting of outcomes
☒	☐	Estimates of effect sizes (e.g. Cohen's *d*, Pearson's *r*), indicating how they were calculated

Our web collection on statistics for biologists contains articles on many of the points above.

Software and code

Policy information about availability of computer code

Data collection	No software was used for data collection.
Data analysis	Supernova assembler v2.1.1, "purge_haplotigs", BLASTn, Tigmint, ARKS, LINKS v1.8.6, BUSCO v3, QUAST v4.6, Sealer, ntCard/ntHits/ntEdit, Kraken2, Blobtools, Jalview v2.11.1, SeqKit v0.12.0, tRNAscan-SE, shinyCircos, minimap2 v2.17, PALEOMIX v1.2.14, AdapterRemoval v2.3.1, BWA mem v0.7.17, PicardTools v2.18.27, GATK v4.1.4, SAMtools v1.9, snpEff, BCFtools v1.9, Muscle v3.8.31, ModelFinder, IQTree v1.6.12, Fitchi, Arlequin v3.5, ANGSD v0.931, R v3.6, ngsLD, OrthoMCL v2.0.92, PCAngsd v0.982, ngsAdmix v32, CLUMPAK, ngsDist v1.0.8, FastMe v2.1.5, Treemix v1.13, EEMS, Remaining code used for the population genomic analyses is available on the first author's GitHub (https://github.com/OKersten/PuffPopGen)

For manuscripts utilizing custom algorithms or software that are central to the research but not yet described in published literature, software must be made available to editors and reviewers. We strongly encourage code deposition in a community repository (e.g. GitHub). See the Nature Research guidelines for submitting code & software for further information.

Data

Policy information about availability of data

All manuscripts must include a data availability statement. This statement should provide the following information, where applicable:

- Accession codes, unique identifiers, or web links for publicly available datasets
- A list of figures that have associated raw data
- A description of any restrictions on data availability

Raw read data have been deposited in the European Nucleotide Archive (ENA, www.ebi.ac.uk/ena) under study accession number PRJEB40631 (see Table S4 for individual sample accession numbers). Nuclear and mitochondrial scaffolds (GCA_905066775.1, CAJHIB010000001-CAJHIB010013329), as well as pseudo-chromosomes (GCA_905066775.2, CAJHIB020000001-CAJHIB020000027), have been uploaded to ENA (Project PRJEB40926, Sample SAMEA7482542).

Field-specific reporting

Please select the one below that is the best fit for your research. If you are not sure, read the appropriate sections before making your selection.

☐ Life sciences ☐ Behavioural & social sciences ☒ Ecological, evolutionary & environmental sciences

For a reference copy of the document with all sections, see nature.com/documents/nr-reporting-summary-flat.pdf

Ecological, evolutionary & environmental sciences study design

All studies must disclose on these points even when the disclosure is negative.

Study description	The study presents the first whole genome analysis of structure, gene flow and taxonomy of a pelagic, North Atlantic seabird, the Atlantic puffin. It generated a de novo draft assembly for the Atlantic puffin and elucidates the population structure of the Atlantic puffin by whole genome resequencing of 72 individuals across 12 colonies representing the majority of the species' breeding range.
Research sample	The research sample included 72 adult Atlantic puffins (Fratercula arctica) across 12 colonies located in Svalbard, Northern mainland Norway, Iceland, the Faroe Islands, Scotland, and Canada. This set of samples covers the majority of the species' breeding range, and includes representatives of each of the three presumptive subspecies (F. arctica arctica, grabae, naumanni). Half of the samples were males and the other half females.
Sampling strategy	Sample size (six individuals per colony - 12 colonies), including an equal sex ratio (3 males and 3 females per colony) was chosen based on sample availability, financial budget, potential sex bias, and geographical extent of the breeding range.
Data collection	Feather and blood samples from Atlantic puffins were collected and made available by SEAPOP (http://www.seapop.no/en), SEATRACK (http://www.seapop.no/en/seatrack/) and ARCTOX (http://www.arctox.cnrs.fr/en/home). Genomic libraries were built by the Norwegian Sequencing Centre using a TruSeq DNA Nano preparation kit (Illumina).
Timing and spatial scale	Samples were collected between 2012-2018.
Data exclusions	An individual from the Isle of May was removed from the dataset due to low endogenous DNA content, low average depth of coverage and a large proportion of missing sites compared to all other samples.
Reproducibility	The entire code used for the population genomic analyses is available on the first author's GitHub (https://github.com/OKersten/PuffPopGen). It was rerun multiple times and repeating the analyses was successful.
Randomization	This is not relevant to the study. Samples were not randomized.
Blinding	Blinding was accomplished by having different personnel execute different stages of the sample acquisition and analysis. Many of the authors collected the samples, while the 1st author did the laboratory work. All sequence data was generated by the Norwegian Sequencing Center. Subsequently, the 1st author carried out the population genomic analyses with input from a few other authors.

Did the study involve field work? ☒ Yes ☐ No

Field work, collection and transport

Field conditions	Samples were collected at several Atlantic puffin colonies during ongoing monitoring by external programs. Puffin colonies are often located on grassy or rocky slopes and sea cliffs. Field conditions during sample collection in the spring and summer months varied considerably due to the large latitudinal and longitudinal range across the sampled colonies.
Location	The following colonies were sampled: Isle of May (Lat: 56.186559 Long: -2.557249), Grímsey (Lat: 66.544129 Long:-18.000131), Papey (Lat: 64.592281 Long: -14.167197), Breiðafjörður (Lat: 65.145486 Long: -22.807873), Vestmannaeyjar (Lat: 63.427217 Long: -20.267614), Faroe Islands (Lat: 62.144991 Long: -7.003334), Røst (Lat: 67.46205 Long: 11.937911), Bjørnøya (Lat: 74.452439 Long: 19.028405), Hornøya (Lat: 70.387488 Long: 31.157179), Gåsøyane (Lat: 78.454 Long: 16.2211), Gannet Isl. (Lat: 53.940044 Long: -56.563564), Gull Isl. (Lat: 47.238105 Long: -52.780226)
Access & import/export	Feather and blood samples from Atlantic puffins were collected and made available by SEAPOP (http://www.seapop.no/en), SEATRACK (http://www.seapop.no/en/seatrack/) and ARCTOX (http://www.arctox.cnrs.fr/en/home) using the appropriate permits for collection and import/export.<br>1. Gåsøyane, Røst, Hornøya, Bjørnøya (Norway) - FOTS ID #15602 and #15603 from the Norwegian Food Safety Authority for SEATRACK and SEAPOP ; Permit 2018/607 from Miljødirektoratet (Norwegian Environment Agency), dated 4 May 2018.<br>2. Gannet and Gull Island (Canada) - Canadian Wildlife Service Migratory Bird Banding Permit 10559 G, approved Animal Use Protocol (AUP) by Eastern Wildlife Animal Care Committee (17GR01, 18GR01), Newfoundland and Labrador Wilderness and Ecological Reserves Permit - Scientific Research (DOC/2017/02003), Canadian Wildlife Service Scientific Permit ST2785 (to MLM), Canadian Wildlife Service Banding Permit 10694, and Acadia University Animal Care Committee Permits ACC 02-15 and 06-15 (to MLM).<br>3. Isle of May (Scotland) - Scottish Natural Heritage licence 2014/MON/RP/156 and Ringing Permit A400 (to MPH).<br>4.Vestmannaeyjar, Papey, Breiðafjörður, Grímsey (Iceland) - Icelandic puffins were legally hunted during the hunting period of 1. July-15. August.<br>5. Faroe - Feathers came from predated birds collected in the field after the predator was finished with them

Disturbance	Disturbance to the individual puffins and entire colonies was minimized due to the sampling by trained staff with the appropriate permits following suitable procedures.

Reporting for specific materials, systems and methods

We require information from authors about some types of materials, experimental systems and methods used in many studies. Here, indicate whether each material, system or method listed is relevant to your study. If you are not sure if a list item applies to your research, read the appropriate section before selecting a response.

Materials & experimental systems

n/a	Involved in the study
☒	☐ Antibodies
☒	☐ Eukaryotic cell lines
☒	☐ Palaeontology and archaeology
☐	☒ Animals and other organisms
☒	☐ Human research participants
☒	☐ Clinical data
☒	☐ Dual use research of concern

Methods

n/a	Involved in the study
☒	☐ ChIP-seq
☒	☐ Flow cytometry
☒	☐ MRI-based neuroimaging

Animals and other organisms

Policy information about studies involving animals; ARRIVE guidelines recommended for reporting animal research

Laboratory animals	The study did not involve any laboratory animals.
Wild animals	Small blood or feather samples were collected from wild animals.
Field-collected samples	DNA was extracted from blood or feathers collected from Atlantic puffins. These samples were collected and made available by SEAPOP (http://www.seapop.no/en), SEATRACK (http://www.seapop.no/en/seatrack/) and ARCTOX (http://www.arctox.cnrs.fr/en/home) using the appropriate permits for collection.<br>1. Gåsøyane, Røst, Hornøya, Bjørnøya (Norway) - FOTS ID #15602 and #15603 from the Norwegian Food Safety Authority for SEATRACK and SEAPOP ; Permit 2018/607 from Miljødirektoratet (Norwegian Environment Agency), dated 4 May 2018.<br>2. Gannet and Gull Island (Canada) - Canadian Wildlife Service Migratory Bird Banding Permit 10559 G, approved Animal Use Protocol (AUP) by Eastern Wildlife Animal Care Committee (17GR01, 18GR01), Newfoundland and Labrador Wilderness and Ecological Reserves Permit - Scientific Research (DOC/2017/02003), Canadian Wildlife Service Scientific Permit ST2785 (to MLM), Canadian Wildlife Service Banding Permit 10694, and Acadia University Animal Care Committee Permits ACC 02-15 and 06-15 (to MLM).<br>3. Isle of May (Scotland) - Scottish Natural Heritage licence 2014/MON/RP/156 and Ringing Permit A400 (to MPH).<br>4.Vestmannaeyjar, Papey, Breiðafjörður, Grímsey (Iceland) - Icelandic puffins were legally hunted during the hunting period of 1. July-15. August.<br>5. Faroe - Feathers came from predated birds collected in the field after the predator was finished with them
Ethics oversight	These samples were collected and made available by SEAPOP (http://www.seapop.no/en), SEATRACK (http://www.seapop.no/en/seatrack/) and ARCTOX (http://www.arctox.cnrs.fr/en/home) using the appropriate permits for collection and following ethically approved guidelines (see above).

Note that full information on the approval of the study protocol must also be provided in the manuscript.
