# Supplementary material for: Complex population structure of the Atlantic puffin revealed by whole genome analyses
Source: Commun Biol. 2021 Jul 29;4:922. doi: 10.1038/s42003-021-02415-4 (PMC8322098; doi:10.1038/s42003-021-02415-4)
Supplement: Supplementary file 6 — Description of Additional Supplementary Files [file 42003_2021_2415_MOESM6_ESM.pdf]

Description of Additional Supplementary Files

File Name: Supplementary Data 1
Description: Refinement of an Atlantic Puffin draft genome assembled by the Supernova assembler using 10xGenomics. Presented are the bioinformatics analyses for the 4 most continuous and complete assemblies after the refinement pipeline consisting of 3 phases. Phases consisted of a) refining the two original Supernova assemblies (800M and 1000M) by using the reads from three Illumina HiSeqX lanes, b) increasing continuity and completeness by filling gaps, reducing missassemblies and removing contaminants, and c) final polishing. Joint settings used for each program are indicated in parentheses.

File Name: Supplementary Data 2
Description: Metadata including sexing results and mapping statistics associated with whole-genome resequencing of 72 Atlantic Puffin individuals across 12 colonies. Sequencing reads were mapped to the newly assembled draft genome using PALEOMIX. DNA extracts of individuals without information on sex were sexed with PCR. Raw read data have been deposited in the European Nucleotide Archive (ENA, www.ebi.ac.uk/ena) under the given accession numbers. M(Gender)=Male, F(Gender)=Female, B(Sample Type)=Blood, F(Sample Type)=Feather, Preserv.=Preservative, EtOH=Ethanol, LysBuff=Lysis Buffer, MT = mitochondrial. NU = nuclear.

File Name: Supplementary Data 3
Description: : Location and annotation of single nucleotide polymorphisms (SNPs) across the mitogenome of the Atlantic Puffin detected by sequencing 71 individuals across 12 colonies. Genotypes were jointly called with GATK v4.1.4 by using the HaplotypeCaller, CombineGVCFs and GenotypeGVCFs tool. Genotypes were filtered with BCFtools v1.9 according to GATKs Best Practices. Indels and non-biallelic SNPs were removed and only SNPs present in all individuals were kept.
